# The Piezo channel is central to the mechano-sensitive channel complex in the mammalian inner ear

**DOI:** 10.21203/rs.3.rs-2287052/v1

**Published:** 2023-07-12

**Authors:** Jeong Han Lee, Maria C. Perez-Flores, Seojin Park, Hyo Jeong Kim, Yingying Chen, Mincheol Kang, Jennifer Kersigo, Jinsil Choi, Phung N. Thai, Ryan Woltz, Dolores Columba Perez-Flores, Guy Perkins, Choong-Ryoul Sihn, Pauline Trinh, Xiao-Dong Zhang, Padmini Sirish, Yao Dong, Wayne Wei Feng, Isaac N. Pessah, Rose E. Dixon, Bernd Sokolowski, Bernd Fritzsch, Nipavan Chiamvimonvat, Ebenezer N. Yamoah

**Affiliations:** 1 Department of Physiology and Cell Biology, School of Medicine, University of Nevada, Reno, NV 89557; 2 Prestige Biopharma, 11-12F, 44, Myongjigukje7-ro, Gangseo-gu, Busan, South Korea 67264; 3 Department of Biology, University of Iowa, Iowa City, IA; 4 Division of Cardiovascular Medicine, Department of Internal Medicine, University of California, Davis, CA 95616.; 5 National Center for Microscopy and Imaging Research, University of California San Diego, La Jolla, CA 92093; 6 Department of Molecular Biosciences, School of Veterinary Medicine, University of California, Davis, 1089 VM3B, Davis, CA 95616; 7 Department of Physiology & Membrane Biology, Tupper Hall, One Shields Avenue, Davis, CA. 95616; 8 Department of Otolaryngology-Head and Neck Surgery, Morsani College of Medicine, University of South Florida, Tampa, FL,

## Abstract

The inner ear is the hub where hair cells transduce sound, gravity, and head acceleration stimuli carried by neural codes to the brain. Of all the senses, hearing and balance, which rely on mechanosensation, are the fastest sensory signals transmitted to the central nervous system. The mechanoelectrical transducer (MET) channel in hair cells is the entryway for the sound-balance-brain interface, but the channel’s composition has eluded biologists due to its complexity. Here, we report that the mouse utilizes Piezo1 (Pz1) and Piezo2 (Pz2) isoforms as central components of the MET complex. The Pz channel subunits are expressed in hair-cell stereocilia, are co-localized and co-assembled, and are essential components of the MET complex *in vitro* and *in situ,* including integration with the transmembrane channel (Tmc1/2) protein. Mice expressing non-functional *Pz1* and *Pz2,* but not functional *Pz1* at the ROSA26 locus under the control of hair-cell promoters, have impaired auditory and vestibular traits that can only be explained if Pz channel multimers are integral to the MET complex. We affirm that Pz protein subunits constitute MET channels and that functional interactions with components of the MET complex yield current properties resembling hair-cell MET currents. Our results demonstrate Pz is a MET channel component central to interacting with MET complex proteins. Results account for the MET channel pore and complex.

## Introduction

Transducer ion channels are the sensory modalities’ gateway to the nervous system. The activation of transducer channels generates receptor potentials in sensory receptors, which are converted directly into primary sensory neural codes. The mechanoelectrical transducer (MET) channel complex responsible for detecting sound, gravity, and head acceleration in inner ear hair cells (HCs) identity remains an enigma^1, 2^. The MET complex is housed in a hair-cell actin-based stereocilium^3^ located at the tips^4^ of shorter stereocilia connected to the side of longer stereocilia by a tip link^5–7^ that gates the MET channels^8–10^. In the tip-link’s absence, an anomalous HC MET current can be invoked from the cuticular plate membrane^11, 12^. Mechanical deflection towards the tallest stereocilia exerts tension in the tip link, transmitting force directly onto the MET channel to increase the open probability (P_o_), while deflection towards the shortest stereocilia causes slack in the tip link to reduce channel P_o_^13, 14^. Biophysical and pharmacological features of the MET channels include sub-millisecond activation time constants^15, 16^, weak cation-selectivity, and large molecule permeability, such as the fluorescent dye FM1–43^17–19^, and aminoglycoside antibiotic^20^. Accordingly, the MET channel has a sizeable unitary conductance of ~100-pS^21^.

Considerable evidence has been used to identify components of the tip link and candidate protein subunits constituting the MET complex, but the molecular composition remains unclear. Experiments have shown that the tip link comprises cadherin 23 (Cdh23) at the upper end and protocadherin15 (Pcdh15) at the lower end of the filamentous structure, whereby the two proteins form cis-homodimers that interact in trans *via* their opposing N-termini^22–24^. The transmembrane inner ear protein (Tmie)^25^ and the Ca^2+^/integrin binding family member 2 (Cib2) protein^26, 27^ may be coupled to the MET channel, specifically to the tip link and the cytoskeleton. The Lipoma HMGIC fusion partner-like 5 protein (Lhfpl5)^28^ likely serves as an allosteric modulator of the MET channel. The prevalent promising MET channel candidates have been the transmembrane channel like 1 and 2 (Tmc1) and (Tmc2)^29, 30^. Akin to other MET complexes, Tmc1 and Tmc2 are localized at stereocilia tips^31^, and their onset in HC expression coincides with the beginning of the MET current^32^. Multiple mutations of the *Tmc1* allele yield hearing loss, which alters the MET channel conductance and Ca^2+^ permeability^33^. However, the association between genetic mutations and hearing loss is not unique to the *Tmc* alleles among candidate MET complex^34, 35^. Tmc has structural similarities to the Ca^2+^-activated Cl^−^ channel (TMEM16A)^29^, and Tmc orthologs may constitute a channel^36 37^ that is mechanically sensitive in liposomes^38^. Other attempts have failed to demonstrate the Tmcs as ion channels^39–41^.

Piezo1 (Pz1) and Piezo2 (Pz2) are mechanically-sensitive ion channels^42, 43^ that respond to shear stress and pressure changes in blood vessels and the bladder^44^ and are touch-sensitive in Merkel cells^45^. Pz channels are trimeric assemblies in which each modular subunit consists of a bowl-shaped monomer with a central pore, an extracellular cap, and three curved intracellular beams, generating a lipid curvature^46–48^. Previous reports did not detect Pz1 in the stereocilia of HC^49^, but Pz2 functions may account for the anomalous reversed polarity current (RPI)^12^ — null deletion of *Pz2* aborts high-frequency hearing in mice^50^.

Here we show that HCs express *Pz1* and *Pz2* transcripts and proteins, and the two proteins co-localize at the tips and sides of stereocilia and cuticular plate membranes with and without Tmc1 and Tmc2 co-assembly. Therefore, despite reports regarding Pz1, our results demonstrate a role for both Pz1 and Pz2 in auditory and vestibular HCs. Although sequence homology between Pz1 and Pz2 is ~42%^51, 52^, trimeric interface analyses suggest ~84% homology providing the possibility for hetero-trimeric interaction. We generated non-functional *Pz1* and *Pz2* knockin (*ki*) mouse models, floxed at the ROSA26 locus. Results demonstrate mutant (*ki, MU*) *Pz1* and *Pz2*, but not wildtype *Pz1* under two different HC-specific Cre-recombinase, Myosin 15 (*Myo15 (mc)*) and calretinin (*Calb2 (cc)*), cause hearing and vestibular dysfunction. Co-immunoprecipitation analyses using cochlear tissue and cell lines expressing Tmc- and Pz-fusion proteins revealed the co-assembly of Pz and Tmc and functional coupling, using fluorescence resonance energy transfer (FRET) *in situ* and *in vitro*. Pz is central to the MET complex and interacts with multiple proteins, including Pcdh15, Tmc1, Lhfpl5, Tmie, and Cib2, signifying a chief role in the MET complex assembly. We propose the MET complex comprises multimers of the Pz channel subunits serving as the centerpiece, while other regulatory and indispensable binding partners confer a functional MET unit.

## Results

### Pz1/2 and Tmc1/2 are expressed in inner and outer hair cells (IHCs and OHCs) of cochlear and vestibular organ HCs.

**Figure 1.**
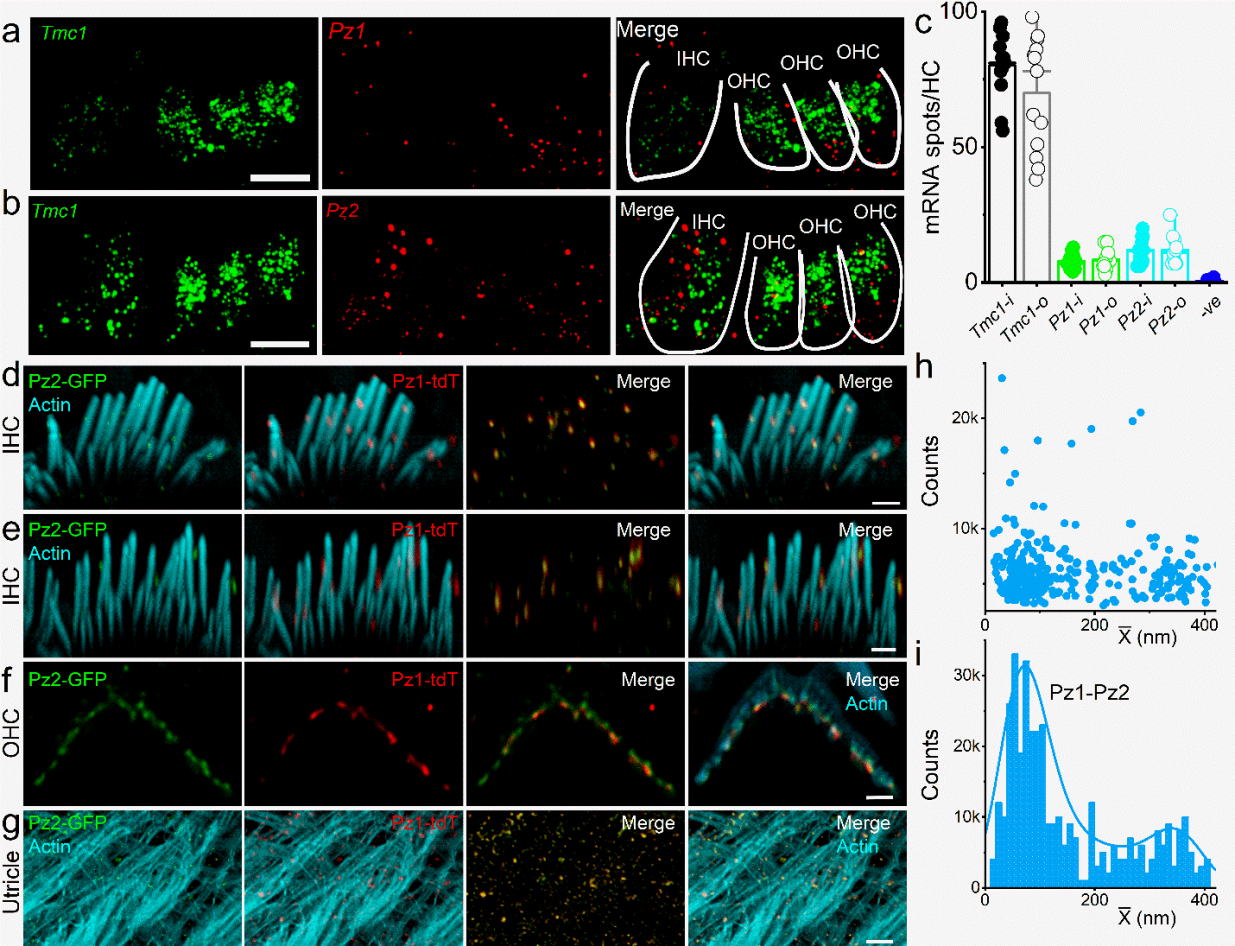
smFISH localizes transcripts encoding *Tmc1*, *Pz1,* and *Pz2* in the IHCs and OHCs. The expression of *Pz* and *Tmc*-encoding transcripts in hair cells (HCs), using smFISH on the isolated organ of Corti (OC) sections from P12 mice. **a-b,** RNA molecules encoding Tmc1 (*Tmc1*) (green), Pz1 (*Pz1*), *and* Pz2 (*Pz2*) (red) were detected as fluorescent puncta in HCs, shown individually and as merged photomicrographs. Outlines of a single row of IHCs and three OHCs are marked in white. Negative and positive control results are shown in **Supplement Fig. 1 (S1)**. **c,** The mean number of RNA molecules detected per HC was calculated as described^85^. *Tmc1* was most abundantly expressed (mean, IHC, noted in the graph as i, 80±3 (n=13), OHC, noted as o, 70±6 (n=13). *Pz1* and *Pz2* expression in IHC (i) and OHC(o) were as follows; (*Pz1i*, 8±1 (n=11), *Pz1o*, 9±1 (n=11), *Pz2i*, 12±2 (n=11), *Pz2o*, 12±2 (n=11), and negative probe showed little to no expression, 0.4±0.3 (n=13) (**Supplement Fig. 1, S1**). Data are plotted to show individual replicates (animals) and mean ± SEM. There were significant differences at the *p*<0.05 level for tested probes (*Tmc1, Pz1,* and *Pz2*) and negative controls F(6,74)=(128) *p* = *5.0X10*^−*37*^. *Post hoc* comparisons using the Tukey HSD test indicate that *Pz1o* vs *Tmc1i* (*p*=*1X10*^−*22*^); *Pz1i* vs *Tmc1o* (*p*=*1X10*^−*25*^); *Pz1o* vs *Tmc1i* (*p*=*1X10*^−*23*^); *Pz1o* vs *Tmc1o* (*p*=*2.1X10*^−*24*^); *Pz2i* vs *Tmc1i* (*p*=3*.4X10*^−*26*^); *Pz2i* vs *Tmc1o* (*p*=*4.4X10*^−*21*^); *Pz2o* vs *Tmc1i* (*p*=*6.1X10*^−*23*^); *Pz2o* vs *Tmc1o* (*p*=*1.8X10*^−*24*^) are significantly different. All experiments were repeated in multiple animal samples noted as **n**, and at least 15 images were collected, sampled blindly by three individuals, and averaged for each independent experiment. Scale bar, 10 μm (**a-b**). **d-e,** GFP and tdT fluorescence was visualized in the whole-mount cochlea from a cross between *Pz1*^*tdT*^ and *Pz2*^*GFP*^ (*Pz1*^*tdT*^*/Pz2*^*GFP*^) in P10 mice IHC and OHCs. The signal of tdT and GFP were enhanced with antibodies to the fluorophores. Stereocilia were counterstained with phalloidin (in blue). Expression of Pz1 and Pz2 are co-localized in IHC (**d-e**) and OHC stereocilia (**f**) and utricular hair bundles (**g**). Regions of solitary expression of the Pz1 and Pz2 were also observed. Scale bar (**d-f**, 1 μm, **g**, 2 μm). **h-i,** Scattered plot and histogram depicting the counts and frequency plots of NND (Pz1-Pz2) in hair cell stereocilia. The bimodal distribution of NND suggests a non-random functional or spatial relationship between the two channel isoforms.

To quantify the relative abundance of candidate MET channel transcripts in HCs, we used single-molecule fluorescent hybridization (smFISH) to determine the expression of *Tmc1*, *Pz1,* and *Pz2* transcripts in postnatal (P) day 10–12 (P10-P12) cochlear sections. Because the present experiments were restricted to hearing onset, and *Tmc2* transcript levels are undetectable after P8^53^, *Tmc2* mRNA was not evaluated. The total number of RNA molecules detected per inner and outer hair cell (IHC/OHC) was calculated across independent replicates (Figs. 1a–c). *Tmc1* (in green, encoding Tmc1) was richly expressed in IHC, and OHC, followed by *Pz2* and *Pz1* (in red, encoding Pz2 and Pz1). Negative and positive controls are shown (Supplement Fig. 1 (S1)). Compared with *Pz1 and Pz2*, *Tmc1* transcript levels are ~7-fold greater than *Pz* in HCs (Fig. 1c).

To validate the Pz1/2 expression in HCs, we used *Pz1-tdT:Pz2-GFP* mice, expressing Pz1 and Pz2 fusion proteins with tdTomato (tdT) and green fluorescence proteins (GFP), respectively^45, 54^. We identified tdT-Pz1 (in red) and GFP-Pz2 (in green), co-localized in IHC and OHC stereocilia tips and sides of the whole-mount cochlear epithelium (Figs. 1d–f, counterstained with phalloidin for actin in blue). Co-localized tdT-Pz1- and GFP-Pz2-puncta were observed in HC stereocilia in whole-mount utricle (Fig 1g). The inter-channel (Pz1-Pz2 and Pz2-Pz1) nearest neighbor distances (NND) distributions are summarized in Figs. 1h–i. There was no statistical difference between NND (Pz1-Pz2) and NND (Pz2-Pz1) in IHC and OHCs. The mean NND (Pz1-Pz2) was 0.47±0.46 μm. A bimodal distribution, with first and second modes at 0.07 μm and 0.41 μm, was calculated, and obtained from a finite mixture of two Gamma functions, suggesting a relationship (either functional or spatial) between the two channels.

The Pz channel consists of trimeric subunits^46^. Pz1 and Pz2 sequences showed ~42% homology^51, 52^. In contrast to the full-length sequence, the identified Pz1 subunit interface revealed 75% identical and 84% conserved residues with Pz2 (S2-3), similar to the heteromeric SK-channel subunit interacting interface that occurs *in vitro* and *in vivo*^55^, suggesting the possibility for hetero-trimeric interaction between Pz1 and Pz2 (see Method for detailed analyses). Results from proximity ligation assays (PLA) also affirmed at least ~40-nm closeness between Pz1 and Pz2 in HCs (S4). The spatial propinquity of the two channel subunits in HC stereocilia and cuticular plate suggested a functional interaction.

**Figure 2:**
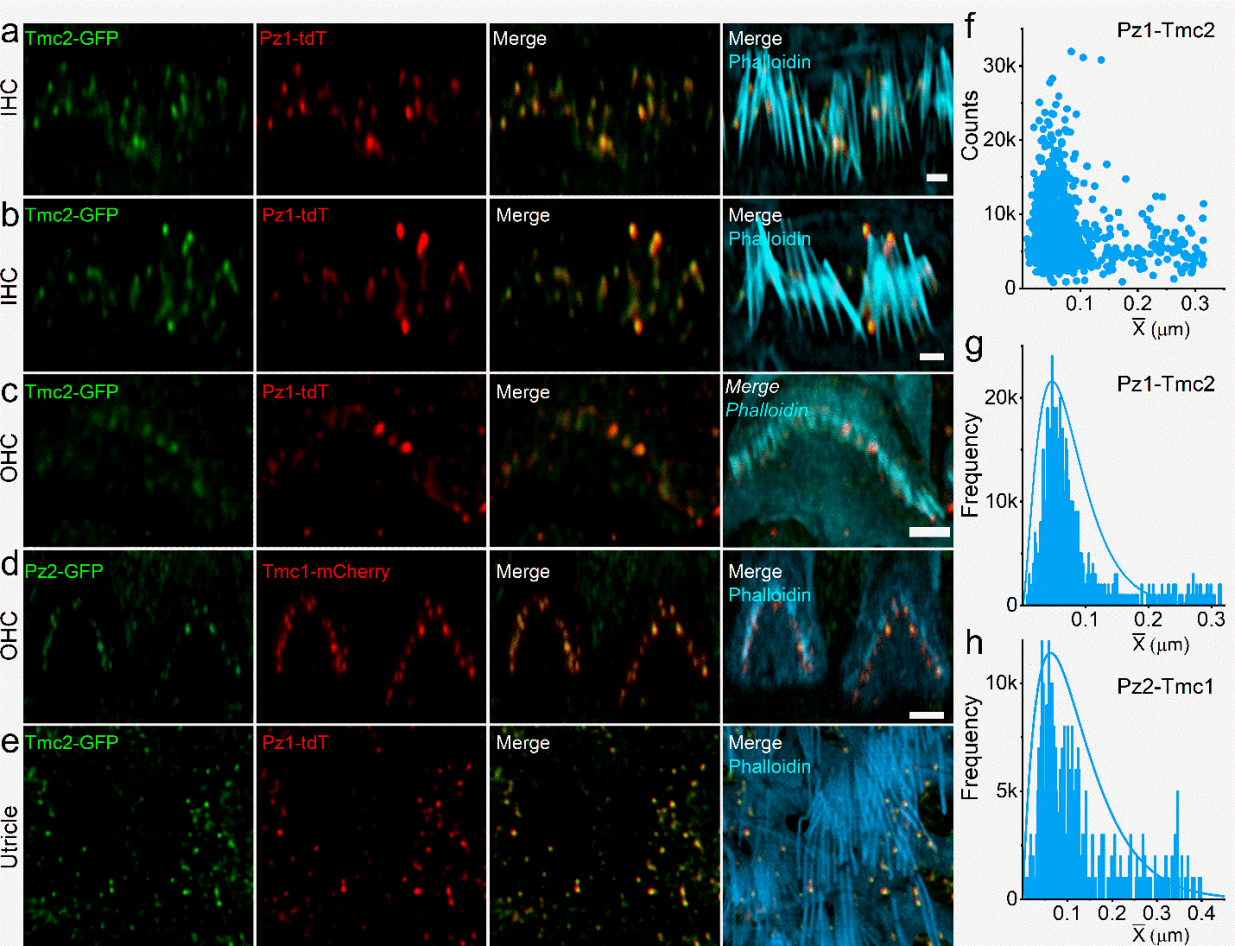
Tmc1, Tmc2, Pz1, and Pz2 are localized in IHC and OHC stereocilia and utricular HC stereocilia bundles. a-c, Confocal fluorescence images obtained from *Tmc2-AcGFP*/*Pz1-tdT* transgenic mice at P10. Tmc2 (green) and Pz1 (red) labeling were strongest at stereocilia tips, and plasma membranes of cuticular plates counterstained in blue with Alexa-405-phalloidin for actin. As indicated, the Tmc2 and Pz2 expression patterns were similar in IHCs and OHCs. (Scale bar, 1 μm). d, Expression of Tmc1 (red) and Pz2 (green) captured from an OHC of *Tmc1-mCherry:Pz2-GFP* mice. For the image shown, Pz2 labeling is seen strongly at OHC stereocilia tips and cuticular plate membrane, while Tmc1 is detected mainly at stereocilia tips. (Scale bar, 1 μm). e, Confocal fluorescence image of utricular HC stereocilia bundles from *Tmc2-AcGFP*/*Pz1-tdT* transgenic mice at P10. GFP-Tmc2 (green) and tdT-Pz1 (red) localize predominantly to the tips of stereocilia, which were counterstained in blue with Alexa 405-phalloidin for actin. (Scale bar, 2 μm). All experiments were repeated in five animal samples, and at least 15 images were collected and sampled blindly by three individuals for each independent experiment. f-h, Scattered plot and histogram depicting the counts and frequency plots of NND (Pz1-Tmc2) and (Pz2-Tmc1) in hair cell stereocilia.

We reasoned that if HC-Pz subunits constitute components of the MET complex, they should localize with Tmc subunits, components of the MET complex^30^. We confirmed this prediction using *Tmc2-AcGFP:Pz1-tdT* and *Tmc1-mCherry*:*Pz2-AcGFP* mice^31^. At P10-P12, *Ac*GFP-Tmc2 and tdT-Pz1 expression was detected in IHCs and OHCs and vestibular HC stereocilia (Figs. 2a–c, e). Co-localized and single-expression of Tmc2 and Pz1 was also detected at the level of the cuticular plate membrane. Fig. 2d shows AcGFP-Pz2 (green) and mCherry-Tmc1 (red) in OHC and IHC stereocilia tips. Serial z-sections revealed co-localization of Tmc1 and Pz2 at stereocilia tips. The Pz1-Tmc2 and Pz2-Tmc1 NND distribution was ~0.4±0.5 μm in IHC stereocilia and ~0.5±0.4 μm in OHC stereocilia (Figs. 2f–h). At the cuticular plate, segregation was apparent between the two proteins (S5). While these results showed Pz was located at the presumed MET-current generation site, we needed to test whether the response properties of the Pz-current resemble the HC MET currents.

### Properties of the Pz-channel current resemble the HC MET current.

We overexpressed mouse *Pz1* (*mPz1*) plasmid in Neuro2A (N2A) cells. Membrane localization revealed by TIRF imaging accounted for ~13% of mPz1-mClover3 fusion protein expression. By contrast, ~8% of mTmc1-mRuby3 fusion protein membrane expression was measured 24-hrs post-transfection (S6). After 24–48 hours of mPz1 plasmid transfection and mechanically displacing the N2A cell body membrane via a piezo-electric-driven probe, whole-cell recordings at −80 mV revealed a Pz1 current (I_Pz1_). The maximum I_Pz1_ amplitude ranged from 0.7 to 6 nA. The mean maximum I_Pz1_ was 3±1 nA (n = 17; (S7a)). In contrast, endogenous mechanically-activated (MA) current in N2A cells ranged from 0.03–0.2 nA with a mean value of 0.10±0.04 nA (n=44) in keeping with earlier report^42^. The current (I_Pz1_)-displacement relationship was fitted with a two-state Boltzmann function with half-activation (X_1/2_) = 0.8±0.1 μm and displacement sensitivity X_1/2_ at = 61.1±6.2 pA μm^−1^ (n = 17; S7b). The I_Pz1_ was activated with a constant displacement and stepped to varying voltages. The resulting I_Pz1_ traces are shown as an inset (S7c). The instantaneous current-voltage relation yielded a linear function between −120 and +90 mV. The reversal potential was −7±5 mV (n = 14) (S7c), consistent with a nonselective cation channel, and compatible with the HC MET current^43, 56^.

Hair cell MET current displays a rectification mechanism, suggesting a reduced pore size at positive voltages^57^. We used small-amine molecules as permeant ions^58^ to examine the channel-pore shape by plotting the current-voltage relation of the relative I_Pz1_ at −90 and −120 mV (S7d-e). The channel inner and outer radii were estimated, using reported empirical fitting analyses, by plotting the amine radius vs. the relative current at −120 mV^58, 59^. An inner-face pore radius of 7±4 Å and an outer-face radius of 20±5 212B was estimated (S7f-g). The findings align with HC MET channel^53^ estimations and the upward concave structure of Pz^48^. Next, the Ca^2+^-dependent I_Pz1_ decay was evaluated with varying concentrations of patch-pipette Ca^2+^ chelators, EGTA and BAPTA. The results reflect the Ca^2+^ dependence of HC MET current adaptation^60, 61^ (S8a-e). At −80 mV, where the driving force for Ca^2+^ is greater, with 5-mM pipette EGTA, I_Pz1_ decay was rapid (decay time constant (τ_decay_) at maximum I_Pz1_ = 24±3 ms (n=15)). In contrast, at 70 mV, the τ_decay_ of I_Pz1_ recorded with patch-pipette-BAPTA, a more efficient Ca^2+^ buffer (I_Pz1_ τ_decay_ at −80 mV = 38±4 ms (n=5) and 70 mV = 97±10 ms (n=6)), demonstrated the Ca^2+^-dependence of I_Pz1_ decay. However, the displacement-response relation remained significantly unaltered using different pipette Ca^2+^ buffers (S8). The findings affirmed the Ca^2+^ dependence of I_Pz1_ decay kinetics, which parallels the HC MET current kinetics.

We co-expressed mouse Pz1 and Pz2 channels and Tmc isoforms as proxies to test the functional significance of co-localization in HC stereocilia (Fig. 2, S4). Expression of mPz1 and mPz2 by themselves yielded distinct current profiles. Pz2 currents (I_Pz2_) showed fast activation and decay kinetics (S9a). The τ_decay_ for maximum I_Pz2_ and I_Pz1_ was <10 and >10 ms, respectively. The current-density, after 36–40 hours post-transfection for I_Pz2_ was ~2.5-fold (323±78 pA/pF, n = 17) greater than I_Pz1_ (129±25 pA/pF, n = 19). Co-expression of *mPz1* and *mPz2* in an equivalent molar ratio (1:1) produced current (I_Pz1/2_) with properties resembling a hybrid of the two-channel currents with τ_decay_ ranging from 9–24 ms (mean τ_decay_ = 17.4±4.7 ms, n=14). The normalized displacement-current response relationships were fitted with a two-state Boltzmann function, and half-maximum displacements (X_1/2_, in μm) were estimated for I_Pz1_ = 0.87±0.01 (n = 11), I_Pz2_ = 0.67±0.01 (n = 10) and I_Pz1/2_ = 0.76±0.01 (n = 12) (S9b). There were notable differences in MET current densities and τ_adapt_ after co-expressing mPz1 with mTmc1 or mTmc2 (S9c-e).

The mechanically-activated current decay for mPz1 and mTmc2 transfected N2A cells increased compared to mPz1 alone, and the τ_decay_ decreased ~4.5-fold, indicating the regulation of mPz1-mediated current by Tmc2. Another visible and consistent effect of singly and co-expressed m*Pz1, Tmc1,* and *Tmc2* was the robust amplitude of m*Pz1*-*Tmc1* current compared to *mPz1-Tmc2* or *mPz1* current alone. For similar transfection conditions, the total I_Pz1/T1_ density (292±40 pA/pF, n=10) was ~2.4-fold greater relative to I_Pz1_ (122±18 pA/pF, n=10), and I_Pz1/T2_ (113±22 pA/pF, n=10). Co-expression of *Tmc1* or *Tmc2* with *mPz1* shifted displacement-response relationships rightward of I_Pz1_ alone, with Tmc2 exerting the most pronounced effect (S9). In contrast, the steepness of the displacement-response curves at X_1/2_ remained relatively unchanged (S9f). Because the present experiments were limited by the relatively slow rise-time of the mechanical stimulator and because of coarse mechanical stimulation, quantitative analyses were not performed of the displacement-response relationship and the time constants of current activation.

### Evidence for the functional coupling of Pz and Tmc using Förster resonance energy transfer (FRET)

Motivated by Pz and Tmc co-localization in HC stereocilia and I_Pz_ property modulations by Tmc1/2 co-transfection (I_Pz/T1/2_), we tested additional evidence for functional coupling between the two proteins. In OHC stereocilia, we examined the co-expression of Pz2-GFP and Tmc1-mCherry in mice that were crossbred from heterozygotes for each construct, whereas in N2A cells in vitro we examine co-transfected Pz1 tagged with mClover3 (mPiezo1-mClover3) and Tmc1 tagged with mRuby3 (mTmc1-mRuby3). Results examined after FRET-fluorophore activation are shown in S10. FRET efficiencies were quantified using acceptor photobleaching (S10). Results from FRET experiments *in vitro* showed FRET efficiency of ~10%. Experiments conducted using OHCs stereocilia expressing Pz2-GFP and Tmc1-mCherry showed similar FRET efficiency (S10), suggesting that the two proteins are located spatially and are functionally coupled.

### I_Pz1/T1_ exhibited similar pharmacological properties to MET current.

MET current block by aminoglycosides is a pharmacologic trademark^20, 56^, consequently, I_Pz_ or current ensuing from a Pz- and Tmc association should be affected to resemble dihydrostreptomycin (DHS) effects on HC MET currents. Thus, we tested for I_Pz1_ sensitivity towards DHS. DHS application on N2A cells expressing mPz1 alone inhibited the I_Pz1_ in a dose-dependent manner. Results show an IC_50_ of 270±135 μM (n = 7), with ~65% of the maximum I_Pz1_ was blocked by 200 μM neomycin (n = 2, S11 a-c). Typically, HC MET current is at least ~20-fold more sensitive towards DHS than I_Pz1_ DHS-sensitivity in N2A cells (S11). Since previous results showed that *Tmc* and its mutations reduced HC MET current sensitivity to DHS^62, 63^, we tested I_Pz1/T1_ block by DHS. Pz1-Tmc1 co-transfection yielded current IC_50_ of ~70±6 nM (n=7) in the presence of DHS (S11). The ~1000-fold increased sensitivity of I_Pz1/T1_ towards DHS provided additional evidence for the Pz-Tmc functional association.

### mPz1 in lipid bilayer exhibited similar single-channel conductance as MET current

HC MET single-channel conductance is ~100 pS^64, 65^. We subjected the Pz1-transfected N2A-cell membrane to pressure at varying voltages, and the results showed a Pz1 unitary conductance of ~36 pS (S12), consistent with previous reports^42^ but at odds with HC MET channel conductance. However, I_Pz_ is modulated by regulatory and interacting proteins, and the force-response properties depend on membrane lipids^66^. Moreover, recent studies suggest that HC Tmc may exhibit lipid scramblase activity^67^. Using purified mPz1 from brain lipid extract in lipid bilayer with defined lipid composition (PE: PS: PC 5:3:2), we demonstrate that the unitary mPz1 current was ~10 pA at −100 mV with single-channel conductance of ~100 pS, in keeping with the reported HC MET single-channel conductance. DHS, GsMTx4, and ruthenium blocked the mPz1-channel activity in lipid bilayers, consistent with HC MET’s current pharmacology (S13).

### Knockdown of Pz by CRISPR/Cas9-mediated genome engineering

Data accrued motivated us to test *Pz* functions *in vivo* in the inner ear, using non-functional *Pz1* and *Pz2* gene knockin and HC-specific Cre-recombinase strategies. While other gene manipulation strategies, such as mutated-gene knockin at the promoter sites could have been adopted, we opted against such a design to mitigate potential embryonic lethality because of the channels’ essential roles in other tissues. Additionally, the amassed data suggested that Pz1 and Pz2 are expressed in HCs and the two may form heterotrimers (S2-3).

**Figure 3.**
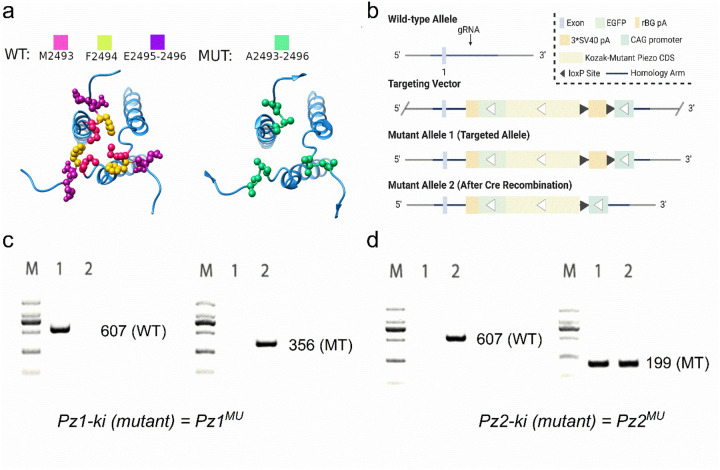
Generation of *knockin (ki)* mouse models. **a.** Introduction of the mutation into the *Pz1* and *Pz2* loci by gene targeting. **a,** Cartoon of a short segment of the C-terminal domain (CTD) of Pz protein showing the sequence residues 2493–2496 in Pz1 MFEE mutated to AAAA. The corresponding conserved sequence in Pz2 was at 2767–2770, mutated to AAAA. The plasma membrane appears as a blue rectangle. Amino acids are labeled as indicated: polar, hydrophobic, acidic, and basic residues. **b,** CRISPR/Cas9-mediated genome engineering was used. The *Pz* conditional overexpression *ki* mouse line features a “CAG promoter-loxP-3*SV40 pA-loxP-Kozak-Mutant *Pz1* CDS-P2A-EGFP-rBG pA” cassette inserted into intron 1 of the ROSA26 locus. The *Pz2* conditional overexpression knockin mouse line features a “CAG promoter-loxP-3*SV40 pAloxP-Kozak-Mutant *Pz2* CDS-P2A-tdTomato-rBG pA” cassette also inserted into intron 1 of ROSA26 locus. **c-d,** Tail genomic DNA was digested for *Pz1* (*Pz1-ki*) genotyping. We developed a PCR assay (35 cycles) with a pair of primers: (607 bp: F:5”-AAGCACGTTTCCGACTTGAGTTG-3” and R:5”-GGGTGAGCATGTCTTTAATCTACC-3”). The primers for the mutant were: (356 bp: F:5”-TTCAGGGTCAGCTTGCCGTAG-3” and R:5”-CTGCCGTGTGTGGACCGCATCCT-3”). The mutant *Pz2* (*Pz2-ki*) primers were: (199 bp: F:5”-ACCTCCTCGCCCTTGCTCACCAT-3” and R:5”TCGTGAGGGAGACAGGGGAGTTG-3”).

We generated two knockin (ki) mice in a C57BL6/NTac genetic background using CRISPR/Cas9-mediated genome engineering (Cyagen Biosciences, Inc., Santa Clara, CA, USA). The non-functional forms of *Pz1 (Pz1*^*MU*^) and *Pz2 (Pz2*^*MU*^) were flanked by loxP sites and inserted into intron 1 of the ROSA26 locus in the two ki mice. Additionally, a control wildtype (WT) *Pz1*^*WT*^ was generated. Both the *Pz1-mutant* and *Pz2-mutant ki* (*denoted as Pz1*^*MU*^*, and Pz2*^*MU*^) coding sequences contain four amino acids in the C-terminal domain (CTD), where the sequence “MFEE” was replaced with amino acids “AAAA” (Fig 3a–b). The sequences correspond to amino acids 2493–96 for *Pz1* and 2767–2770 for *Pz2*. The selection of EE:AA residue substitutions stems from earlier reports^68^ demonstrating that while mutant Pz proteins with substitutions of the AA residues can be targeted to the plasma membrane, they are non-conductive. Similar results were confirmed for the MFEE:AAAA mutants transfected in N2A cells (S14). We performed analyses *in silico* to predict the top five most likely off-target mutations that could occur with the CRISPR approach and ensured the mice did not harbor them. Mice were also backcrossed unto C57 mouse lines for ten generations to mitigate potential off-target effects. Genotyping strategies are described (Fig. 3c–d; see Methods). We denote the mutant ki as *Pz1*^*MU*^ and *Pz2*^*MU*^. A control mouse with wildtype (WT) Pz1 at the ROSA 26 locus was also generated (*Pz1*^*WT*^).

Calretinin is encoded by the *Calb2* gene, subserving as a major Ca^2+^ buffer^69, 70^ in HCs, whereas myosin 15 is encoded by the *Myo15* gene, showing early postnatal expression in HCs^71, 72^. *Calb2* is also expressed in auditory neuron subtypes^73^. We used the two Cre lines as complementary strategies to regulate *Pz* knockin mouse lines. Procedures, generation, genotyping, and validation of *Myo15*- (*mc*) and *Calb2-Cre (cc)* mouse lines are described in the Methods section and Supplement **15 (**S15). We first confirmed HC-specific Cre expression by crossing *mc* and *cc* with the *Ai9-tdT (Ai9(RLC-tdT;* JAX strain #00709) and *Calb2-GFP* mouse lines (S15). Additionally, we inactivated *Pz1* and *Pz2* in HCs individually, using *Pz1*^*MU*^ and *Pz2*^*MU*^ mice^74,45^ crossed with *mc* and *cc* mouse lines to assess the potential differential roles of the two proteins. We will refer to the resulting lines after crosses as *mc-Pz1*^*MU*^ and *cc*-*Pz1*^*MU*^*, mc-Pz2*^*MU*^ and cc-*Pz2*^*MU*^. The controls used were *cc, mc* or *Pz1*^*WT*^, where the wildtype Pz1 channel was knockin at the ROSA 26 locus. The auditory and vestibular traits in the control mice were similar and thus used interchangeably.

Pz conditional knockout mouse models *Pz1-knockout (Pz1*^*ko*^*) cross with the Cre-lines* were assessed as *mc-Pz1*^*ko*^, *mc-Pz2*^*ko*^, *cc-Pz1*^*ko*^, and *cc-Pz2*^*ko*^. The crosses of heterozygous floxed-lines with and without the Cre allele yielded offspring with a 1:2:1 ratio for the wild-type (WT), heterozygous, and homozygous floxed allele, respectively, suggesting no embryonic lethality. *Pz1*^*MU*^ and *Pz2*^*MU*^ mice appeared normal without obvious behavioral defects (rotarod test, data not shown) and body weight issues (S16a). Real-time RT-PCR analysis of wild-type and *Pz1/2*^*MU*^ cochlear samples at P10 demonstrated normal *Pz* transcript levels (data not shown). We performed immunostaining of whole-mount cochlear tissue using the Pz1 antibody and detected correctly targeted Pz1 in HC stereocilia in the *Pz1*^*MU*^ mouse samples as expected (S16b).

### The auditory and vestibular traits of *Pz*^*MU*^ and *Pz*^*ko*^ mice

**Figure 4.**
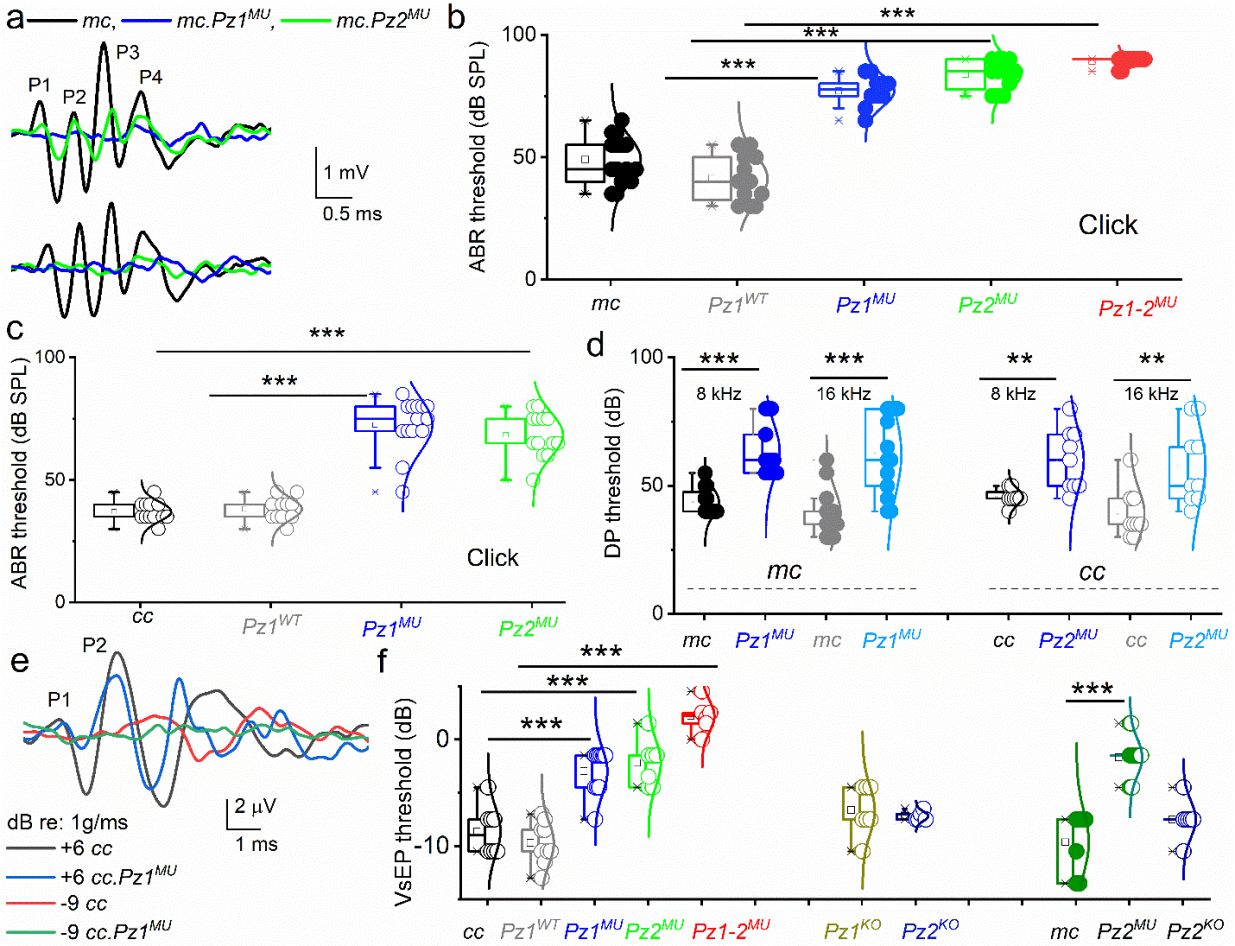
Hearing loss and vestibular hypofunction in *Piezo (Pz) knockin (ki, mutant (MU), Pz*^*MU*^*))* mice. **a,** Typical ABR traces evoked click stimuli at 80 and 60 dB. Mice genotypes were *Myo15*-*Cre* (*mc*), control group, black traces, and *Pz1* (*mc-Pz1*^*MU*^, blue) and *mc-Pz2*^*MU*^ (green) mice (8-weeks-old). The peaks (P) of the waveforms represent neural activity from the primary auditory nerve, cochlear nucleus, superior olivary complex, and higher brain nuclei. **b-c,** Summary of the ABR thresholds to click stimuli. **b.** Comparison of ABR thresholds for mc (●), WT Pz1 knockin at ROSA 26 locus (denote *mc-Pz1*^*WT*^ (indicated *Pz1*^*WT*^), (●)), *Pz1*^*MU*^ (●), *Pz2*^*MU*^ (●) and double *Pz1–2*^*MU*^ (●) in 8-week (w)-old mice. Mice were littermates, and male and female numbers were equal. When odd numbers are reported, the females outnumber males. We combined the data since no significant differences were detected between the sexes. Data are shown as mean ± SD. For click sound threshold (in dB), there were significant differences at the *0.05* level for *Pz1*^*WT*^ comparisons, one-way ANOVA, F(4,56) = (105) *p* = *2.4X10*^−*25*^. *Post hoc* comparisons using the Tukey HSD test at 8-w, *Pz1*^*WT*^ (41±10, n=12), *Pz1*^*MU*^ (77±6, n=12) (*p*=*3.3X10*^−*6*^); *Pz2*^*MU*^ (84±6, n=12) (*p*=*1.4X10*^−*6*^); *Pz1–2*^*MU*^ (89±2, n=12) (*p*=*1.9X10*^−*8*^) are significantly different. There were no significant differences between *mc* vs. *Pz1*^*WT*^ (p = 0.99). **c**, The click ABR thresholds for control (*calb2-cre (cc(*○))) compared with control *cc-Pz1*^*WT*^ (denoted *Pz1*^*WT*^(○)), *Pz1*^*MU*^ (○) and *Pz2*^*MU*^ (○) at 8-w of age. Littermate mice were tested. There were significant differences at the *0.05* level for *cc* comparisons, one-way ANOVA, F(3,46) = (72) *p* = *2.1X10*^−*17*^. *cc* (37±4, n=12), *Pz1*^*WT*^ (38±5, n=11) (*p* = *0.98*), *Pz1*^*MU*^ (73±11, n=14) (*p*=*1,5X10*^−*9*^); *Pz2*^*MU*^ (69±9, n=13) (*p*=*1.9X10*^−*10*^). There were no significant differences between *cc* vs. *Pz1*^*WT*^. **d,** OHC functions were evaluated by measuring DPOAE thresholds at 8 and 16 kHz in 8-w-old *mc, cc,* and corresponding *Pz1*^*MU*^ mice. Data are shown in (mean ± SD). For *mc* mice at 8 kHz, threshold (dB) *mc* (44±5, n=12 (●)) vs. *Pz1*^*MU*^ (64±10, n=13(●)) (*p*=*8.8X10*^−*6*^); 16 kHz, (39±9, n=13(●)) vs. (62±16, n=13(●)) (*p*=*1.9X10*^−*4*^). For *cc* mice at 8 kHz, threshold (dB) *cc* (47±9, n=9 (○)) vs. *Pz1*^*MU*^ (60±12, n=9(○)) (*p*=*7.0X10*^−*3*^); 16 kHz, (39±10, n=9(○)) vs. (58±15, n=9(○)) (*p*=*7.4X10*^−*3*^). **e-f**, Reduced VsEP in *Pz-ki* but not *Pz-ko* mouse lines. **e,** Typical VsEP intensities (−9 and +6 dB) for control, *cc,* and *Pz1*^*MU*^ mice at 8-w. Response peaks are indicated for P1 and P2. Stimulus intensities are in dB re: 1.0 g/ms. Response peak amplitude decrease as stimulus amplitude decrease, and no response ensues below threshold values. **f,** Summary data, contrasting VsEP thresholds of the indicated mice genotypes. Data were obtained from 8-w-old mice. For *cc* (○) vs. *Pz1*^*WT*^(○)*, Pz1*^*MU*^(○), *Pz2*^*MU*^ (○), and *Pz1–2*^*MU*^ (○) (note that the double mutant was on mc and not on cc) mean threshold values (in dB re: 1g/ms) and comparison are shown for *cc* (mean (n))= −8.6±2.2(8) vs. *Pz1*^*WT*^ −9.7±2.0 (9), *p*=*3X10*^−*1*^; *Pz1*^*MU*^ −3.0±2.1 (10), *p*=*7.3X10*^−*5*^*; Pz2*^*MU*^ −2.2±2.1 (7), *p*=*7.9X10*^−*5*^; and *Pz1–2*^*MU*^ 2.2±1.5 (6), *p*=*1.6X10*^−*7*^. Comparing cc with *Pz1 and Pz2* knockout (ko) (*Pz1*^*ko*^, *Pz2*^*ko*^) mice; *Pz1*^*ko*^ −6.6±2.3 (7), *p*=*1X10*^−*1*^; *Pz2*^*ko*^ −*7.2*±0.4 (5), *p*=*1.2X10*^−*1*^. Comparing mc (●) with *Pz2*^*MU*^ (○*) and Pz2*^*ko*^ (○) mice; mc −9.6±2.9 (7), vs *Pz2*^*MU*^ −1.7±1.7 (16), *p*=*1.3X10*^−*4*^; *Pz2*^*ko*^ −*7.5*±1.7 (7), *p*=*1.2X10*^−*1*^.

We analyzed auditory brainstem responses (ABR) to broadband click- and pure tones of 4, 8, 16, and 32 kHz stimuli at various sound-pressure levels (SPL). ABRs were measured at 4- and 8-weeks of age. Representative ABR traces show reduced characteristic waveform peaks and increased latencies using click sounds at 60- and 80-dB SPL (Fig. 4a). From 4 to 8 weeks, *mc-Pz1*^*MU*^ mice exhibited an ~20 to 35 dB threshold increase, while *mc-Pz2*^*MU*^ mice showed an ~40 dB threshold shift compared to age-matched control *mc* and *mc-Pz1*^*WT*^ mice for broadband click (Fig. 4b) and across all tone pip stimuli (S17). Results indicated hearing dysfunction by progressively elevated ABR thresholds, with severe hearing loss greater than 70–80 dB SPL thresholds for *mc-Pz1*^*MU*^ and *mc-Pz2*^*MU*^ and profound hearing loss by 12 weeks (threshold at or above 90 dB for click sound) and concomitant threshold elevations across all tone stimuli. However, the elevated thresholds were more significant at 32 kHz than at 4–16 kHz in 4-week-old mice, and a progressive increase in ABR thresholds was observed at 8 weeks in the *mu* relative to control mice (S17). Almost complete hearing loss (threshold ≥ 90 dB) was recorded for the double mutant *mc-Pz1–2*^*MU*^ mice by 8 weeks of age (Fig. 4b). ABR thresholds for the *cc-Pz1*^*MU*^ and *cc-Pz2*^*MU*^ were similar to *mc-Pz1*^*MU*^ and *mc-Pz2*^*MU*^ mice (Fig. 4 S17). In contrast, null deletion of individual *Pz1* or *Pz2* alone produced modest ABR threshold elevations, using *Myo15 and Calb2 Cre* lines and click sound as documented (Fig. 4). Attempts to generate knockouts of both isoforms, thus far, have failed. We suspect embryonic lethality despite using multiple Cre lines.

Increased ABR thresholds may suffice to capture IHC malfunction, whereas distortion product otoacoustic emissions (DPOAEs) are acoustic measurements of OHC activity. DPOAE thresholds for the *mc-Pz1*^*MU*^ and *mc-Pz2*^*MU*^ mice were elevated relative to age-matched control mice (Fig. 4d). Similar ABR and DPOAE results were obtained in the *cc-Pz1*^*MU*^ and cc-*Pz2*^*MU*^ mouse lines. Results suggest compromised integrity of electromechanical transduction and a decline in the competence of IHC and OHC functions.

Pz channels’ functional roles *in vivo* in vestibular end-organ HCs were examined by measuring compound action potentials, the vestibular sensory evoked potential (VsEP)^75^. VsEP originates from HCs, the vestibular nerve, and its central relay activity in response to linear acceleration pulses. Figure 4e shows VsEP waveforms for *cc* control and *cc-Pz1/2*^*MU*^ *and mc-Pz1–2*^*MU*^ *double* mice. The summary data show that VsEP thresholds in single *Pz1* or *Pz2* mutant knockins are significantly elevated relative to those for the age-matched controls (Fig. 4f). Compared to the double mutant mice (*Pz1–2*^*MU*^), relative to controls, the VsEP threshold differences were even more remarkable. However, results from a null deletion of individual *Pz1* and *Pz2* show no significant effects (Fig. 4f).

### Mechanotransduction is attenuated in *Pz*^*MU*^ mice.

We monitored the extent-of-altered HC transducer currents in the *Pz*^*MU*^ mice to explain the observed hearing phenotype. The rapid uptake of the lipophilic dye FM1–43 occurs through the MET channels^17, 19^. At rest, HC MET channels’ P_o_ is ~0.1, so we reasoned that a nonconducting Pz subunit would further reduce the resting P_o_ and inhibit HC FM1–43 dye uptake. We examined FM1–43 dye loading in *mc-Pz1*^*MU*^ and *mc-Pz2*^*MU*^ HCs from the apical-to-middle cochlear region, representing characteristic frequencies (CFs) of 4 to 10 kHz from P12 mouse cochleae and compared them to aged-matched *mc* controls. Local perfusion of FM1–43 dye resulted in intense labeling first at the hair bundle level 1 (L1), followed by dye membrane-partitioning and diffusion across the cuticular plate (L2) and basolateral (L3) aspects of HCs. On average, chronological images from the three levels show intense labeling after 3–10 sec of 10 μM FM1–43 exposure in the *mc* control and faint labeling in *mc-Pz1*^*MU*^ HCs (S18). Z-stack images were taken at the cuticular plate level in 5-sec intervals post-dye exposure (S18). The time constants (τ) of dye loading at L2 were: 23±3 sec (n=5) for controls; 61±6 sec (n=4) for *Pz1-ki*^*m-c*^; and 58±8 sec (n=4) for *Pz2-ki*^*m-c*^. For clarity, only L2 data are plotted (S18). We conclude that the expression of a non-functional Pz subunit suffices to reduce but not altogether abolish FM1–43 dye loading in IHCs and OHCs.

**Figure 5.**
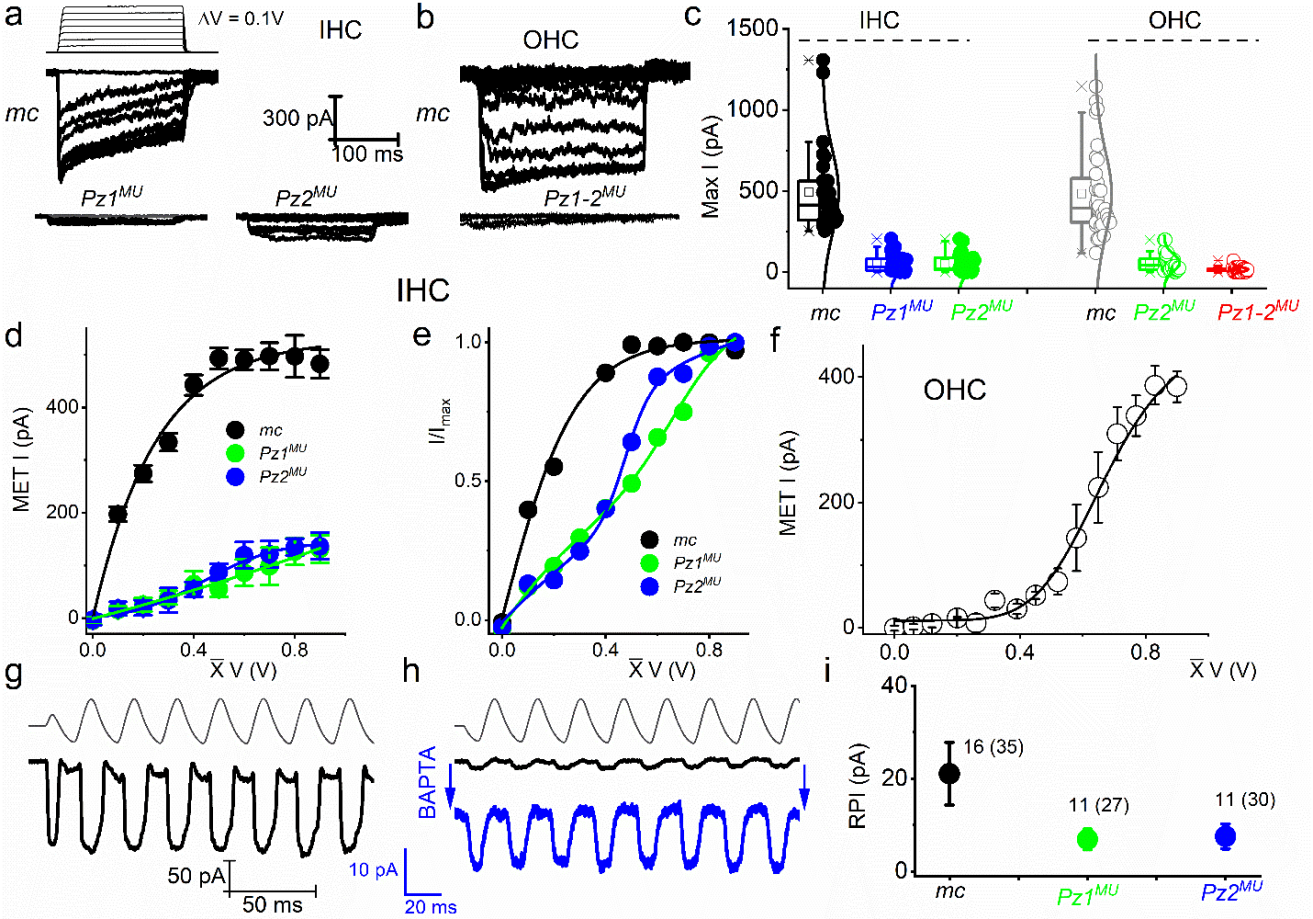
Evaluation of MET channel functions using FM1–43 permeation and MET currents in IHC and OHC stereocilia bundles. **a,** Typical MET current traces in IHCs from control (*mc*), *Pz1*^*MU*^, and *Pz2*^*MU*^ mice at P10 in response to a series of ~200-ms hair bundle displacements, using fluid-jet deflection towards the taller stereocilia. Hair cells were held at −80 mV. All recordings were made from apical IHCs. Bundle deflection was elicited with 0.1–0.9 V pressure clamps in 0.1-V steps. The exact bundle displacement estimation was not determined. For clarity, a few traces were omitted. Current traces from *Pz1*^*MU*^ and *Pz1*^*MU*^ cochlear IHCs are shown. **b,** Current traces recorded from an OHC from mc mouse cochlea. Below are the traces from *Pz1* and *Pz2* double mutant knockin (*mc-Pz1–2*^*MU*^) OHC. **c,** Analyses of group data of the maximum IHC and OHC MET current measured (mean ± SD) from control, *mc*, 493±255 pA (n = 30) from experiments seven control, nine *mc-Pz1*^*MU*^, 53±53 pA (n=30 IHCs) and 13 *mc-Pz2*^*MU*^, 52±58 pA (n = 30 IHCs) mice. One-way ANOVA, F(2 87) = 82, *p* = *1.1X10*^−*20*^ *mc* vs. *mc-Pz1*^*MU*^, *p*=*1X10–25*; *mc* vs. *mc-Pz2*^*MU*^*, p*=*1X10–23*; mc-*Pz1*^*MU*^ vs mc-*Pz2*^*MU*^, *p*=*1.0*. For OHCs *mc,* 484±263 pA (n=32) from eleven control, eight *mc-Pz2*^*MU*,^ 58±52 pA (n=32), and six *mc-Pz1–2*^*MU*^, 15±15 (n=29). One-way ANOVA, F(2 90) = 85, *p* = *2.2X10*^−*21*^ *mc* vs. *mc-Pz2*^*MU*^, *p*=*1X10*^−*26*^; *mc* vs. *mc-Pz1–2*^*MU*^*, p*=*1X10*^−*35*^; mc-*Pz2*^*MU*^ vs mc-*Pz1–2*^*MU*^, *p*=*0.6*. **d,** Summarized displacement (in pressure-clamp voltage (V))-current-response relationship for control, *mc (black)*, vs. *mc-Pz1*^*MU*^ (green) and *mc-Pz2*^*MU*^ (blue). **e,** Normalized displacement response relationships fitted with a two-state Boltzmann function for control (black) and a three-state Boltzmann function for *mc-Pz1*^*MU*^ (green) and *mc-Pz2*^*MU*^ (blue). **f,** Normalized displacement response relationships fitted with a two-state Boltzmann function for OHC MET current (black) **g-i,** Representative MET currents were elicited with a 40-Hz sinusoidal deflection of P10 apical IHC stereocilia bundles in a control mouse (*mc*) with and without BAPTA treatment. IHCs were held at −80 mV in all recordings. The fluid jet’s driving voltage (~0.5-V) is shown. Positive driving voltage represents bundle deflection toward the taller stereocilia. It yielded inwardly directed MET current. After BAPTA (5-mM) treatment, the residual MET current had reserve polarity (RP) such that the inwardly directed MET current’s peak coincided with the sinusoidal deflection’s trough. BAPTA treatment reduced the MET current by ~10-fold. For *mc-Pz1*^*MU*^ and *mc-Pz2*^*MU*^ IHCs with measurable MET currents, the post-BAPTA-residual MET currents had reversed polarity. In most *Pz*^*MU*^ IHCs, the polarity of BAPTA-residual MET current was challenging to measure because of signal-to-noise levels. **i,** Summary data for BAPTA-mediated residual MET currents (RPI) in IHCs in *mc-Pz1*^*MU*^ and mc-*Pz2*^*MU*^ in P10 mice. The numbers of IHCs recorded are indicated in parenthesis, and the ones with measurable RPI are indicated without parenthesis. Data were obtained from at least 4 different mice in each group. Values in I are mean ± SD, control 21±3 pA (n = 16); *mc-Pz1*^*MU*^ 7±1 pA (n = 11); *mc-Pz2*^*MU*^ 8±1 pA (n = 11). One-way ANOVA F(2 35) 12, *p* = *1.4X10*^−*4*^; control vs. *mc-Pz1*^*MU*^, *p* = *6.3X10*^−*4*^; control vs. *mc-Pz2*^*MU*^, *p* = *0.001*; *mc-Pz1*^*MU*^ vs. *mc-Pz2*^*MU*^, *p* = *0.98*.

To examine whether the reduced FM1–43 loading in *mc-Pz*^*MU*^ mouse HCs matches with transducer currents and to obtain direct evidence for Pz’s role in HC MET currents, we recorded IHC and OHC MET currents in the whole-cell configuration from P10-P12 cochleae (Fig. 5a–b). HCs were held at −80 mV. IHC and OHC MET current in control mice showed varied activation and adaptation kinetics, as shown previously^49, 76, 77^ (Fig. 5a–b). The size of the maximum MET current was significantly smaller in *mc-Pz1*^*MU*^ and almost negligible in the *mc-Pz1–2*^*MU*^ compared to control HCs (*mc*) (Fig. 5c). The current-displacement plots showed a notable difference in MET currents from *mc* compared to *mc-Pz1*^*MU*^ and *mc-Pz2*^*MU*^ mice. While the normalized current-displacement curve for control HCs was well-fitted with a two-state Boltzmann function, the relationships derived from the *mc-Pz1*^*MU*^ and *mc-Pz2*^*MU*^ IHCs were best matched with a three-state Boltzmann function (Fig. 5d). Similar results we obtained for OHCs MET currents (Fig. 5c, f). We examined the RPI following the post-BAPTA application to disrupt the tip links. Figure 5g shows the MET current generated using sinusoidal mechanical stimuli. In Figs. 5h–I we illustrate the remaining current after a 5-mM BAPTA solution bath application. The expanded trace, in blue, shows the features of the RPI. At P12, the maximum RPI (in pA) from IHCs was significantly smaller in mc-*Pz1*^*MU*^ compared to *mc* control mice (Fig. 5i).

### OHC electromotility in *Pz*^*MU*^ mutant and control mice was relatively intact

DPOAE measurements, which represent the coarse assessment of cochlea-sound amplification, suggest mild OHC dysfunction in the *Pz1*^*MU*^ and *Pz2*^*MU*^ mouse models. We determined the roles of the Pz channels in OHCs by assessing OHC electromotility. Electromotility describes OHC functions expressed visibly as length changes in the OHC cell-body, mediated by a voltage-dependent gating-charge movement and measured as nonlinear capacitance (NLC) changes^78, 79^. OHC NLC at P18-P21 in control and *Pz1*^*MU*^ and *Pz2*^*MU*^ mice showed substantial differences between the control and the mutant mice (S21). However, the results alone may not account for the DPOAE threshold increase^80^.

### Hair cell bundle structure in *Pz1*^*MU*^ and *Pz2*^*MU*^ mice

**Figure 6.**
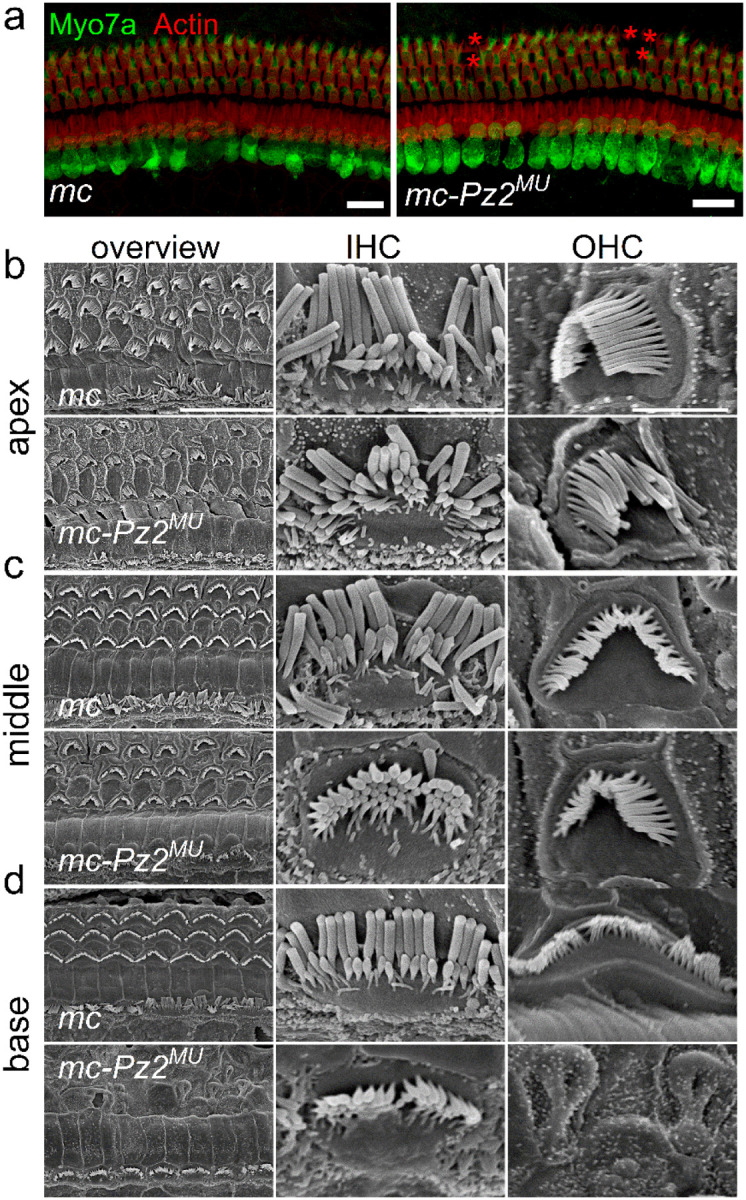
Confocal and scanning electron images comparing cochlear HCs of control vs. *Pz2-ki*^*m-c*^ mice. **a, Left panel,** Whole-mount isolated cochlea from a P28 *m-c* mouse, used as a control sample, showing one row of IHCs and three rows of OHCs, **Right panel**, Similar preparation from a 4-week-old *mc-Pz2*^*MU*^ cochlea. Note (* in red) indicating lost OHCs. Scale bar = 10 μm. **b-d,** Overviews (left columns) showing the apex, middle, and base of 3-week-old control mice with equivalently-treated 3-week-old *mc-Pz2*^*MU*^ mice. Images show nearly identical IHCs and OHCs, although OHCs at the base are missing in certain areas in the *mc-Pz2*^*MU*^ (bottom right). Comparisons at higher magnification of IHCs (middle) and OHCs (right) are near normal in control and *mc-Pz2*^*MU*^ mice: scale bar,10 μm, left, and 2 μm, middle and right column. Similar results were obtained from 5 control and 7 *mc-Pz2*^*MU*^ mice.

Mechanoelectrical transduction appears to be a requisite for HC maturation^81^, and with dwindling current magnitude in *Pz*^*MU*^ HCs, we expected HC morphological alterations and degeneration. We counterstained HC-stereocilia actin with phalloidin-TRITC in P12 and 4-week (P28)-old cochleae. We observed no gross changes in hair bundle structure and the characteristic three rows of OHCs and one row of IHC in the *mc-Pz*^*MU*^ P12 cochlea (data not shown), but by 4-week, loss of OHCs was apparent in the cochlea (Fig. 6a). We used scanning electron microscopy (SEM) for high-resolution analyses to evaluate HC and bundle morphology. Cochlear HCs of *mc-Pz*^*MU*^ mice at P21 had normal morphology, including intact hair bundles and stereocilia linkages in both IHCs and OHCs resembling controls (Figs. 6b–d), but the loss of a few OHCs was apparent at the basal cochlea at P21 (Fig. 6d). In older *mc-Pz*^*MU*^ mice >P42, IHC enlargement, and multiple OHC loss was evident (S20). Profound OHC degeneration at the cochlear basal and middle turns was observed at P56 and older, and other hair bundle abnormalities were noticeable, such as fused OHC stereocilia and basal-turn IHC degeneration (S20).

### Co-immunoprecipitation of Pz and MET complex proteins

**Figure 7.**
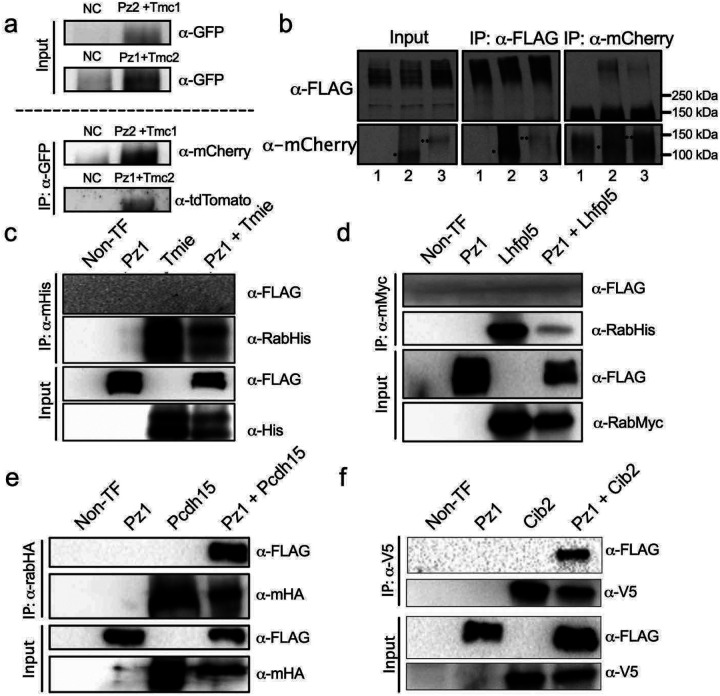
Pz1/2 exists in a complex with Tmc1/2 in cochlear tissue and forms a protein complex with MET complex proteins. **a**. Representative immunoblots of GFP input and tdTomato and mCherry after IP. **b,** Co-immunoprecipitation of FLAG-tagged Pz1 and mCherry-tagged Tmcs. Pz1-FLAG vector was transfected into HEK293 cells in combinations with mCherry alone or Tmcs-mCherry. Immunoprecipitation and western blot analysis were performed with anti-flag and anti-mCherry antibodies of a cell transfected with various combinations as listed (Lane 1: mCherry + Pz1-FLAG, lane 2: Tmc1-mCherry + Pz1-FLAG, lane 3: Tmc2-mCherry + Pz1-FLAG). Lane 1 showed a negative control. Whole-cell lysates were subjected to immunoprecipitation (IP) with a FLAG or mCherry antibody, followed by Western blotting (WB) with FLAG or mCherry, as indicated in the figure. (*=Tmc-mCherry monomer, **=Tmc2-mCherry monomer). Co-IP assays showing the interaction between Tmc1/2-mCherry and Pz1-FLAG (**b**), Tmie-His and Pz1-FLAG (**c**), Lhfpl5-Myc, and Pz1-FLAG (**d**), Pcdh15-HA and Pz1-FLAG (**e**) Cib2-V5 and Pz1-FLAG (**f**) and Tmc1- mCherry and Pz1-FLAG (**e**). Proteins were extracted from HEK 293 cells that transiently expressed target proteins. Protein extracts were incubated with α-FLAG and α-mCherry (**b**), α-His (**c**), α-Myc (**d**), α-HA (**e**), and α-V5 (**f**). Pull-down samples were detected using α-FLAG to identify Pz and α-mCherry to detect Tmc1 (**b**) α- His to detect Tmie (**c**), α-Myc to detect Lhfpl5 (**d**), α-HA to detect Pcdh15 (**e**), α-V5 to detect Cib2 (**f**) in western blotting.

We reasoned that if the Pz protein is an integral and central component of the MET protein complex, it should co-immunoprecipitate as a complex in cochlear tissue. Cochlear tissue was harvested from *Tmc2-AcGFP:Pz1-tdT* and *Tmc1-mCherry*:*Pz2-GFP* mice, whereas negative controls were from non-transgenic mice cochlear tissues. Results show Pz1 co-immunoprecipitated with Tmc2, anti-GFP, and as demonstrated in immunoblot using anti-tdT antibodies. Similarly, to determine whether Pz2 forms multiprotein complexes with Tmc1, we performed immunoprecipitation using anti-GFP and immunoblotting using anti-mCherry antibodies. We show that Pz1 and Tmc2, and Pz2 and Tmc1 interact in a complex (Fig. 7a). Because of limited cochlear tissue and unreliable Pz and Tmc protein antibodies, we were compelled to use the HEK 293 expression system and tagged MET apparatus proteins. Results from HEK 293 cells transiently transfected with either mCherry-Tmc1 or mCherry-Tmc2 confirmed the mCherry-tagged Tmcs pulled-down and anti-mPz1 labelled Pz1, given that HEK 293 cells endogenously express Pz1 (Fig. 7b)^82^. A complement experiment using cells transfected with mCherry-Tmc1/2 and Flag-mPz1 showed positive pull-down with anti-mCherry or anti-Flag, revealing the interaction between mTmc1/2 and mPz1. Next, similar experiments were performed using tagged proteins, including His-Tmie, Myc-Lhfp15, HA-Pcdh15, and V5-Cib2 co-expressed individually with Flag-mPz1. Co-immunoprecipitation investigations revealed that Flag-mPz1 successfully immunoprecipitated with His-Tmie, Myc-Lhfp15, HA-Pcdh15, and V5-Cib2, suggesting the proteins form complexes (Fig. 7c–f). The original gel blots are presented in Supplement Fig. 22 (S22) for disclosure and evaluation. The findings demonstrate that the Pz protein subunit forms a component of the MET complex proteins in cochlear tissue and heterologous expression systems and serves a central role in interacting spatially and functionally with recognized members of the HC MET complex.

## Discussion

The sound and balance gateways to the brain are through MET-channel activation in HC stereocilia. The identity of HC MET channel-complex composition has been a puzzle, and multiple attempts have revealed essential elements of the protein complex, but the pore-forming protein remains debatable^13^. We demonstrate that IHCs and OHCs express *Pz1* and *Pz2* transcripts. Pz1 and Pz2 proteins are localized at stereocilia tips and sides of the adjacent neighboring stereocilium in IHCs, OHCs, and HCs of the vestibular end organ. The second and third OHC stereocilia rows house Pz1 and Pz2 channels. I_Pz_ activation, decay kinetics, and Ca^2+^ permeation are consistent with HC MET channels^4^. Solitary Pz1 and Pz2 proteins are concentrated at the apical and cuticular plate membrane. The co-localization of Pz1 and Pz2 in the stereocilia raised the possibility that the two subunits may form heterotrimers, as shown *in silico* (S2-3)^46^. The formation of a functional heterotrimeric channel may explain the findings that the knockout of one Pz subunit was insufficient to produce robust auditory and vestibular traits^49^.

Using knockin mice harboring *Pz1/2* with reporter gene fusion proteins, we document that Pz1/2 co-localized and formed multiprotein complexes with Tmc1/2 in vestibular and cochlear HCs. In HEK 293 cells, Pz1 forms multiprotein complexes with Tmie, Lhfp15, Pcdh15, and Cib2, all documented components of the MET complex^13^, demonstrating the central role of Pz in the channel complex. The MET complex likely consists of a cadre of interacting proteins, regulating the pore-forming proteins and facilitating the interactions with the tip link and cytoskeleton or plasma membrane lipids to shape the force transmission to the channel. Tmc, in particular, may enrich the local Pz environment with lipid microdomains that may define the MET channel physiological traits and functional interactions to confer the current and its pharmacological sensitivity to DHS. Pz subunits form a non-specific cationic channel with biophysical and pharmacological features resembling the MET currents. Since Pz1 and Pz2 likely form heterotrimers, using a knockin of a non-functional mutant subunit represents one of the most effective strategies to abolish channel functions, providing the data to support the roles of Pz subunits subserving a pathway for ion permeation in HC stereocilia. The strategy is authenticated since WT knockin at the ROSA 26 locus yielded no abnormal auditory and vestibular traits. Notwithstanding the caveats associated with expression systems, the expression of mTmc1 or 2 alone did not bear a mechanically activated current, but Pz1/2 did. Co-transfection of *Pz1/2* with *Tmc1/2* produced a mechanically-gated current with increased density and varied decay kinetics compared with *Pz1/2* alone. *Pz1/2* and *Pz-Tmc* co-expression yielded current with MET pharmacological chracteristics, Ca^2+^-sensitivity, and permeation properties reminiscent of HC MET current^20, 57, 83^.

The null deletion of *Pz1/2* alone in HCs had a mild effect on auditory and vestibular functions^49^, whereas knockin of mutant *Pz1* or *Pz2* subunits produced ABRs and VsEPs with increased thresholds and profound deafness by ~3 months accompanied by HC degeneration. Mice expressing mutant Pz1 and Pz2 were deaf by eight weeks and exhibited severe imbalance. Moreover, IHCs and OHCs expressing *Pz* mutants have reduced FM1–43 uptake with attenuated HC transduction currents, substantiating the assertion that Pz is necessary for HC MET current. Similar results using two distinct HC Cre lines (*Myo15* and *Calb2*) support the conclusion that Pz subunits are essential components of the MET apparatus. HC Pz and Tmc proteins form multiprotein complexes, and the results were confirmed in HEK 293 and N2A cells.

Evidence for HC *Pz2* transcripts and protein expression was previously shown^49, 50^, but detection was missed for Pz1^49^. A more sensitive *in situ* hybridization strategy, smFISH^84^, allowed for *Pz1* and *Pz2* RNA detection and quantification in HCs, and high-resolution microscopy enhanced by protein-fluorophore fusion expression revealed Pz and Tmc proteins localized at the stereocilia tips and sides in propinquity (Figs 1–2). Given the close localization of Pz1/2 in HC stereocilia, predicted structural and functional interaction between the two subunits in HC and N2A cells, and the trimeric structure of the Pz subunits, we sought to attenuate Pz channel function *in vivo* using non-functional *Pz1* and *Pz2* mutants. The auditory and vestibular phenotypes of the *Pz1*^*MU*^, *Pz2*^*MU,*^ and double ki *Pz1–2*^*MU*^ mice are consistent with their roles in MET function. Considering a stochastic assembly of channel subunits and assuming functional hetero- and homomeric channels, and equal levels of subunit expression, ~30% of HCs may carry functional Pz subunits in the single mutant allele knockin HCs, and the functional-channel numbers decrease to ~12% in the *Pz1–2*^*MU*^ cochlear HCs (S23). The analysis may explain the residual hearing and vestibular traits in the single *Pz*^*MU*^ mice in contrast to the early and severe traits in double *Pz1–2*^*MU*^ mice (Fig. 4).

Tmc is an integral element of the MET complex in mammalian HCs^29^ and may serve as ion channels in invertebrates^36^. Cell lines expressing Tmc provide evidence for Pz1 interacting with MET complex proteins, including Tmie, Lhfp15, Pcdh15, and Cib2, indicating that the Pz subunits are central to the MET complex in both cochlear tissue and a reconstituted system. Tmc may be an essential allosteric regulator for Pz-channel functions in mammalian HC. Among the prevailing evidence that Tmc serves as the pore-forming subunits for MET channels is a set of elegant experiments in which specific Tmc1 residues were mutated into cysteine, and cysteine-modification reagents were able to mediate MET currents^29^. In a scenario where the Pz-channel complex was intact except for the mutated Tmc1, it is conceivable to use cysteine modification to restore the MET complex function. Suppose Tmc is an allosteric anchor for force transmission through lipid interaction with Pz and is required for Pz engagement in the HC setting. In that case, results from the previous report can be envisioned. An improbable setup for two parallel ion permeation pathways, one for the Tmcs and the other for the Pzs, can be envisioned and tested in the future. However, the simplest explanation for the current findings is that the Pz channel is the central pore-protein, and a vital allosteric regulatory protein, such as Tmc, sculpts the MET current properties. From the findings, Pz-Tmc current sensitivity to aminoglycosides also offers persuasive evidence for the importance of Tmc in the function of Pz in HCs. The findings provide a compelling alternative for the MET channel identity, Pz protein forming a central pore and complexing with recognized components of the MET channel elements to confer its gateway function for sound-balance-brain communication.

## Experimental Procedures

### Animal Models

#### Generation of genetically modified mouse strains

All animal experiments were performed under the University of Nevada Reno Institutional Animal Care and Use Committee guidelines. Equal numbers of males and females were used in the experiments. In cases where odd numbers are reported, females outnumbered male samples. To generate the *Myo15-Cre* mouse line, a “P2A-Cre-T2A- E2-Crimson” cassette was targeted to replace the TGA stop codon in exon 66 of the *Myo15* gene. The *Calb2-Cre* mouse line was created by inserting a “P2A-Cre-IRES-TagBFP2” cassette upstream of the TAA stop codon in exon 11 of the *Calb2* gene.

For Piezo1 genotyping, we developed a PCR assay (35 cycles) with a pair of primers: (607 bp: F:5”-AAGCACGTTTCCGACTTGAGTTG-3” and R:5”-GGGTGAGCATGTCTTTAATCTACC-3”). The primers for the mutant were: (356 bp: F:5”-TTCAGGGTCAGCTTGCCGTAG-3” and R:5”- CTGCCGTGTGTGGACCGCATCCT-3”) (Fig. 4c). The mutant Peizo2 primers were: (199 bp: F:5”- ACCTCCTCGCCCTTGCTCACCAT-3” and R:5”-TCGTGAGGGAGACAGGGGAGTTG-3”) (Fig. 4d). *Myo15-cre* PCR assay was performed with a pair of primers for wild-type, wt: (435 bp: F:5”-GGGCTTGGCAGTGGTAGTGGTAT-3” and R:5”-GTCACTTGGTCTGGAGAGGCTG-3”), and homozygote, homo: 729 bp: F:5”-CCCAGGAAGCAGTTTAGCAGTG-3” and R:5”-TTTGGTGTACGGTCAGTAAATTGGAC-3”)). *Calb2-cre* PCR assay was performed with a pair of primers for wild-type, wt: (525 bp: F:5”-GTTGATAGGAAGGTCCATTCGGT-3” and R:5”-CAGAAGCCTAAATCATACAGCGAAG-3”), and homozygote, homo: 271 bp: F:5”- ACTTAAACCCACTCTCACCTCTTT-3” and R:5”-TACGGTCAGTAAATTGGACACCTT-3”)) (Supplement Fig. 7). Other mouse lines were purchased from Jackson Labs (Bar Harbor, Maine, USA). Piezo1-tdTomato https://www.jax.org/strain/029214, B6;129-Piezo1tm1.1Apat/J, strain #:029214; Piezo2-GFP https://www.jax.org/strain/027719, B6(SJL)-Piezo2tm1.1(cre)Apat/J, strain #:027719; Tmc1-mCherry https://www.jax.org/strain/028392, B6.Cg-Tg(Tmc1/mCherry)2Ajg/J, strain #:028392; and Tmc2-GFP https://www.jax.org/strain/028517, B6.Cg-Tg(Tmc2/AcGFP)3Ajg/J, strain #:02851; and Ai0-tdTomato https://www.jax.org/strain/007909, B6.Cg-Gt(ROSA)26Sor^tm9(CAG-tdTomato)Hze/J^. Genotype followed primers recommended by Jackson Labs.

### Immunofluorescence

Cochleae were dissected from the temporal bone and fixed with 4% paraformaldehyde in phosphate-buffered saline (PBS) for 2 hrs on the rocker at 4°C. The samples were decalcified in 0.25 M ethylenediaminetetraacetic acid (EDTA) solution for up to 3 days (duration was age-dependent) with daily solution changes. Decalcified cochleae were washed in PBS and micro-dissected to the apex, middle, and base. The utricle was dissected. For the cochlea, the tectorial membrane and Reisner’s membrane were removed. Each sample was permeabilized in 0.05% Triton X-100 in PBS for 10 min and then incubated for 60 min in a blocking solution containing 10% bovine serum albumin (BSA) or 10% goat serum in PBS containing 0.1% Triton X-100. The samples were incubated with the primary antibodies overnight at 4°C. The rinsed tissues were incubated (2 hrs; RT) in a fluorescent dye-conjugated secondary antibody. The following primary and secondary antibodies were used: Anti-GFP antibodies (Abcam), anti-mCherry antibodies (Abcam and Novous), anti-tdTomato (MyBioSouce.com), anti-Myo7a (Proteus), anti-Piezo1 (Novus), Alexa Fluor^™^ 488 goat anti-rabbit, Alexa Fluor^™^ 647 goat anti-rabbit, Alexa Fluor^™^ 647 donkey anti-goat IgG, Alexa Fluor^™^ 488 goat anti-mouse IgG1, Alexa Fluor^™^ 568 goat anti-mouse IgG2a, Alexa Fluor^™^ 568 goat anti-chicken (Invitrogen), and Phalloidin (Abcam and Sigma-Aldrich). Images were captured with Leica SP8 confocal microscopes.

### Single-Molecule Fluorescence *in situ* Hybridization with RNAscope

Mice were anesthetized and transcranial perfused with Diethyl Pyrocarbonate (DEPC)-treated PBS at P10. The temporal bones were isolated and fixed with 4% PFA in DEPCtreated PBS for 24 hrs at 4°C to preserve RNA. The samples were decalcified in bone decalcification buffer (ACD) overnight at 4°C. Decalcified cochleae were washed in DEPC-PBS three times. The cochlear samples were sequentially dehydrated in 10, 20, and 30% sucrose solution at 4°C for 1 h, 2 h, and overnight, respectively, then embedded in OCT for cryosection. Samples were cryo-sectioned to a thickness of 10 μm, placed onto Superfrost slides, and stored at −80°C until processed. Probe hybridization was performed according to the manufacturer’s instructions (Advanced Cell Diagnostics, ACD). Sections were immersed in pre-chilled 4% PFA for 15 min at 4 °C. Sections were then dehydrated at RT in 50%, 70%, and twice in 100% ethanol for 5 min each and allowed to dry for 1–2 min. Fixation and dehydration were followed by protease digestion, using protease for 30 min at RT. Sections were then incubated at 40 °C with the following solutions: (1) target probe in hybridization buffer A for 3 h; (2) preamplifier in hybridization buffer B for 30 min; (3) amplifier in hybridization buffer B at 40 °C for 15 min; and 4) label probe in hybridization buffer C for 15 min. After each hybridization step, slides were washed with washing buffer three times at RT. The label probes were conjugated to Alexa Fluor 488 and 594 for fluorescent detection. Probes, positive and blank negative controls obtained from ACD are shown (Supplement Fig. 1 (S1)). Sequences of the target probes, preamplifier, amplifier, and label probe are proprietary. Detailed information about the probe sequences can be obtained by signing a non-disclosure agreement provided by the manufacturer. Incubation in DAPI solution for 15 s at RT was performed to label cell nuclei. Slides were then mounted in Fluoromount-G and sealed under a coverslip. Images were captured with a Leica SP8 confocal microscope. Dots in each fluorescent-positive cell were counted and scored as described.

### Auditory brainstem responses (ABR) and distortion product otoacoustic emissions (DPOAEs)

Mice were anesthetized using ketamine (40 mg/kg) and xylazine (10 mg/kg). Animals were then placed in a sound-attenuated chamber. Body temperature was maintained at 36.5 ± 0.5°C using a homeothermic blanket control unit (Harvard Apparatus) and rectal probe feedback. Ground and recording electrodes were placed subcutaneously in the scalp, and a calibrated transducer (Tucker Davis) was placed in the right pinna. At a rate of 20 Hz, with intensity from 0 to 90 dB sound pressure level (SPL; rms for click stimuli) in 5 dB increments, 0.1 ms broadband clicks, and 3 ms pure tone pips at 4, 8, 16, and 32 kHz were presented. The ABR activity was extracted from 128 to 1024 stimuli. The hearing threshold was the minimum sound intensity eliciting a typical ABR waveform. For DPOAE experiments, mice were anesthetized with avertin for ABR measurements. After visual inspection to ensure a healthy external and middle ear, the mice were placed in a sound-attenuated chamber, and a dual acoustic probe/microphone assembly (Etymotic Research) was placed in the ear. Primary tones with an f2/f1 ratio of 1.25 were presented at equal sound pressure levels, and tones were routed to independent transducers and allowed to mix acoustically in the ear canal. A digital signal processor sampled and synchronously averaged the SPL for geometric mean frequencies <20.1 kHz. Above 20.1 kHz, the sampling input was automatically switched to the dynamic signal analyzer for frequency analysis to avoid DSP aliasing artifacts >22.1 kHz. Cubic (2f1–f2) DPOAE levels and corresponding noise floor levels were calculated. DP thresholds were generated for each stimulus level and were evaluated relative to the noise floor (DPOAE > 5 dB above the noise floor).

### Vestibular sensory evoked potential measurement (VsEP).

Mice were anesthetized using ketamine (40 mg/kg) and xylazine (10 mg/kg), and VsEP measurements were recorded as described previously^86^. The skull was stabilized with a head mount. Stimuli were linear acceleration pulses of 2 ms duration, nine pulses per second, presented in standard and inverted directions. Normal polarity was defined as the upward displacement of the shaker platform while inverted in the downward platform displacement. Stimulus amplitude was measured in jerk (da/dt, i.e., g/ms, where 1.0 g = 9.8 m/s^2^ and 1.0 g/ms = 9.8 μm/ms, using a calibrated accelerometer attached to the shaker platform. Stimulus amplitude ranged from −18 to +6 dB re: 1.0g/ms, adjusted in 3-dB steps. Two-channel signal averaging was used to resolve vestibular responses from background electroencephalographic activity. The electrophysiological activity was amplified (200,000X), filtered (300 to 3000 Hz), and VsEPs were recorded to normal and inverted stimulus polarities (1024 points, 10-μs per point, 128 responses per averaged waveform). Three response parameters were quantified: threshold, peak latencies, and peak-to-peak amplitudes. The threshold is measured in dB re 1.0 g/ms and is defined as the stimulus amplitude midway between that which produced a discernible VsEP and that which failed to produce a response. The threshold measures the sensitivity of the gravity receptor end organs, utricle, and saccule. Thresholds, latencies, and amplitudes are averaged for each group and analyzed using variance (one-way ANOVA).

### Cell culture and plasmid vector transfection

Neuro2A cells (N2A) were grown in Eagle’s Minimum Essential Medium (EMEM) containing 10% fetal bovine serum and 50 units/ml penicillin at 37 °C with 5% CO_2_. Cells were plated onto 35 mm dishes or 15-mm round glass coverslips placed in 4-well plates and transfected using lipofectamine 3000 (Invitrogen) according to the manufacturer’s instruction. For the Pz overexpression experiment, 1 μg/ml of mPz1-IRES-GFP or vector only was transfected, and cells were recorded 24–48 hours later. The initial mPz1 and mPz2 clones were a generous gift from Dr. A. Patapoutian.

### In vitro and ex vivo fluorescence resonance energy transfer (FRET) experiments

Mouse Pz1 tagged with mClover3 (mPz1-mClover3) and mTmc1 tagged with mRuby3 (mTmc1-mRuby3) were transiently transfected into cells using Lipofectamine 3000 (Invitrogen) following the manufacturer’s instructions. For further immunofluorescence microscopy and FRET experiments, cells were passaged and seeded on 35-mm glass bottom dishes for imaging 2–3 days after transfection. Before FRET imaging, the cell culture media in the dishes was replaced with Tyrode’s solution containing (in mM): 144 NaCl, 1 MgCl, 4 KCl, 2 CaCl2, 10 D-Glucose, 0.33 NaH_2_PO_4_, 10 Hepes with pH 7.4. Zeiss LSM 700 microscope was used for the imaging. The Pz1-mClover3 (donor) and mTmc1-mRuby3 (acceptor) co-transfected N2A cells were excited by 488 nm and 555 nm laser, respectively. For photobleaching of the acceptor signal, a specific surface membrane region (region of interest (ROI)) was chosen and photobleached with a 555-nm laser, and simultaneous donor emission signals excited with a 488-nm laser were acquired using time-lapse mode till steady state recording. The intensity increase of the donor emission was calculated by subtracting the background (averaging of 3 data points) from the steady-state averaged data (3 data points). The FRET efficiency (E) was calculated based on the donor intensity pre (D_pre_) and post (D_post_) acceptor photobleaching: E= (D_post_-D_pre_)/D_post_. To minimize the photobleaching effects of the donor on the FRET quantification, we used only donor cells for acceptor bleaching control, and a linear equation fitted the donor bleaching time course to obtain the slope, which was used for correction of the time course of the donor signal in the co-expression experiments.

### FM1–43 dye loading

A stock solution, 10 mM, was prepared in PBS. A new working solution was prepared. Cochlear samples were incubated in 10 μM of FM1–43 for 5 s, washed with PBS, and observed under the Leica SP8 confocal microscope as described^19^.

### Scanning electron microscopy (SEM)

Mice were perfused with 4% PFA in 1x PBS, the inner ears were isolated, and the stapes footplate was removed. The ear was flushed with, then fixed overnight in 4% paraformaldehyde (PFA), and 2.5% glutaraldehyde in 1x PBS. After washing in ddH_2_O for 3X in 1-hour, the samples were post-fixed with 1% osmium tetroxide for approximately 1 hour. The sample was washed in PBS before decalcifying for 3–4 days in 0.25 M EDTA at 4°C with daily solution changes. The cochleae were microdissected, the tectorial membranes removed and gradually dehydrated using 30%, 50%, 70%, 80%, 90%, 100% ethanol, 2:1 ethanol/ hexamethyldisilazane (HMDS) (Thermo Scientific #A15139 AE), 1:2 ethanol/HMDS, and finally 100% HMDS. Samples were transferred to an open well plate in HMDS and allowed to air dry overnight in a fume hood. Samples were then mounted on aluminum stubs (Ted Pella #16111) using double-sided carbon tape (EMS #77817–12) and stored in a specimen mount holder (EMS #76510) and sealed in a desiccator until sputter-coated with Au/Pd (Emitech Sputter Coater K550). Samples were viewed and images captured under SEM (Hitachi S-4800- University of Iowa Central Microscopy Research Facilities). An accelerating voltage of 5-kV and 11-kV were used (Supplement Fig. 10 (S10)). Images were compiled using CorelDRAW X7 graphic software.

### Co-immunoprecipitation

We homogenized and sonicated cochlear tissues in Pierce immunoprecipitation lysis buffer and incubated the lysates with GFP-antibody at 4°C overnight. The next day, magnetic beads were prepared according to the manufacturer’s instructions (ThermoFisher). We then incubated the sample/antibody with Dynabeads Protein G for 30 mins at RT to allow the antigen-Ab complex to bind to the magnetic bead. A magnetic strip was used to remove the supernatant. The beads were then washed 3 times with a washing buffer. An elution buffer was used to elute the antigen of interest. Samples were heated for 10 mins at 70° C before immunoblotting. The primary antibodies used for immunoblots were: anti-mCherry (Abcam, ab183628), 1:1000, anti-GFP (Abcam, ab6556), 1:1000, and anti-tdTomato (OriGene, TA183027), 1:1000.

Plasmids (m*Pcdh15, mTmie, mLfhpl5, mCib2,* and m*Tmc1*) were purchased from Gene copoeia. HEK 293 cells and lipofectamine were used for plasmid overexpression. IP lysis buffer (Thermo Scientific, cat no. 87787) and dynabeads Protein G immunoprecipitation kit (Invitrogen, cat no. 10007D) were used for immunoprecipitation. 3.48×10^6^ cells/well were seeded in a 100 mm dish 1-day before the transformation. Transfected cells were harvested 48 hours after transformation, then lysate with IP lysis buffer. The lysate sample was centrifuged, and the supernatant from the lysate sample was used in the reaction. Antibody and dynabeads G were incubated for 10 min at RT to make antibodies conjugate to beads. Antibodies conjugated beads were washed twice and incubated with the supernatant from cell lysis for 20 min at RT. The sample was washed 3 times and transferred to the new tube. The transferred sample was washed one time, and all of the washed solutions were removed from the sample. The elution buffer was incubated with the sample for 5 min at RT and boiled with 5x loading dye at 70°C for 10 min. The boiled sample was electrophoresed in 10% Mini-Protean TGX precast protein gels (Bio-Rad, cat no. 4561033). The sample was transferred to PDVF and then incubated with 5% skim milk for 1hr. The primary antibody was reacted with the sample transferred membrane Overnight at 4°C and was washed 3 times with 1% TBST. The secondary antibody was incubated with the membrane for 2 hr and was washed 3 times. The membrane was incubated with Clarity western ECL substrate (Bio-rad, cat no. 1705060) and the signal was detected with ChemiDoc MP imaging system (Bio-Rad). Plasmids from Genecopoeia were as follows: mouse Pdch15 (EX-Mm28779-M07-GS), mouse Tmie (EX-Mm05449-M77-GS), mouse Lfhpl5 (EX-Mm30626-M09), mouse Tmc1 (EX-Mm02159-M56) and mouse Cib2 (CS-Mm07673-M02–01J). Antibodies were as follows: anti-HA (Abcam, cat no. ab9110, ab18181), anti-His (Abcam, cat no. ab18184, ab9108), anti-Myc (Abcam, cat no. ab32, ab9106), anti-mCherry (Abcam, cat no. ab183628, ab125096) and anti-Flag-HRP (Abcam, ab49763).

### Electrophysiology

Patch-clamp experiments were performed in a standard whole-cell configuration using an Axopatch 200B amplifier (Axon Instruments). Patch pipettes had a resistance of 2–3 MΩ when filled with an internal solution consisting of (in mM) 133 CsCl, 10 HEPES, 5 EGTA, 1 CaCl_2_, 1 MgCl_2_, 4 MgATP, and 0.4 Na_2_GTP (pH adjusted to 7.3 with CsOH). The extracellular solution consisted of (in mM) 130 NaCl, 3 KCl, 1 MgCl_2_, 10 HEPES, 2.5 CaCl_2_, 10 glucose (pH was adjusted to 7.3 using NaOH). Currents were sampled at 20 kHz and filtered at 2 kHz. Voltages were not corrected for a liquid junction potential. Leak currents before mechanical stimulations were subtracted offline from the current traces. 10 mM dihydrostreptomycin and neomycin stock solution were prepared in water. All electrophysiological experiments were performed at RT (21–22°C). Reagents were obtained from Sigma Aldrich unless otherwise specified.

### Mechanical Stimulation.

Mechanical stimulation was achieved using a fire-polished glass pipette (tip diameter 1-μm) at 60° to the recorded cell. Downward movement of the probe toward the cell was driven by a Clampex-controlled piezo-electric crystal micro stage (E660 LVPZT Controller/Amplifier; Physik Instruments). The probe was typically positioned close to the cell body without visible membrane deformation. Mechanical displacement was applied with a fire-polished blunt pipette driven by a piezo-electric device. To assess the mechanical sensitivity of a cell, a series of mechanical steps in ~140 nm increments were applied every 10 to 20 s, which allowed for full recovery of mechanosensitive currents. Inward MA currents were recorded at a holding potential of −80 mV. For I-V relationship recordings, voltage steps were applied 750 ms before the mechanical stimulation from a holding potential of −60 mV.

For mechanical stimulation of hair bundles, we employed stepwise and sinewave stimulation of OHCs and IHCs using a piezo-driven fluid-jet hair bundle mechanical stimulator on P10-P12 cochleae and recorded IHC and OHC MET currents in the whole-cell configuration. We represented the bundle displacement in the form of applied piezo-driven voltage. Bundle displacement was not calibrated for each cell because of variations in stimulating probe positions relative to the stimulated hair bundle.

### Estimating the pore size

We followed the approach used for the MET channel in hair cells to estimate the Piezo1 pore size from the inner face and amines radius. Plots were fit empirically to

(1) $$I_{X}/I_{Cs}=A\left(1-a/r^{2}\right)$$

where *a* is the radius of the amines, *r* is the radius of the channel, and *A* is a scaling factor. Internal solutions were made with monovalent amines of different sizes, with size estimated as previously described from CPK models^87, 88^

The external face pore size data were fit to

(2) $$I_{X}/I_{Na}=(A{\left(1-a/r\right)}^{2}/a$$

where I_x_/I_Na_ is the current ratio, *a* is the radius of the amine compound, *r* is the radius of the channel, and *A* is a scaling factor^58^. The external solution used the same approach to substitute Na^+^ with amines of different sizes. Amines were purchased from Sigma Aldrich.

### Single-channel recordings and analysis

Single channel gating properties were recorded using a Planar Lipid Bilayer Workstation (Warner Instruments LLC, Hamden, CT). A bilayer lipid membrane (BLM) was formed across a 200-μm diameter aperture drilled within a polysulfone cup position within a BLM chamber. The composition of the BLM was either a synthetic lipid blend composed of phosphatidylethanolamine/phosphatidylserine/phosphatidylcholine (PE/PS/PC) (5:3:2 w/w; Avanti Polar Lipids, Alabaster, AL) or a brain total lipid extract dissolved in 30 mg/ml n-decane (Acros, New Jersey). The BLM formed two electrically isolated liquid-filled sides of chamber (0.8 ml each), defined as *cis* and *trans* sides, respectively. Voltage input was applied to the *trans* side, whereas the *cis* was grounded. Piezo enriched (purified) protein preparation, ~50–70 mg/ml, and test reagents were always added on the *cis* side solution (500 mM KCl, 10 mM MOPS, pH 7.4), stirred constantly. An initial gradient of 5:2 K^+^ *cis* vs. *trans* was used to promote incorporation of channel protein into the BLM. Once channel incorporation took place, the *trans* chamber was immediately perfused with 500 mM KCl to establish symmetrical ionic conditions across *cis/trans* to prevent additional channel fusions. The amplified current signals were recorded at the indicated holding potentials, filtered at 800 Hz (low-pass Bessel filter 8-pole; Warner Instruments), and digitized at a sampling rate of 10 kHz (Digidata 1440; Molecular Devices, Sunnyvale, CA). Channel data was acquired with pClamp 10 software (Molecular Devices, Sunnyvale, CA). Data analysis and plots were made with OriginPro 2018b and Corel Draw 2020.

### Sequence and structural evidence for Pz1 and Pz2 heterodimers:

To assess whether Pz1 and Pz2 could form heterodimers, we explored the concept of conservation of salt bridges as the matching of positive and negative charges is widely used as a tool to design drug and protein ligands. Using the Pz1 structure (PDB: 6BPZ), we selected one chain (**see** S2) (**a**) of the Pz1 structure and expanded our selection to include any amino acid on the remaining two chains (**b, c**) within 5 Å. We then kept this selection on the two chains (**b, c**) and expanded this selection to any amino acid on the original chain (A) by 5 Å to back-select amino acids on chain (A) to obtain all inter-chain interacting amino acids in the Pz1 trimer. We then selected the basic and acidic amino acids and mapped these onto the Pz1 mouse structure (S2-3). We then calculated a local sequence alignment between mouse-Pz1, human-Pz1, mouse-Pz2, and human-Pz2. The local alignment included the trimer interacting amino acids and surrounding amino acids to align the interacting amino acids properly. We determined the conservation of the interacting amino acids, which reports a 74.59% conservation and an 83.6% amino acid chemical property conservation (S3). The sequence on Pz2 that matched the Pz1 basic and acid salt bridges was also mapped onto the mouse-Pz2 structure (PDB 6KG7). Due to this very high conservation, the chains could be interchanged. We verify that the conserved ionic charges match in 3D coordinates and the location of the non-conserved charges. We replaced Chain B in Pz1 with Chain B from the Pz2 structure using the matchmaker (UCSF Chimera) to make a 2:1 Pz1:Piezo2 structure. We repeated the procedure to replace chain C of Pz1 with chain C of Pz2 to build a 1:2 Pz1:Pz2 model. Visual inspection of these models shows the pore domain contains the most conserved charged residues while the cap domain contains the most non-conserved charged residues (S2). In addition, the 3D positions of the acidic and basic residues have high conservation, suggesting that heterotrimer structures are structurally plausible.

### Statistical analyses

Where appropriate, pooled data are presented as means ± SD. Significant differences between groups were tested using t-test and ANOVA, where applicable. The null hypothesis was rejected when the two-tailed *p*-value < 0.05 is indicated with *, < 0.01 with **, < 0.001 with ***. The number of mice and neurons is reported as *n*.

## Supplementary Material

1 **Supplement Figure 1 (S1). Negative and positive controls in cochlear sections.** smFISH localizes transcripts encoding the *Pz1/2* channels and *Tmc1* (Fig. 1), but not in negative control sections. **a-b**, Negative and positive probes provided by the manufacturer (ACD) were used on cryo-sections of the 2-w-old cochlea. Positive probes for mammalian samples were detected as fluorescent puncta in green and red. Outlines in red (left column) show one row of IHCs and three rows of OHCs. Comparison of control and experimental values are summarized in Fig. 1 and provided in the Results. Scale bar = 10 μm.
**Supplement Figure 2 (S2). Salt bridge specificity is highly conserved in the Pz1 and Pz2 homotrimer interfaces**. **a-d**, Homotrimers of Pz1 (PDB: 6BPZ) from the extracellular (**a**) and transmembrane (**b**) side and Pz2 (PDB: 6KG7) from the extracellular (**c**) and transmembrane (**d**) side. Monomers are colored consistently in panels (**a-d**) with monomer 1 = yellow, monomer 2 = cyan, monomer 3 = pink **e-f**) side view of a heterotrimer with a 2:1 (**e**) and a 1:2 (**f**) Pz1: Pz2 ratio. The protein backbone is shown in a ribbon with basic and acidic amino acids in the sphere. Coloring for Pz1 is as follows: ribbon = yellow, conserved basic = light blue, conserved acidic = red/orange, non-conserved basic or acidic = same color as protein ribbon. Pz2 coloring is as follows: ribbon = purple/pink, conserved basic = dark blue, conserved acidic = dark red, non-conserved basic or acidic = same color as protein ribbon.
**Supplement Figure 3 (S3). Alignments of Pz1 and Pz2 interacting interfaces.** Sequence alignment for Pz1 trimer interface. Alignment includes human Pz1 (Piezo1_H), mouse Pz1 (Piezo1_M), human Pz2 (Piezo2_H), mouse Pz2 (Piezo2_M). The sequence coloring is based on amino acid properties according to the “Taylor” scheme found in Jalview.
**Supplement Figure 4 (S4). Localization of Pz1 and Pz2 within nanometer proximity in hair cells**. **a-b.** The expression and distribution of Pz1 and Pz2 were detected using *Pz1-tdT* and *Pz2-GFP* (*Pz1-tdT/Pz2-GFP)* mice (P10) and assayed using a proximity ligation strategy. Images are shown as Z-projections through a stack of confocal micrographs Pz1 and Pz2 frequently co-localized within less than 40 nm proximity at the stereocilia and cuticular plate membrane in hair cells. The right panels are photomicrographs of the merged images. Scale bar = 3 μm.
**Supplement Figure 5 (S5). Localization of Tmc1 and Pz2 in the IHC and OHC stereocilia. a,** Confocal fluorescence images obtained from *Tmc1-mCherry*:*Pz2-GFP* mice. Tmc1 (red) and Pz2 (green) labeling was strongest at stereocilia tips counter-stained in blue with Alexa-405-phalloidin for actin. Whole-mount cochlea from P10 *Tmc1-mCherry/Pz2-GFP* mice OHCs. The two rightmost panels illustrate Pz2 and Tmc1 labeling at the second (red arrow) and third (blue arrow) rows of OHC stereocilia. Labeling on the first row was rare. Scale bar = 2 μm. **b,** Series of confocal sections at levels 0, 0.6, 1.2, and 1.8 μm, respectively, from stereocilia tips towards the cuticular plate at levels indicated. The inset in the left panel illustrates OHC and the sections’ approximate levels marked with dashed red lines. Scale bar = 3 μm. The rightmost panel is a side view of the OHC stereocilium showing the location of Pz2 and Tmc1 labeled with GFP and mCherry. Scale bar = 0.5 μm. **c,** IHC stereocilia show expression of Pz2 (green) and Tmc1 (red). Scale bar = 5 μm. **d,** Side view of IHC stereocilia showing Pz2 and Tmc1 location. Scale bar = 0.7 μm. Scale bar for the rightmost panel = 1 μm.
**Supplement Figure 6 (S6). Plasma membrane and cytoplasmic expression of mPz1 and mTmc1 in N2A cells. a.** A typical epifluorescent photomicrograph of an N2A cell 48-hrs post-transfection with *mPz1-Clover3* plasmid (left panel) and the corresponding image obtained upon switching to TIRF mode to determine membrane expression (right panel). **b.** Similar images were captured using *mTmc1-mRuby3* plasmid transfection. Scale bar (**a & b**) = 10 μm. **c.** Shown is a summary plot of the ratio of the arbitrary fluorescence in TIRF mode (membrane localization) versus epifluorescence mode (cytoplasmic expression). Data were obtained from 15 cells. Comparing data from **a** and **b,** *p* = *0.039* (*n* = *15*).
**Supplement Figure 7 (S7). Mechanically activated (MA) Pz1 current (I**_**Pz1**_**) and pore-size estimation. a,** Representative traces of MA currents after *mPz1* expression in N2A cells, elicited by a series of mechanical steps of ~70-nm intervals at −80 mV holding voltage. **b,** Normalized current (I/I_max_)-displacement relationship of current fitted with a two-state Boltzmann function. **c,** Mean I-V relationship, using an instantaneous current-displacement protocol. The inset shows MA current traces evoked at different potentials (~1-μm displacement). Membrane potentials were stepped in 30-mV increments from −120 to +90 mV. **d,** Current-voltage plots normalized to the −90 mV current level for different internal amine compounds (left). Current obtained at 120 mV normalized to that at −120 mV plotted against the radius of the intracellular amine as determined from Corey-Pauling-Koltun (CPK) space-filling models. **e,** A plot of normalized current obtained at 120 mV with extracellular Na^+^ and various charge carriers against membrane potential (left). (**f, g**) The solid line represents estimates of inner and outer pore size with data fits showing a pore radius of 7±4 Å and 20±5 Å, respectively.
**Supplement Figure 8 (S8). Internal Ca**^**2+**^ **buffering altered Pz1-current (I**_**pz1**_**) adaptation kinetics but not mechanical sensitivity. a-b**, The current response traces from N2A cells transfected with *Pz1* to mechanical stimuli with different Ca^2+^ chelators, 5-mM EGTA, and BAPTA in the pipette solution. In black are the traces obtained with a hyperpolarized (−80 mV) holding potential, and in blue are traces evoked when cells were held at a depolarized (70 mV) holding potential. **c**, Summary box plot shows current decay (time constant (τ_decay_)) with different Ca^2+^ buffers. The τ_decay_ for maximum current (I_max_), elicited at a −80-mV holding potential in 5 mM EGTA (in ms), 24±3 (n=15), whereas in 5 mM BAPTA, it is 38±4 (n=5). The τ_decay_ at a 70-mV holding voltage in 10 mM EGTA is 71±8 (n=8), and in 10 mM BAPTA, it is 97±10 (n=6). There were significant differences (*p*<0.05) when comparing 5 mM EGTA (5-EGTA), BAPTA (5-BAPTA) at −80 mV, and 10 mM EGTA (10-EGTA) and BAPTA (10-BAPTA) at 70 mV, F(3,30)=(35), *p* = 5.7X10^−10^. *Posthoc* comparisons using a Tukey HSD test indicate significant differences at −80 mV when comparing 5-EGTA vs. 5-BAPTA (*p*=1.3X10^−6^, and 10-EGTA vs. 10-BAPTA (*p*=0.02 at 70-mV when comparing 5-EGTA vs. 5-BAPTA (*p*=0.007) and 10-EGTA vs. 10-BAPTA (*p*=8.0X10^−6^) are significantly different. **d-e,** Normalized current-displacement plots at −80-mV (black) and 70-mV (blue), respectively. Internal solutions containing 5 mM EGTA are represented in squares, and 10 mM BAPTA in diamonds. Using a two-state Boltzmann equation to fit the data, half-maximum displacements X_1/2_ was ~0.85 μm, and the slope at X_1/2_ was ~50 pA μm^−1^. There were no significant differences between the two conditions.
**Supplement Figure 9 (S9). Alterations in mouse Piezo (mPz)-current properties after co-expression of Pz1, Pz2, Tmc1 (T1), and Tmc2 (T2). a**, Family of Pz1, Pz2, and co-transfected Pz1/2 (denoted; I_Pz1_, I_Pz2,_ and I_Pz1/2_) current traces invoked from N2A cell (total DNA, 1 μg). For the *Pz1/2,* a 1:1 molar ratio was used (total, 1 μg). Cells were held at −80 mV. Despite sustained mechanical displacement, visible differences were apparent in the rapid activation and decay. Co-expressed *Pz1/Pz2* plasmid yielded a hybrid current. **b,** Summary of the displacement-response of I_Pz1_ (■), I_Pz2_ (●), and I_Pz1/2_ (▲). Uniformed sigmoidal curves generated from a two-state Boltzmann function showed that the half-activation (X_1/2_, (in μm)) was 0.87±0.01 (n=11), I_Pz2_ was 0.67±0.01 (n=10) and I_Pz1/2_ was 0.76±0.01 (n=12). Displacement, (X_1/2_, pA μm^−1^) sensitivity for I_Pz1_ was 80±8 (n=11), I_Pz2_ was 54±15 (n=10), and I_Pz1/2_ was 72±6 (n=12). **c,** Representative maximum current traces recorded from N2A cells 48-hrs after transfection with *Pz1, Pz2, Pz1/2, Pz1/Tmc1 (T1),* and *Pz1/Tmc2 (T2)* plasmids. The respective decay or adaptation (adapt) time constants (τ) are noted for each trace. **d,** Summary data of τ_adapt_ for the maximum current for the five transfection conditions. Mean τ_adapt_ (in ms) I_Pz1_ = 25±2 (n=14), I_Pz2_ = 6±1 (n=13), I_Pz1/2_ = 17±1 (n=14), I_Pz1/T1_ = 13±1 (n=14) and I_P1/T2_ = 6±1 (n=14). There were significant differences at *p*<0.05 level for the τ_adapt_ of the different transfection conditions F(4,64)=(53) *p* = 1.4X10^−19^. *Post hoc* comparisons τ_adapt_ using the Tukey HSD test indicate that I_Pz1_ vs. I_Pz1/2_ (*p*=1.6X10^−4^); I_Pz2_ vs. I_Pz1/2_ (*p*=3.9X10^−8^); I_Pz1_ vs. I_Pz1T1_ (*p*=3.6X10^−8^); I_Pz1_ vs. I_Pz1T2_ (*p*=0); and I_Pz1T1_ vs I_Pz1T2_ (*p*=1.2X10^−4^) are significantly different. **e,** Co-transfection of *Pz1* and *Tmc1 (T1)* plasmid resulted in a profound increase in the MET current compared with *Pz1* and *Tmc2 (T2)* or *Pz1* alone in N2A cells. Typical I_Pz1_ and I_Pz1/T1_ traces are shown as an inset. The maximum current Imax is 40–42-hrs post-transfection for Pz1, Pz1/T1, and Pz1/T2. Mean values (in pA) for I_Pz1_=1221±181 (n=10), I_Pz1/T1_ = 2920±396 (n=10), I_Pz1/T2_ = 1129±216 (n=10). There were significant differences at *p*<0.05 level for the I_max_ of the different transfection conditions F(2,27)=(13) *p* = 1.2X10^−4^. *Post hoc* comparisons I_max_ using the Tukey HSD test indicate that I_Pz1_ vs. I_Pz1T1_ (*p*=6.0X10^−4^); I_Pz1/T1_ vs. I_Pz1/T2_ (*p*=3.2X10^−4^) are significantly different, but I_Pz1_ vs. I_Pz1/T2_ (p=1.0) is not significantly different. **f,** Summary of the displacement-response of I_Pz1_ (■), I_Pz1/T1_ (●), and I_Pz1/T2_ (●). Uniformed sigmoidal curves generated from a two-state Boltzmann function. For I_Pz1_, the half-activation (X_1/2_, (in μm)) = 0.83±0.02 (n=11), I_Pz1/T1_ = 0.88±0.01 (n=10) and I_Pz1/2_ = 0.86±0.01 (n=12).
**Supplement Figure 10 (S10)** **Interactions between mPz1 and mTmc1 in N2A cells and Pz2 and Tmc1 in OHCs.** mPz1-mClover3 (donor) and mTmc1-mRuby3 (acceptor), co-transfected and co-expressed in N2A cells. The time courses showed the donor fluorescent intensity signal enhancement when the fluorescent acceptor signal was photobleached, showing the functional interaction and proximity of the mPz1 and mTmc1. **a & c.** Photomicrographs of N2A cells expressing mPz1-mClover3 and mTmc1-mRuby3 (**a**) and mouse OHCs expressing Pz2-GFP and Tmc1-mCherry from a mouse. The white circle indicated a region of interest (ROI) where recordings were made. OHCs were isolated from Piezo2-GFP (donor) and Tmc1-mCherry (acceptor) transgenic mice. **b & d.** The time course showed the enhancement of the donor fluorescent intensity signal when the fluorescent acceptor signal was photobleached, demonstrating the interaction and proximity of the mPz1 and mTmc1 in N2A cells and mPz2 and mTmc1 in OHCs. The mean FRET efficiency (E) in N2A cells was 0.10±0.02 (n=8), and in OHCs was 0.10±0.01 (n=7), respectively.
**Supplement Figure 11 (S11)** **Dihydrostreptomycin (DHS) block of I_Pz_ and I_Pz/Tmc1_** **a,** Mechanically-activated (MA) current in N2A cells expressing Pz1 and evoked at −80 mV with 1-μm displacement in control (black) after 100-μM (gray) and 1000-μM (light gray) DHS application. **b,** MA current traces under control and after the indicated DHS application. Cells were held at −80 mV and activated using 1-μm displacement. Traces in black, green, purple, blue, and cyan were control (in μM) 0.05, 0.1, 1, and 2, respectively. **c,** DHS concentration-inhibition response for I_Pz1_ IC_50_ = 270±135 μM (n=7), and I_Pz1/Tmc1_ = 70±6 nM (n=7). Data from neomycin blocked (200 μM) indicated (Δ) ~65% of the total I_Pz1_ (n=2).
**Supplement Figure 12 (S12)** **Pz1 single-channel activity.** **a,** Family of unitary single-channel traces recorded from N2A cell patch under at −80 mV holding potential subjected to −4-mm Hg pressure. **b,** The amplitude histogram of the relative counts versus unitary current amplitude. **c,** Unitary current amplitude determined at different holding potentials assessed from 5 patches from 5 N2A cells and fitted with a linear regression line. The single-channel conductance (γ) was 36±5 pS (n=5).
**Supplement Figure 13 (S13)** **Channel properties of purified Piezo reconstituted in a planar lipid bilayer.** Induced incorporation of Pz1 channels into the bilayer lipid membrane (BLM) from the cis side was recorded at a potential of 100 mV (cis to trans). The recording buffer was symmetric (500-mM KCl, both cis and trans). Channel activities were recorded at the discrete holding potentials indicated or during a programmed voltage ramp protocol. **a-d**, Representative current traces of recordings made from independent channel incorporations at the indicated holding potentials using BLMs formed with synthetic lipid (PE:PS: PC 5:3:2; w/w); The dashed line(s) and their corresponding numbers to the right of the traces indicate the number of channels resolved. The unitary conductance (γ, or G_o_) was obtained from a linear fit from the current/voltage (I/V) relationships (**e**). The results presented in **a-f** were from 2 separate protein preparations. Channel activities recorded with BLM formed with brain total lipid extract are shown in **f-k**. The representative current traces of a 4-channel recording under the indicated holding potentials are shown in **f.** The dashed oval indicates the region of the trace expanded in the inset immediately above (recordings made at 40 mV). The unitary conductance (γ or G_o_) is shown in **g**. Channel activities were recorded before (control period) and following the addition of (**h**) 100 μM DHS, (**i**) 1 mM or 2 μM GsMTx4, or (**j**) 20 μM ruthenium red, non-selective ion channel blockers under a voltage ramp protocol (**k**). The results presented in **f-k** were from 2 separate protein preparations.
**Supplement Figure 14 (S14). The mechanically-activated (MA) current is generated by wild-type and mutant Pz1 and Pz2 channels. a, (left panel)** Wild-type *Pz1* with the amino acid at the C-terminal domain (CTD) MFEE at 2493–2496 expressed in N2A cells. Typical MA current traces generated with stepped displacements from 0 to 2 μm ΔX = 0.4 μm. Panels indicated show current traces generated with similar protocols from N2A cells expressing AAAA amino acid mutation, wild-type *Pz1*, MFEE:AAAA at a ratio of 1:1, and *Pz2*, MFEE:AAAA (1:1). For *Pz2*; the MFEE was at position 2767–2770. **b,** Summary data comparing the maximum current recorded from N2A cells expressing wild-type and mutant *Pz1* and *Pz2*. Mean maximum current for wild-type (in pA) Pz1-MFEE = 1156±142, n=15; Pz1-AAAA = 14±4, n=15; Pz1-MFEE:AAAA-Pz1 = 79±9, n=15; Pz1-MFEE:AAAA-Pz2 = 63±9, n=15. One-way ANOVA, F(3, 56) = (60) *p*=*1.5X10*^−*17*^; *Posthoc* comparisons Pz1-MFEE vs Pz1-AAAA *p*=*3.8X10*^−*7*^; Pz1-MFEE vs Pz1-MFEE:AAAA-Pz1 *p* =*9.5X10*^−*8*^, and Pz1-MFEE vs Pz1-MFEE:AAAA-Pz2 *p*=*9.6X10*^−*8*^.
**Supplement Figure 15 (S15): *Myo15-Cre (mc)* and *Calb2-Cre* (cc) genotype analyses and expression.** **a-b,** PCR genotyping of wild-type and mutant littermate mice for *Myo15-Cre* and *Calb2-Cre* lines. **c,** tdT (red) expression in IHC and OHCs from *Myo15-Cre-Ai9-tdT* P5 mice shows cell-specific identification of the Cre-line. **d,** Postnatal calretinin (Calb2-GFP gene) in mice at P5 shows robust expression in IHCs and moderate expression in OHCs. Myo7a is used as a hair cell marker in cyan, and actin is labeled with phalloidin (red). Scale bar = 10 μm.
**Supplement Figure 16 (S16). Body weight of wild-type (WT) and expression of Pz1 in *Pz-ki* mice. a,** Mean body weight of control (*myo15-cre* and *calb2-cre*) and mc-*Pz1*^*MU*^*, mc-Pz2*^*MU*^*, ccPz1*^*MU*^, and cc-*Pz2*^*MU*^ mice. Data are shown as a box plot with scattered intervals and listed as mean ± s.e.m. Equal numbers were tested for males and females. When odd numbers are reported, the females outnumber males. No significant differences were detected between the sexes. Thus, the reported data were combined. For *myo15-cre* and *Pz ki* lines one-way ANOVA, F(5,78) = (21) *p* = *2.0X10*^−*13*^. *Post hoc* comparisons using the Tukey HSD test indicate that at 4 weeks (body weight in grams (g)), *myo15-cre (mc)* (23±1, n=17) *vs*. mc-*Pz1*^*MU*^ (14±1, n=19) (*p*=4.9X10^−8^); *mc* (23±1, n=18) *vs*. mc-*Pz2*^*MU*^ (14±1, n=12) (*p*=1.2X10^−7^) are significantly different. For *Calb2-Cre (cc)* and the *Pz*^*MU*^ lines, one-way ANOVA, F(3,40) = (3) *p* = 0.03. *Post hoc* comparisons using the Tukey HSD test indicate that at 4-week *cc* (19±1, n=12) vs. *cc-Pz1*^*MU*^ (17±1, n=13) (*p*=0.6); *cc* (19±1, n=8) vs. cc-*Pz2*^*MU*^ (21±1, n=11) (*p*=0.3) are not significantly different. **b**, Immunofluorescence labeling of OHCs of a P21 whole-mount cochlea showing positive reactivity of actin (green), Pz1 (red), and Myo7a (cyan) antibodies. The merged image is shown. Scale bar = 5 μm.
**Supplement Figure 17 (S17). Progression of ABR thresholds to tone pip sounds and clicks from 4 to 8 week old *mc-Pz1/2*^*MU*^ and *mc-Pz1–2*^*MU*^ mice.** **a-c**, Summary of the ABR thresholds to tone pip sounds. **a,** Comparison of ABR thresholds for *mc, mc-Pz1*^*MU*^ in 4- and 8-week-old mice. Mice were littermates, and male and female numbers were equal. When odd numbers are reported, the females outnumber males. We combined the data since no significant differences were detected between the sexes. Data are shown as mean ± SD. For 4 kHz, sound threshold (in dB) *mc* and mc-*Pz1*^*MU*^ lines one-way ANOVA, F(2,33) = (21) *p* = *1.4X10*^−*6*^. *Post hoc* comparisons using the Tukey HSD test indicate that at 4-w (4 kHz), *mc* (51±9, n=16) vs. *mc-Pz1*^*MU*^ (74±15, n=12) (*p*=*2.3X10*^−*5*^); at 8-w *m-c* (55±4, n=14) vs. *mc-Pz2*^*MU*^ (78±9, n=8) (*p*=*1.5X10*^−*5*^) are significantly different. For 8 kHz, *mc* and *mc-Pz1*^*MU*^ lines one-way ANOVA, F(2,28) = (20) *p* = *4.6X10*^−*6*^*. Post hoc* comparisons using the Tukey HSD test indicate that at 4-w (8 kHz), *mc* (43±13, n=12) *vs. mc-Pz1*^*MU*^ (65±16, n=12) *p*=*5.0X10*^−*4*^; at 8-w *mc* (45±6, n=8) *vs*. mc-*Pz1*^*MU*^ (79±7, n=7) *p*=*5.9X10*^−*6*^ are significantly different. For 16 kHz, *mc* and mc-*Pz1*^*MU*^ lines one-way ANOVA, F(2,27) = (56) *p* = *2.5X10*^−*10*^. *Post hoc* comparisons using the Tukey HSD test indicate that at 4-w (16 kHz), *mc* (31±8, n=10) *vs. Pz1-ki*^*m-c*^ (59±10, n=12) (*p*=2.5X10^−10^); at 8-w *mc* (30±2, n=9) *vs*. mc-*Pz1*^*MU*^ (76±10, n=8) (*p*=*1.2X10*^−*12*^) are significantly different. For 32 kHz, *mc* and mc-*Pz1*^*MU*^ lines one-way ANOVA, F(2,34) = (9) *p* = *6.5X10*^−*4*^. *Post hoc* comparisons using the Tukey HSD test indicate that at 4-w (32 kHz), *mc* (71±9, n=16) *vs*. mc-*Pz1*^*MU*^ (82±11, n=12) (*p*=*8X10*^−*3*^); at 8-w *mc* (76±2, n=7) *vs*. mc-*Pz1*^*MU*^ (86±5, n=9) (*p*=*1.3X10*^−*3*^) are significantly different. **b,** Using similar assessment for *mc* and *mc-Pz2*^*MU*^ 4 kHz, sound threshold (in dB) *mc* and *mc-Pz2*^*MU*^ lines one-way ANOVA, F(2,43) = (39) *p* = *2.1X10*^−*10*^. *Post hoc* comparisons using the Tukey HSD test indicate that at 4-w (4 kHz), *mc* (56±11, n=17) *vs. mc-Pz2*^*MU*^ (80±10, n=20) *p*=*1.0X10*^−*20*^; at 8-w *m-c* (55±6, n=16) *vs. mc-Pz2*^*MU*^ (85±6, n=9) p=*1.1X10*^−*20*^ are significantly different. For 8 kHz, *mc* and *mc-Pz2*^*MU*^ lines one-way ANOVA, F(2,38) = (24) *p* = 2.3X10^−7^. *Post hoc* comparisons using the Tukey HSD test indicate that at 4-w (8 kHz), *mc* (46±14, n=12) *vs. mc-Pz2*^*MU*^ (77±15, n=20) (*p*=*6.8X10*^−*7*^); at 8-w *mc* (45±14, n=12) *vs. mc-Pz2*^*MU*^ (79±9 n=9) (*p*=*5.9X10*^−*6*^) are significantly different. For 16 kHz, *mc* and *mc-Pz2*^*MU*^ lines one-way ANOVA, F(2,37) = (44) *p* = *1.6X10*^−*10*^. *Post hoc* comparisons using the Tukey HSD test indicate that at 4-w (16 kHz), *mc* (30±7 n=11) *vs. mc-Pz2*^*MU*^ (68±17, n=20) (*p*=1X10–20); at 8-w *mc* (29±3, n=11) *vs. mc-Pz2*^*MU*^ (86±5, n=9) *p*=*1.1X10*^−*20*^ are significantly different. For 32 kHz, *mc* and *mc-Pz2*^*MU*^ lines one-way ANOVA, F(2,41) = (16) *p* = *7.4X10*^−*6*^. *Post hoc* comparisons using the Tukey HSD test indicate that at 4-w (32 kHz), *mc* (69±9, n=17) *vs. mc-Pz2*^*MU*^ (82±9, n=18) *p*=*8.9X10*^−*5*^; at 8-w *m-c* (72±2, n=13) *vs. mc-Pz2*^*MU*^ (86±5, n=9) *p*=*5.4X10*^−*5*^ are significantly different. **c**, ABR thresholds *mc* and double knockin mice, *mc-Pz1–2*^*MU*^ at 4 kHz, sound threshold (in dB) *mc* and *mc-Pz1–2*^*MU*^ lines one-way ANOVA, F(2,24) = (50) *p* = *2.9X10*^−*9*^. *Post hoc* comparisons using the Tukey HSD test indicate that at 4-w (4 kHz), *mc* (57±9, n=9) *vs. mc-Pz1–2*^*MU*^ (83±7, n=9) *p*=*7.8X10*^−*8*^; at 8-w *m-c* (56±10, n=9) *vs. mc-Pz1–2*^*MU*^ (88±4, n=9) p=*1.0X10*^−*20*^ are significantly different. For 8 kHz, *mc* and *mc-Pz1–2*^*MU*^ lines one-way ANOVA, F(2,24) = (31) *p* = 2.0X10^−7^. *Post hoc* comparisons using the Tukey HSD test indicate that at 4-w (8 kHz), *mc* (48±15, n=9) *vs. mc-Pz1–2*^*MU*^ (78±8, n=9) (*p*=*4.9X10*^−*6*^); at 8-w *mc* (47±13, n=9) *vs. mc-Pz1–2*^*MU*^ (83±6 n=9) (*p*=*3.7X10*^−*7*^) are significantly different. For 16 kHz, *mc* and *mc-Pz1–2*^*MU*^ lines one-way ANOVA, F(2,24) = (270) *p* = *3.6X10*^−*17*^. *Post hoc* comparisons using the Tukey HSD test indicate that at 4-w (16 kHz), *mc* (27±7 n=9) *vs. mc-Pz1–2*^*MU*^ (81±7, n=9) (*p*=*1X10*^−*20*^); at 8-w *mc* (29±3, n=9) *vs. mc-Pz1–2*^*MU*^ (86±3, n=9) *p*=*1.1X10*^−*20*^ are significantly different. For 32 kHz, *mc* and *mc-Pz1–2*^*MU*^ lines one-way ANOVA, F(2,24) = (23) *p* = 2.*3X10*^−*6*^. *Post hoc* comparisons using the Tukey HSD test indicate that at 4-w (32 kHz), *mc* (72±8, n=8) *vs. mc-Pz1–2*^*MU*^ (86±5, n=9) *p*=*7.7X10*^−*5*^; at 8-w *m-c* (74±2, n=9) *vs. mc-Pz1–2*^*MU*^ (89±2, n=9) *p*=*3.1X10*^−*6*^ are significantly different. For click, *mc* and *mc-Pz1–2*^*MU*^ lines one-way ANOVA, F(2,24) = (132) *p* = *1.2X10*^−*13*^. *Post hoc* comparisons using the Tukey HSD test indicate that at 4-w (click), *mc* (46±7 n=9) *vs. mc-Pz1–2*^*MU*^ (83±7, n=9) (*p*=*1X10*^−*20*^); at 8-w *mc* (49±5, n=9) *vs. mc-Pz1–2*^*MU*^ (87±3, n=9) *p=1.1X10*^−*21*^ are significantly different.
**Supplement Figure 18 (S18). Reduced FM1–43 uptake in *Pz*^*MU*^ hair cells** **a,** Fluorescent images of FM1–43 were taken at different time points at three focal levels, L1, L2, and L3, referring to the apical, cuticular plate, and basal levels, respectively. The frames shown are at L2 (cuticular plate). Time 0 indicates the onset of dye application (10 μM for the 5-sec duration). Consecutive images of the bundles at stereocilia, cuticular plate, and basal levels were taken in 5-second intervals. The dye enters the apical aspects of the cell before being visualized at the basal pole. (Scale bar, 10 μm). **b,** The change in fluorescence at L2 (cuticular plate) focal levels as a function of time (adjusted for the interval between frame capture at each level). Densitometric data of mean pixel intensity were measured in arbitrary grayscale units (a.u) as described^19^. The number of animals tested is indicated for controls and genotypes. Frames were taken at the three rows of OHCs at the apical one-third of the cochlea, and a similar loading pattern is observed at the middle third of the cochlea. The change in fluorescence was fitted with an exponential function, and the time constants (τ, in secs) of FM1–43 dye loading in control, *mc-Pz1*^*MU*^, and *mc-Pz2*^*MU*^ apical cochlear OHCs at L2, 23±3 (n = 5), 61±6 (n = 4) and 58±8 (n = 4).
**Supplement Figure 19 (S19). Cellular degeneration of *Pz***^***MU***^ **mice. a,** Whole-mount cochlea of a ~6-week-old *Calb2-Cre (cc)* control mouse showing the low-frequency segment at the ~6-kHz cochlear region. Myo7a, the hair cell marker, is stained (white), and the stereocilia marker is stained (green) for actin. Scale bar = 10 μm. **b,** The high-frequency segment at the ~32-kHz cochlear region. ABR thresholds for the mouse were (dB); 4-kHz=40, 8-kHz=20 dB, 16-kHz=15, 32-kHz=60, and click=35. **c-d,** A 6-week-old cc-*Pz2*^*MU*^ cochlea shows 6 and 32 kHz segments. Note enlarged IHCs (red arrows) at the low-frequency ~6-kHz segment and lost OHCs (* in red) at the high-frequency ~32-kHz segment. Recorded ABR thresholds for the mouse were (dB); 4-kHz=60, 8-kHz=45, 16-kHz=85, 32-kHz=90, and click=55. Scale bar = 10 μm.
**Supplement Figure 20 (S20). Hair cell loss and degeneration in *Pz***^***MU***^ **mice as observed with SEM at P56. a,** The mc-Pz2^MU^ cochlear apex reveals near-normal IHCs and degenerating OHCs. **b-c,** The degenerating OHCs have fused stereocilia or progressive loss. **d,** Profound IHC and OHC loss in the *Pz*^*MU*^ cochlear base. **e-f,** There are only a few remaining IHCs. Scale bar=10 μm and 2 μm (**b,c**).
**Supplement Figure 21 (S21). Voltage-dependent nonlinear capacitance (NLC) in OHCs in *Myo15-Cre (mc)* and *mc-Pz1/2***^***MU***^ **mice. a**, Upper panels show representative traces of NLC recordings using a voltage stair protocol ranging from −150 to +100 mV with 10-mV increments. Left panel (control (black)), myo15-cre (m-c), middle panel (mc-*Pz1*^*MU*^ (blue)), and right panel (*mc-Pz2*^*MU*^) (red)). **b,** Normalized NLC, plotted as a function of voltage and fitted with the first derivative of a Boltzmann function describing nonlinear charge movement^78^. For OHCs cells from *mc*, *mc-Pz1*^*MU*^, and mc-*Pz2*^*MU*^ mice (n = 5 for each group). NLC obtained through correction for linear capacitance was plotted as a function of voltage and fitted with the first derivative of a Boltzmann function: C_m_ = C_ln_ + C_v_ = C_ln_ + (Q_max_ze/kT) × exp(−ze[V − V_h_]/kT)/(1 + exp[−ze(V − V_h_)/kT])^2^, where C_m_ is the total capacitance of the cell, C_ln_ is the linear capacitance, C_v_ is the nonlinear capacitance, V is the membrane potential, V_h_ is the voltage at half-maximal nonlinear charge transfer, e is the electron charge, k is Boltzmann’s constant, T is the absolute temperature, z is the valence, and Q_max_ is maximum nonlinear charge transfer. For the plots shown, values for *myo15-cre* (*mc*; controls), (C_ln_ (pF), z, Q_sp_ (fC/pF) and V_h_ (mV) were (9, −0.9, 120, −47); *mc-Pz1*^*MU*^, (7, −0.8, 98, −23); and *mc-Pz2*^*MU*^, (10, −0.9, 50, −40). **c,** Normalized NLC illustrates the shifts in V_h,_ comparing controls with the *Pz*^*MU*^ OHCs.
**Supplement Figure 22 (S22). Original gels showing Pz1/2 exists in a complex with Tmc1/2 in cochlear tissue and forms a protein complex with MET complex proteins (see Fig. 7). a**. Immunoblots of GFP input and tdTomato and mCherry after IP. **b,** Co-immunoprecipitation of FLAG-tagged Pz1 and mCherry-tagged Tmcs. Immunoprecipitation and western blot analysis were performed with anti-flag and anti-mCherry antibodies of a cell transfected with various combinations as listed (Lane 1: mCherry + Pz1-FLAG, lane 2: Tmc1-mCherry + Pz1-FLAG, lane 3: Tmc2-mCherry + Pz1-FLAG). Lane 1 showed a negative control. (**c**), Lhfpl5-Myc, and Pz1-FLAG (**d**), Pcdh15-HA and Pz1-FLAG (**e**) Cib2-V5 and Pz1-FLAG (**f**) and Tmc1- mCherry and Pz1-FLAG (**e**). Proteins were extracted from HEK 293 cells that transiently expressed target proteins. (**f**), Pull-down samples were detected using α-FLAG to identify Pz and α-mCherry to detect Tmc1 (**b**) α- His to detect Tmie (**c**), α-Myc to detect Lhfpl5 (**d**), α-HA to detect Pcdh15 (**e**), α-V5 to detect Cib2 (**f**) in western blotting.
**Supplement Figure 23 (S23). Predicted multimerization of endogenous/wild-type Pz1, Pz2, and non-functional Pz mutant subunits.** Schematic of expected channel subunit combinations with wild-type (WT) Pz1 and AAAA mutant (MU) Pz subunits. The numbers at the bottom show the potential membrane-expressed channel populations formed from each ratio of WT and MU subunits. For a stochastic assembly of channel subunits, and assuming functional hetero- and homomeric channels and equal levels of subunit expression, ~30% of HCs may carry functional Pz subunits, i.e., ~70% carry mutant channels, whereas the number of functional HCs decreases to ~12% in the *Pz1–2*^*MU*^ cochlear HCs.
