# Supplementary material for: The Piezo channel is central to the mechano-sensitive channel complex in the mammalian inner ear
Source: Res Sq. 2023 Jul 12:rs.3.rs-2287052. Preprint. [Version 1] doi: 10.21203/rs.3.rs-2287052/v1 (PMC10371147; doi:10.21203/rs.3.rs-2287052/v1)

Supplement Figures

Supplement Figure 1

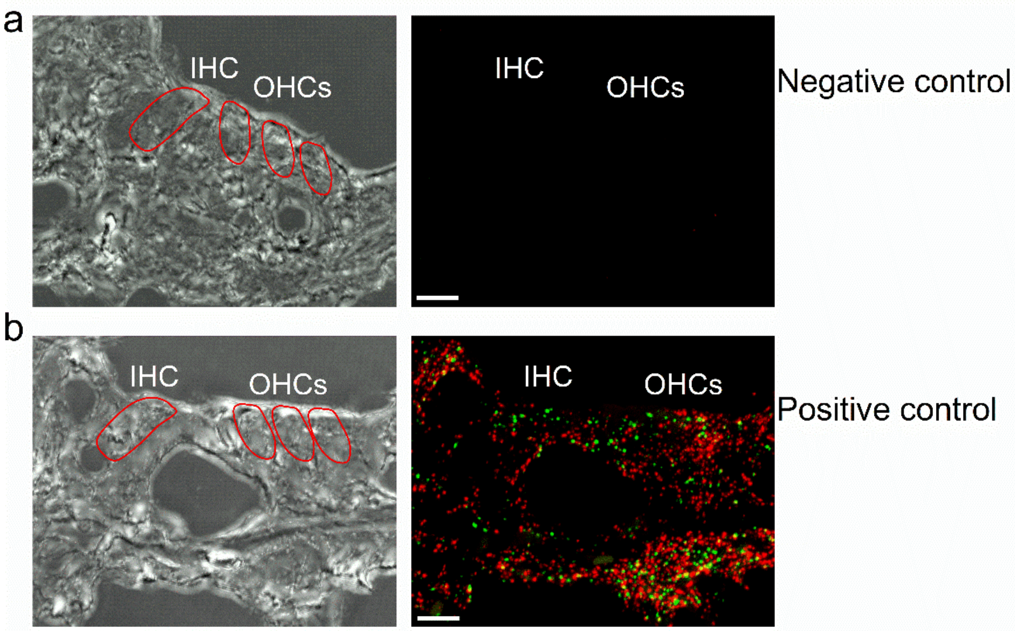

Supplement Figure 2

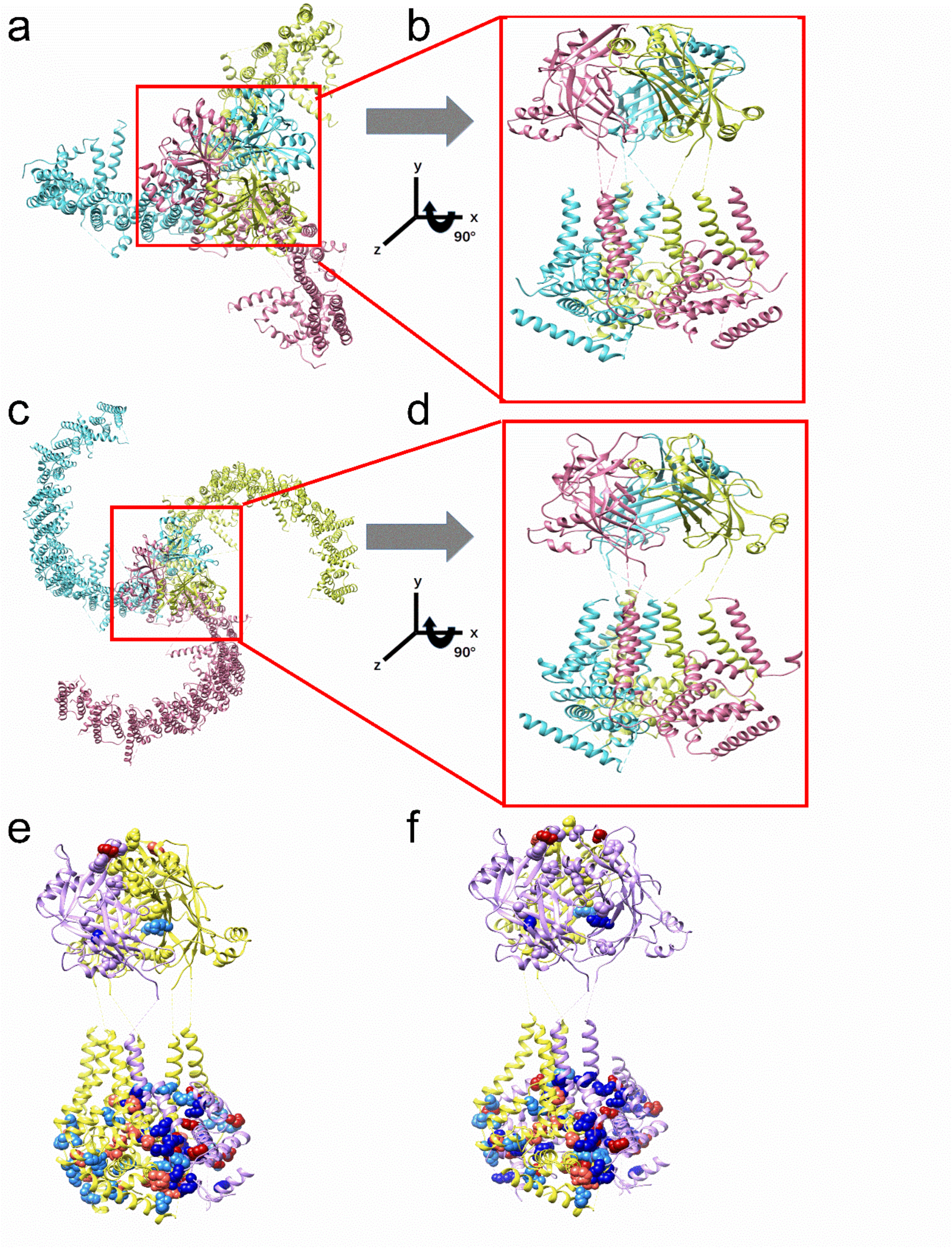

Supplement Figure 3

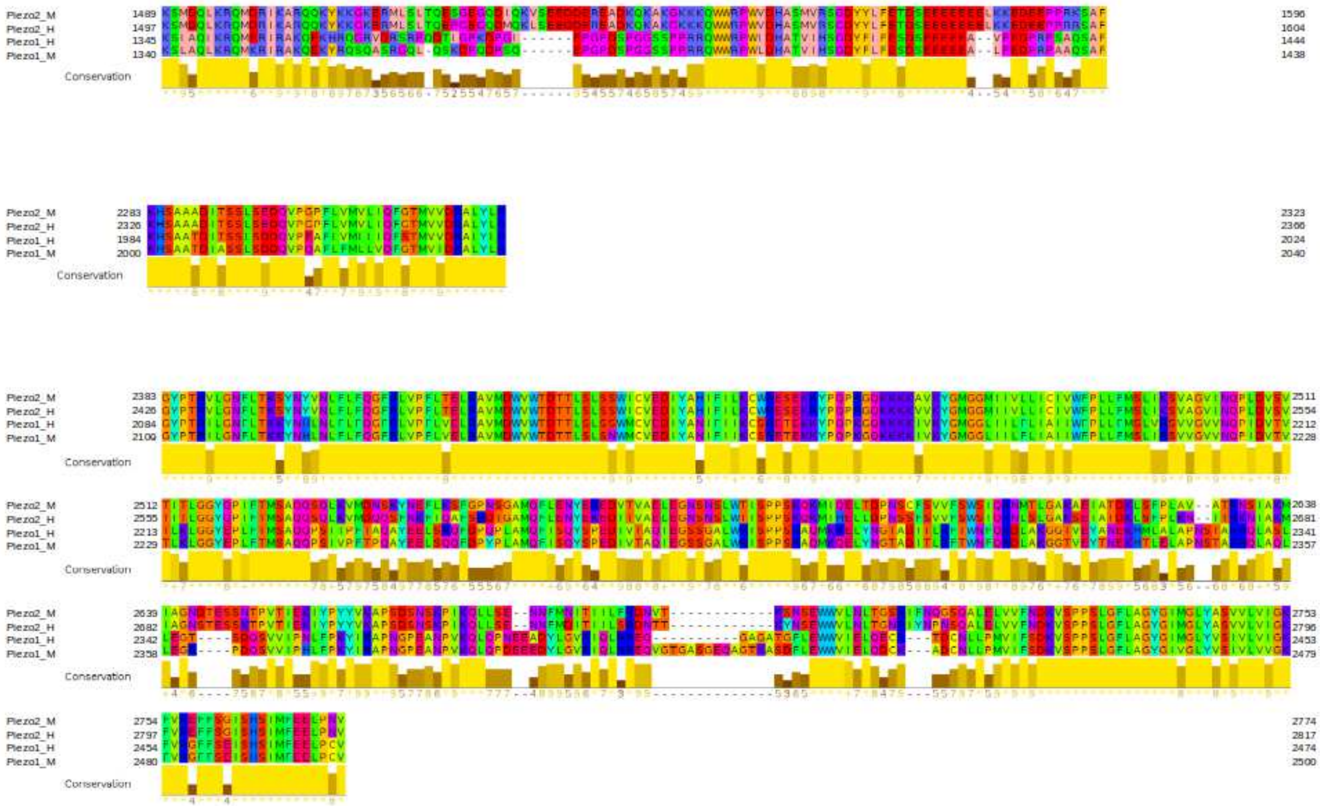

Supplement Figure 4

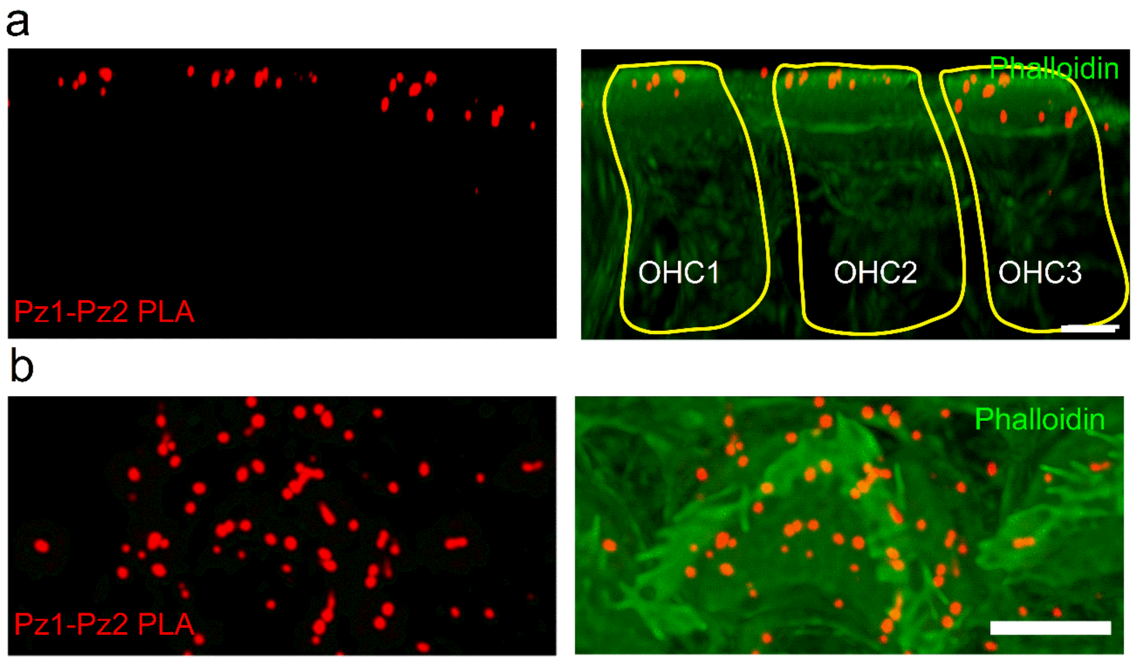

Supplement Figure 5

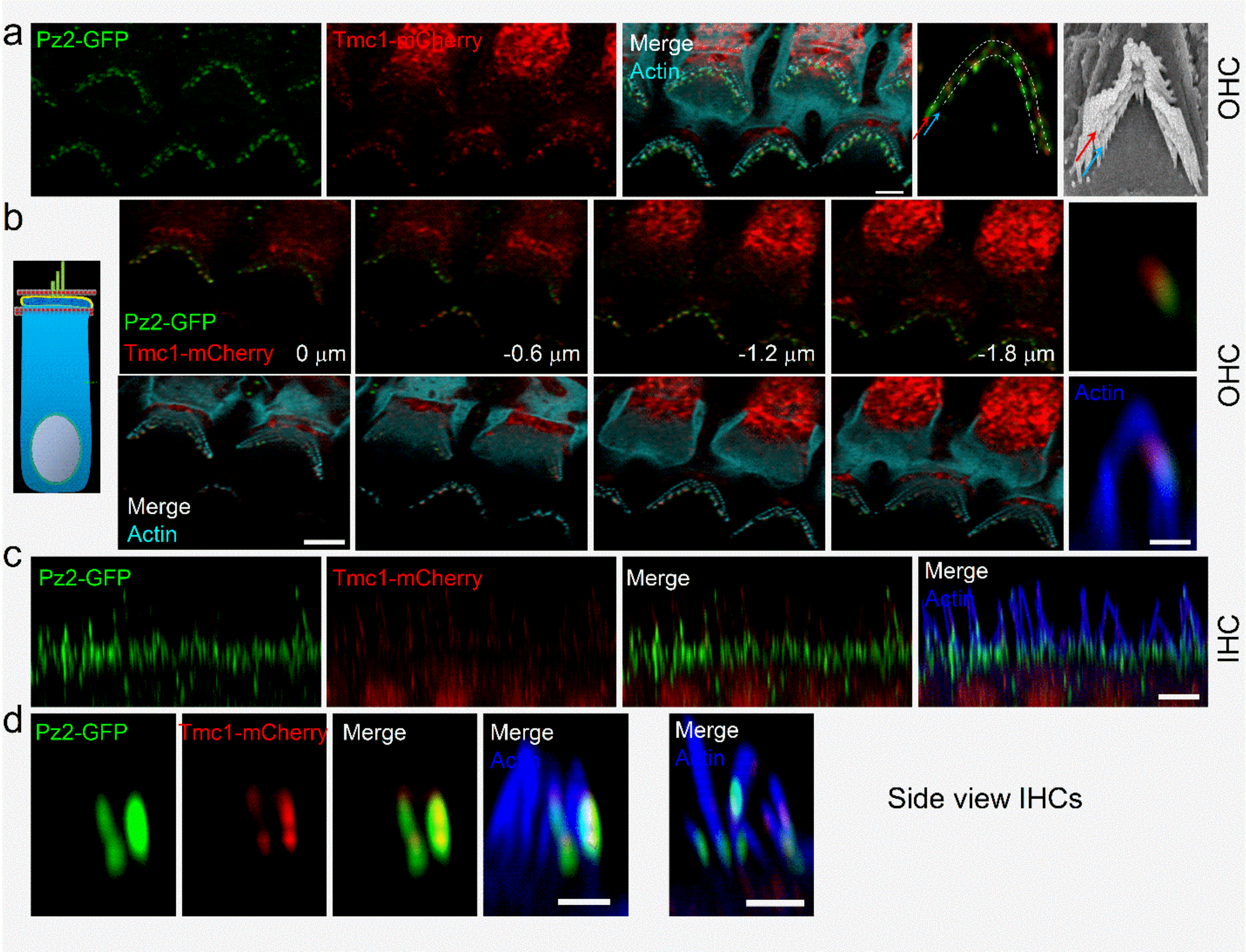

Supplement Figure 6

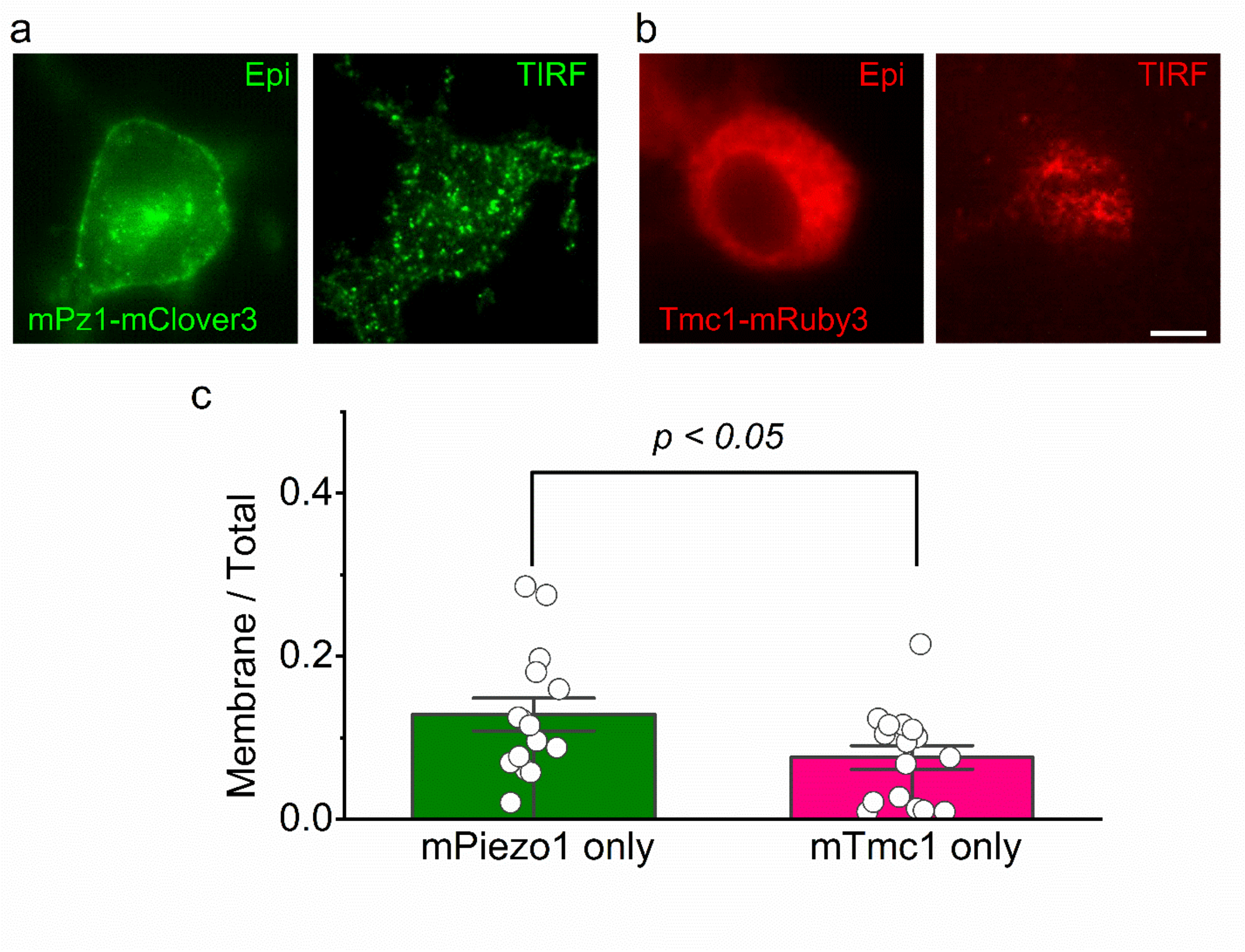

Supplement Figure 7

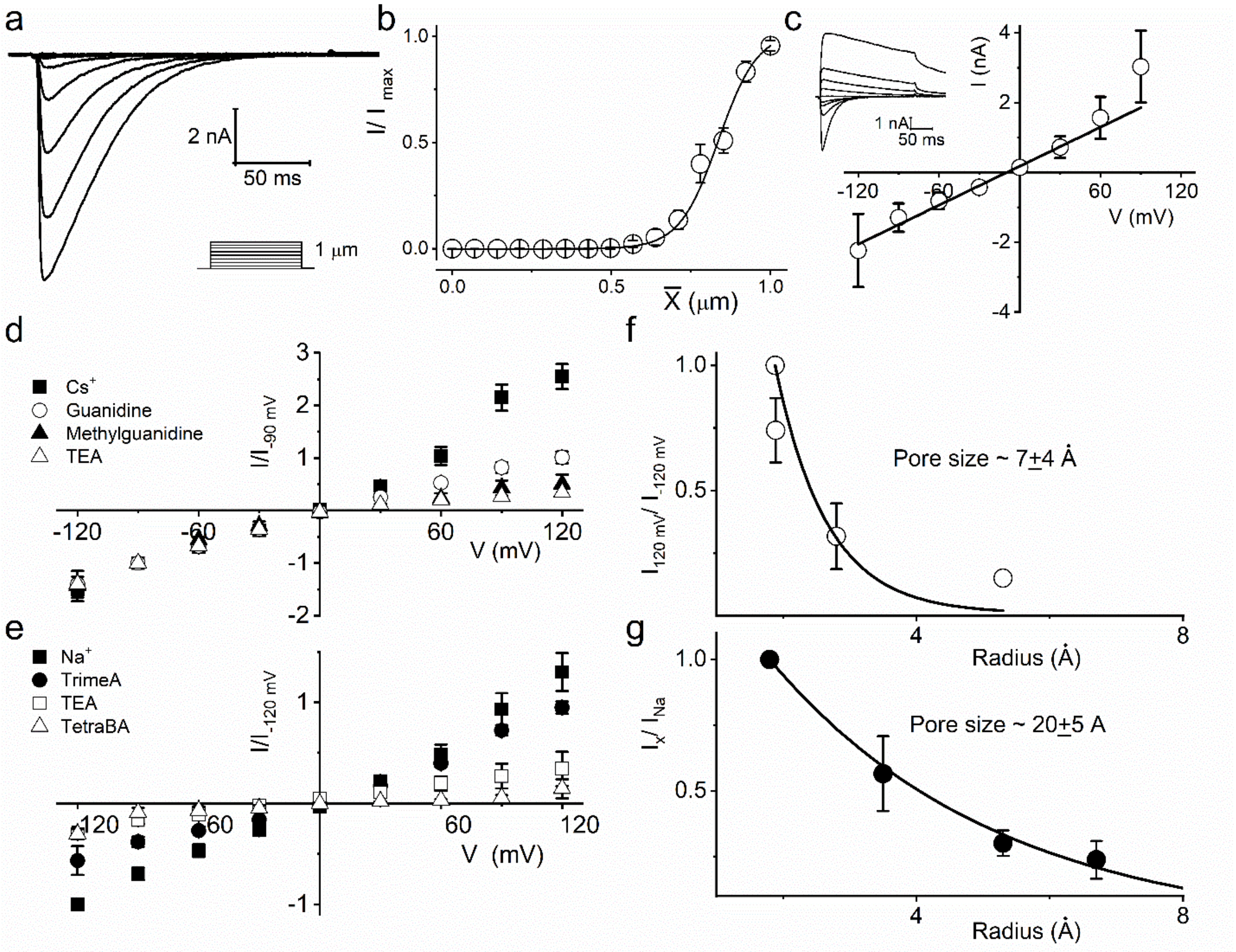

Supplement Figure 8

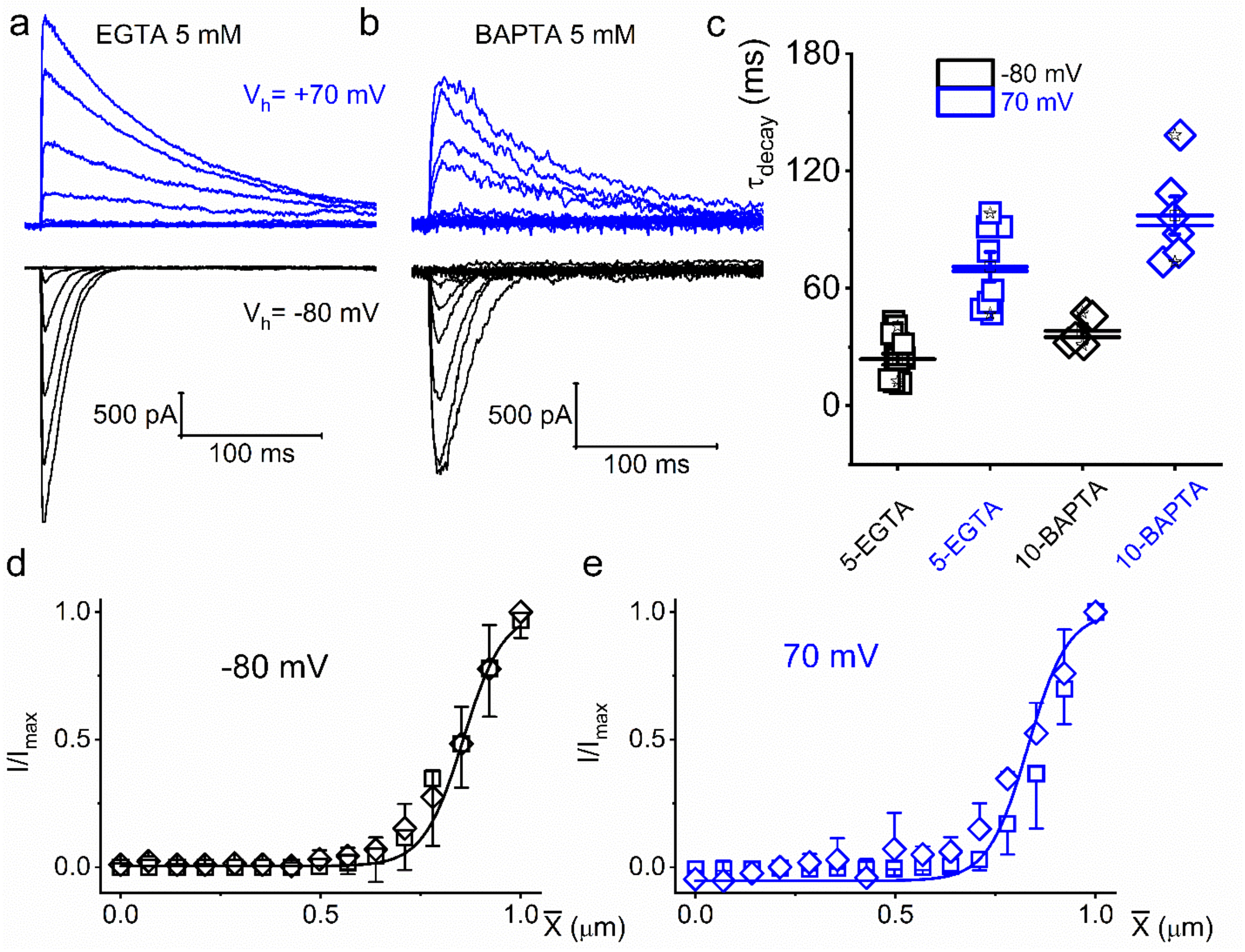

Supplement Figure 9

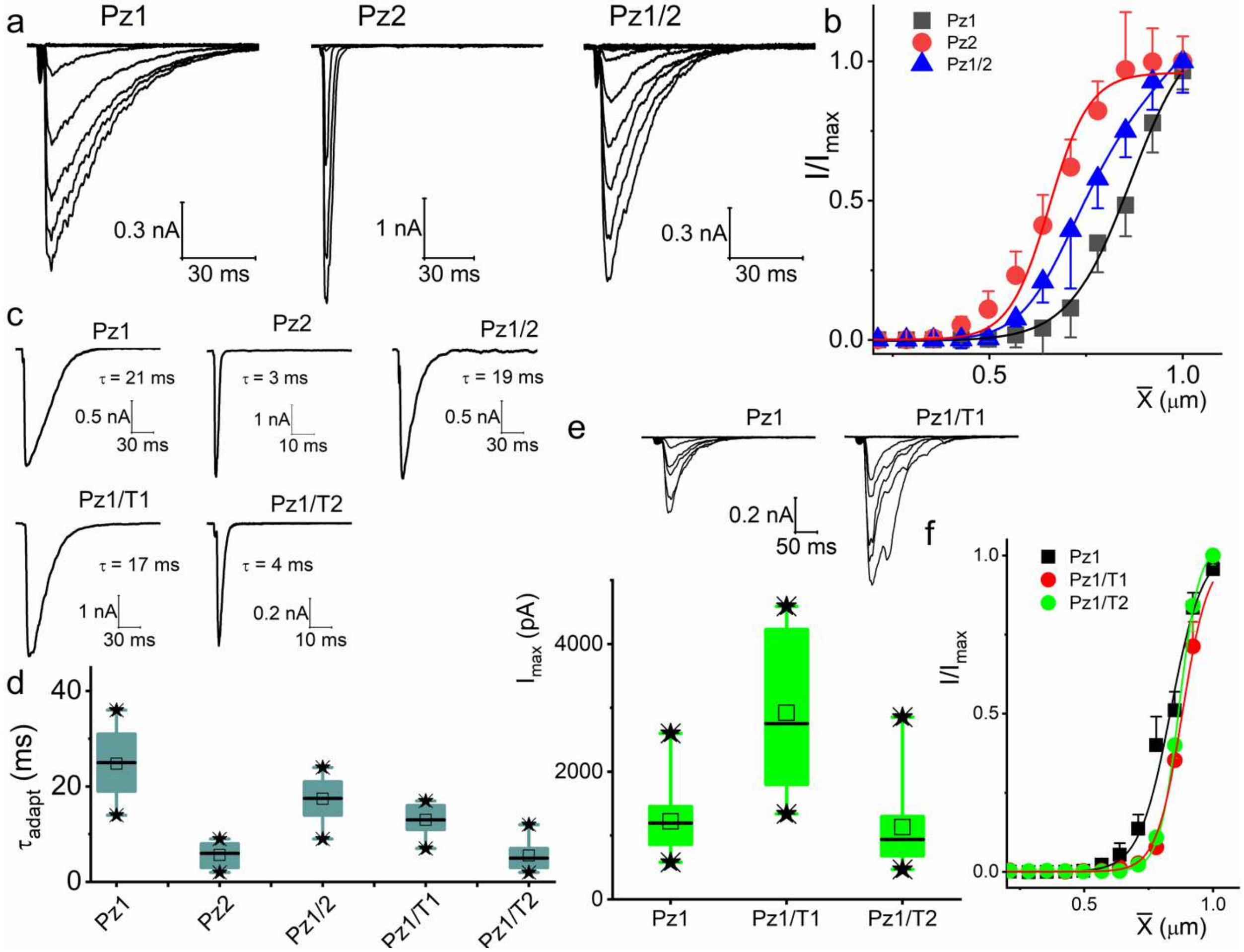

Supplement Figure 10

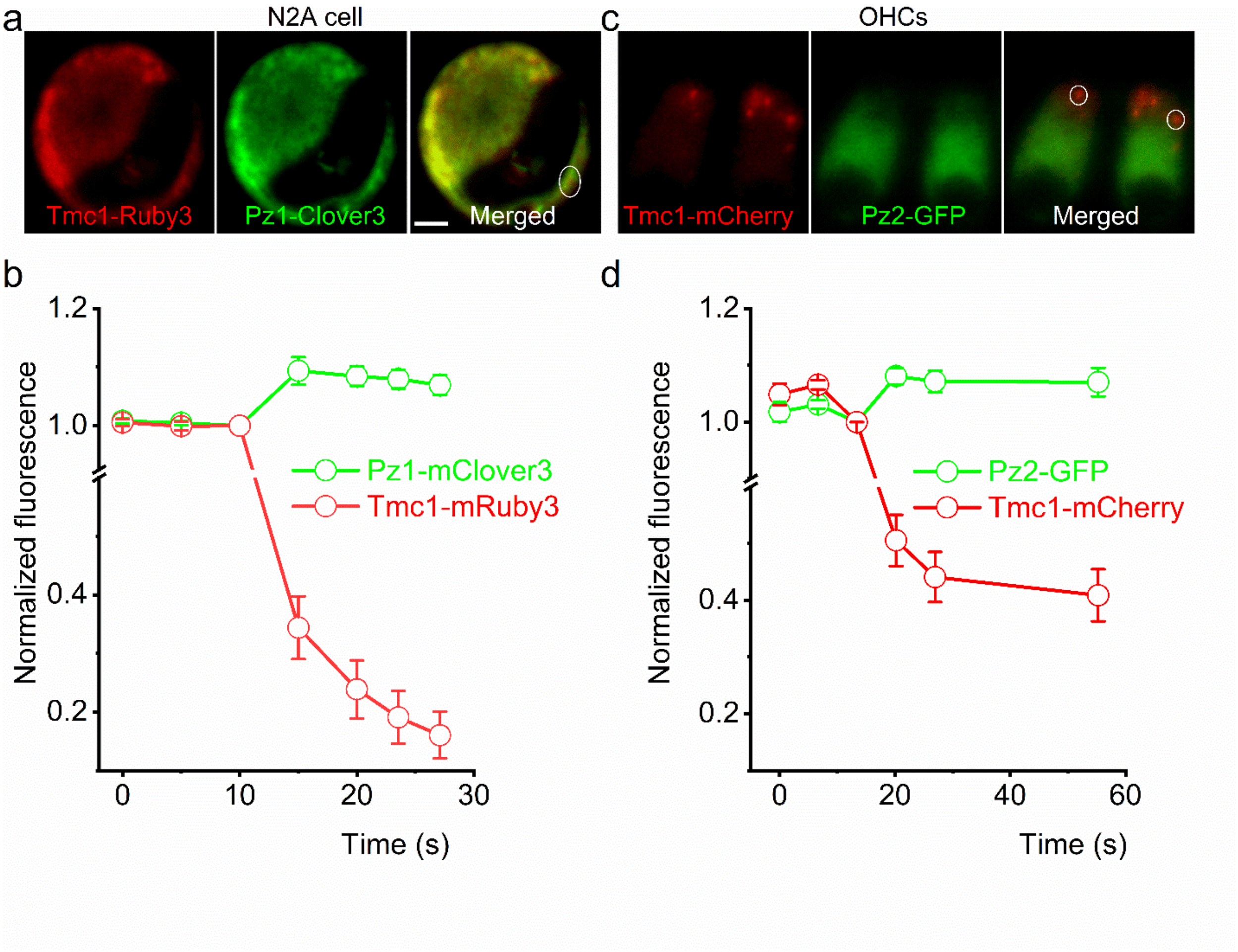

Supplement Figure 11

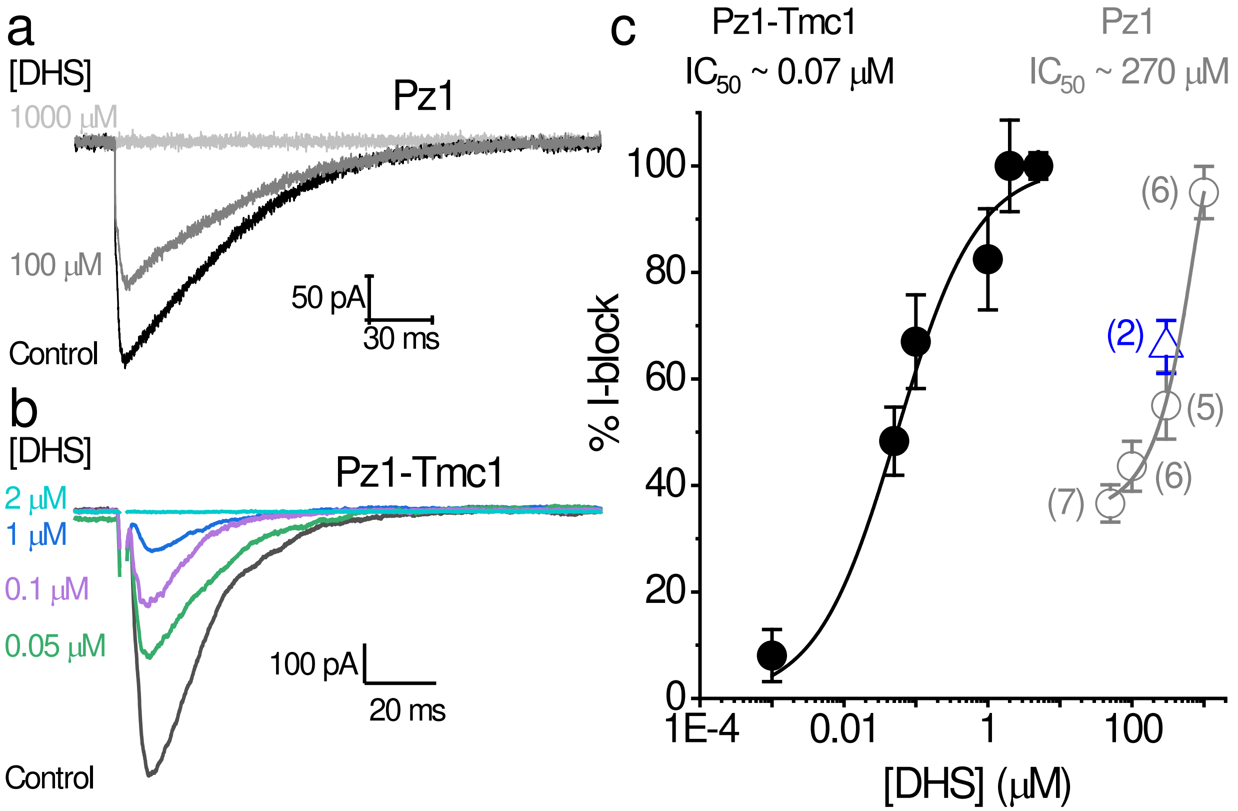

Supplement Figure 12

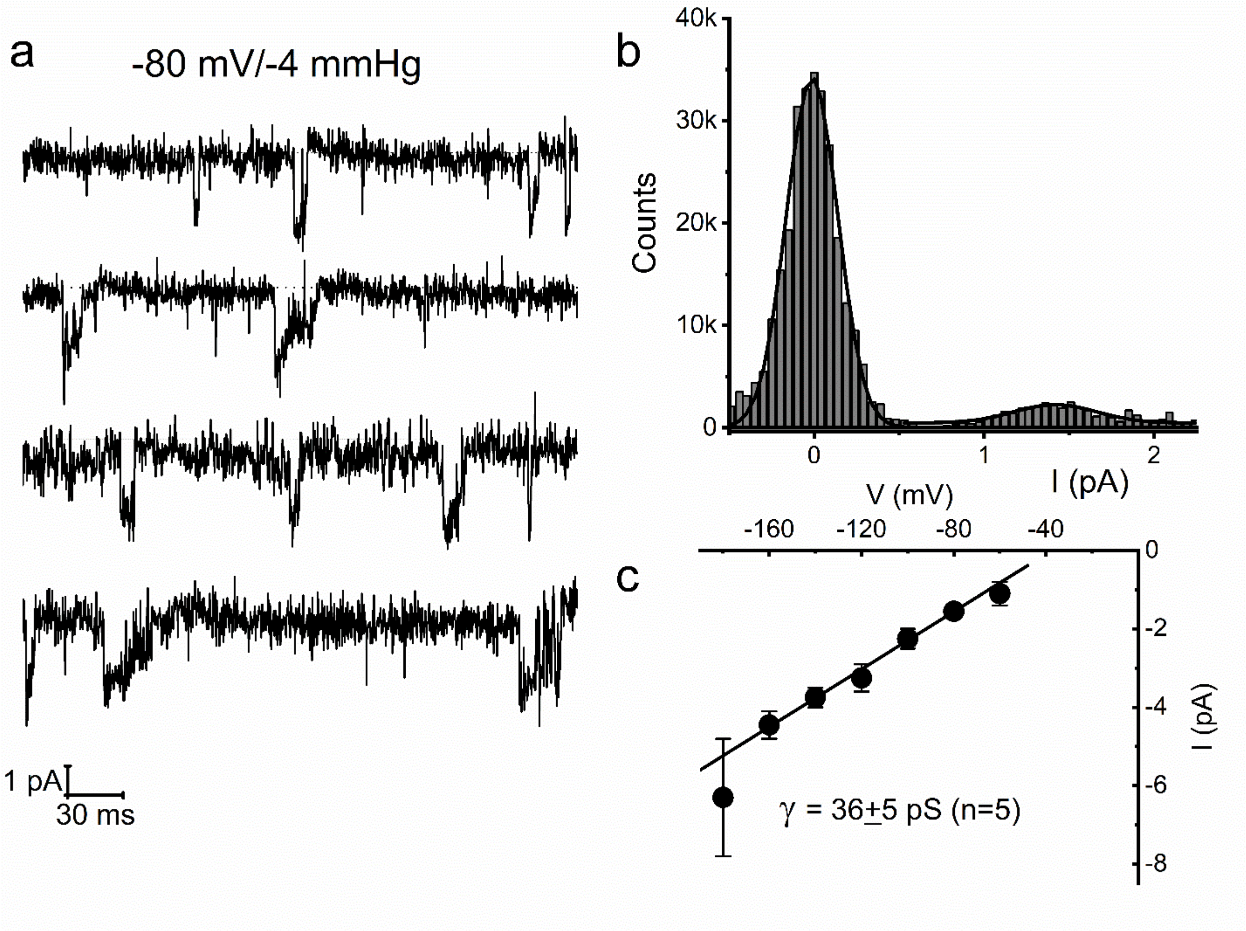

Supplement Figure 13

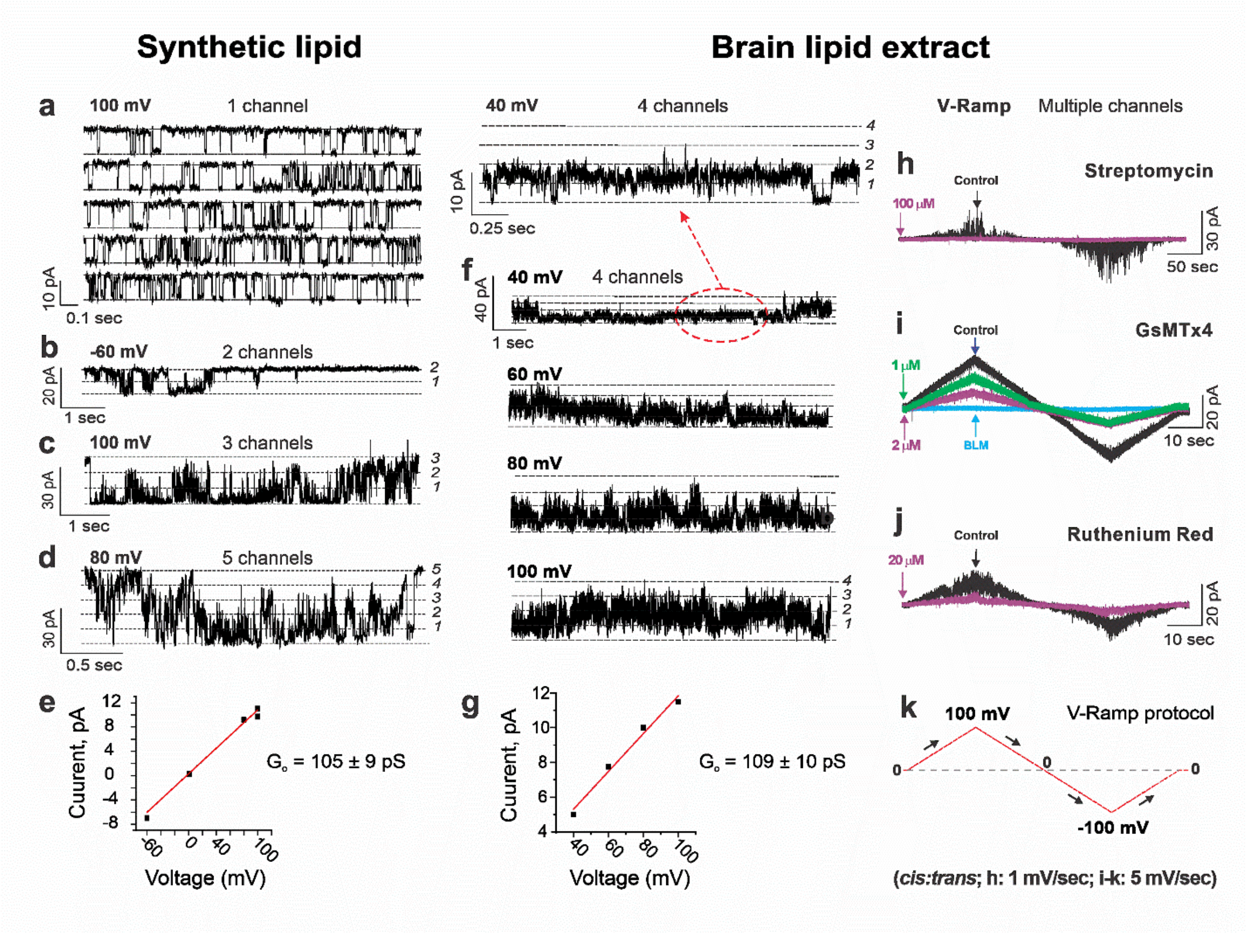

Supplement Figure 14

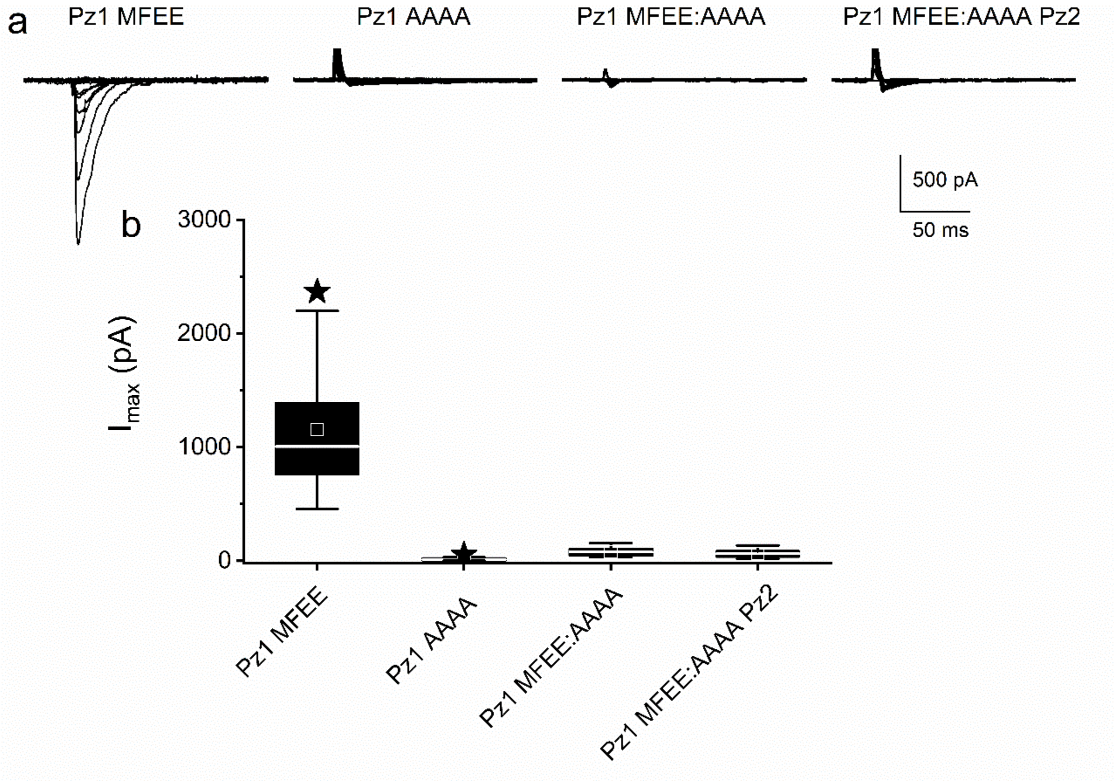

Supplement Figure 15

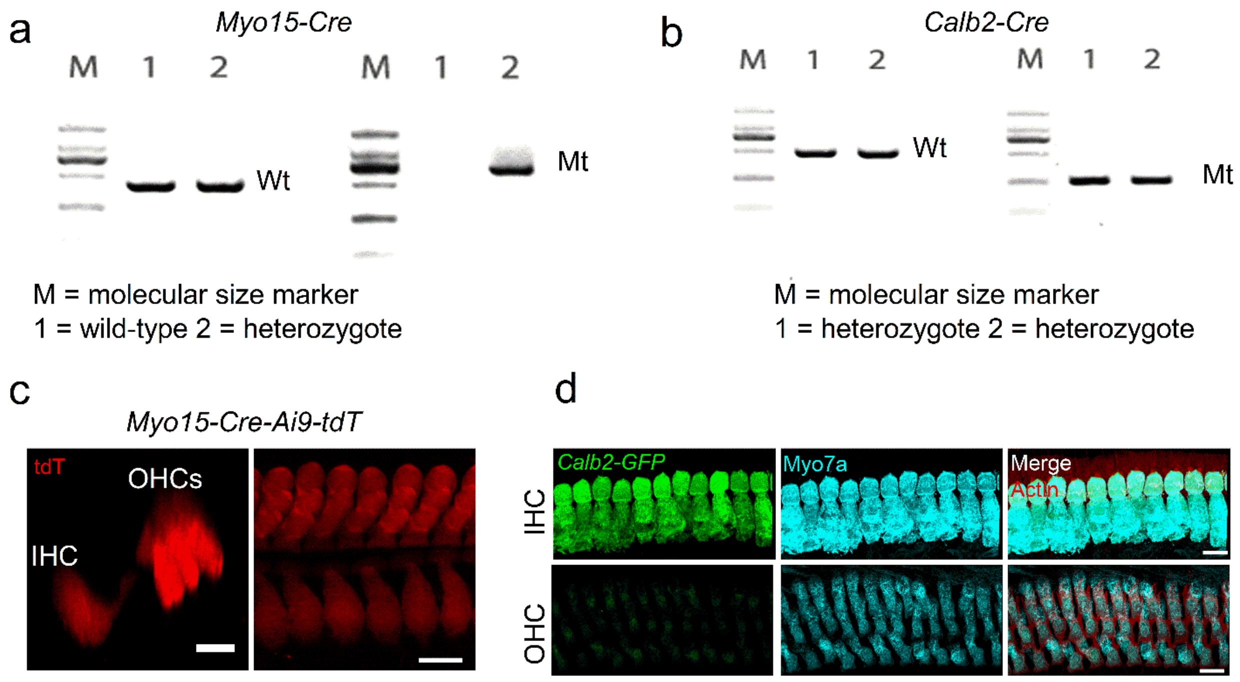

Supplement Figure 16

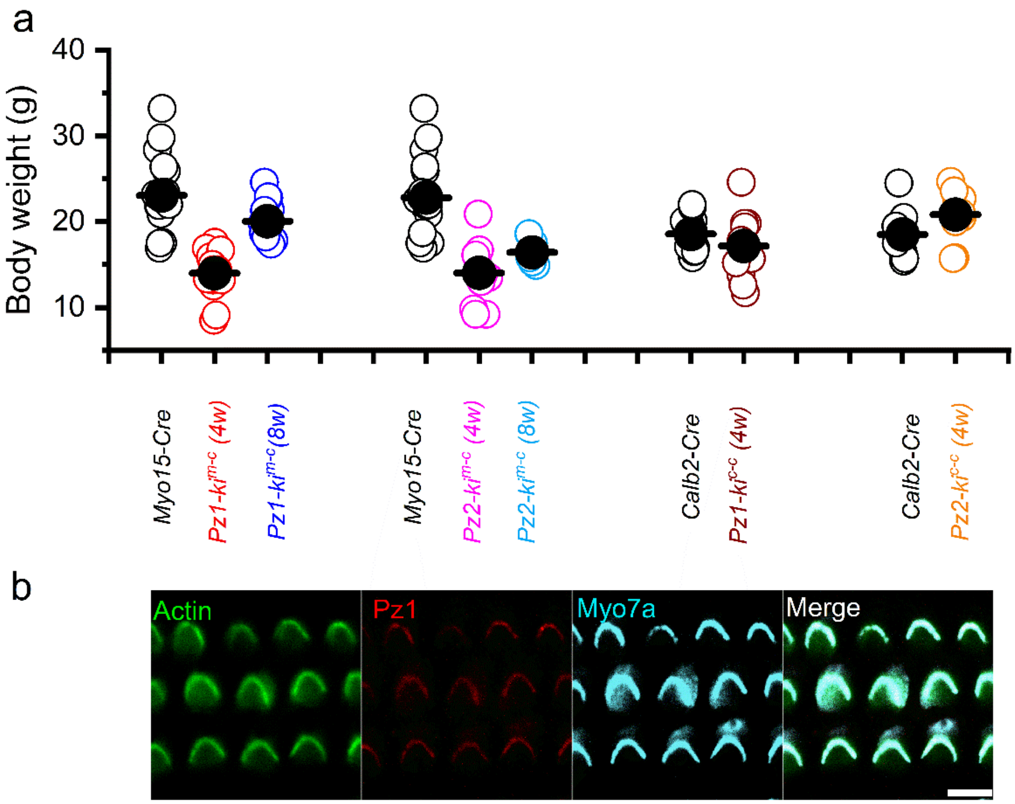

Supplement Figure 17

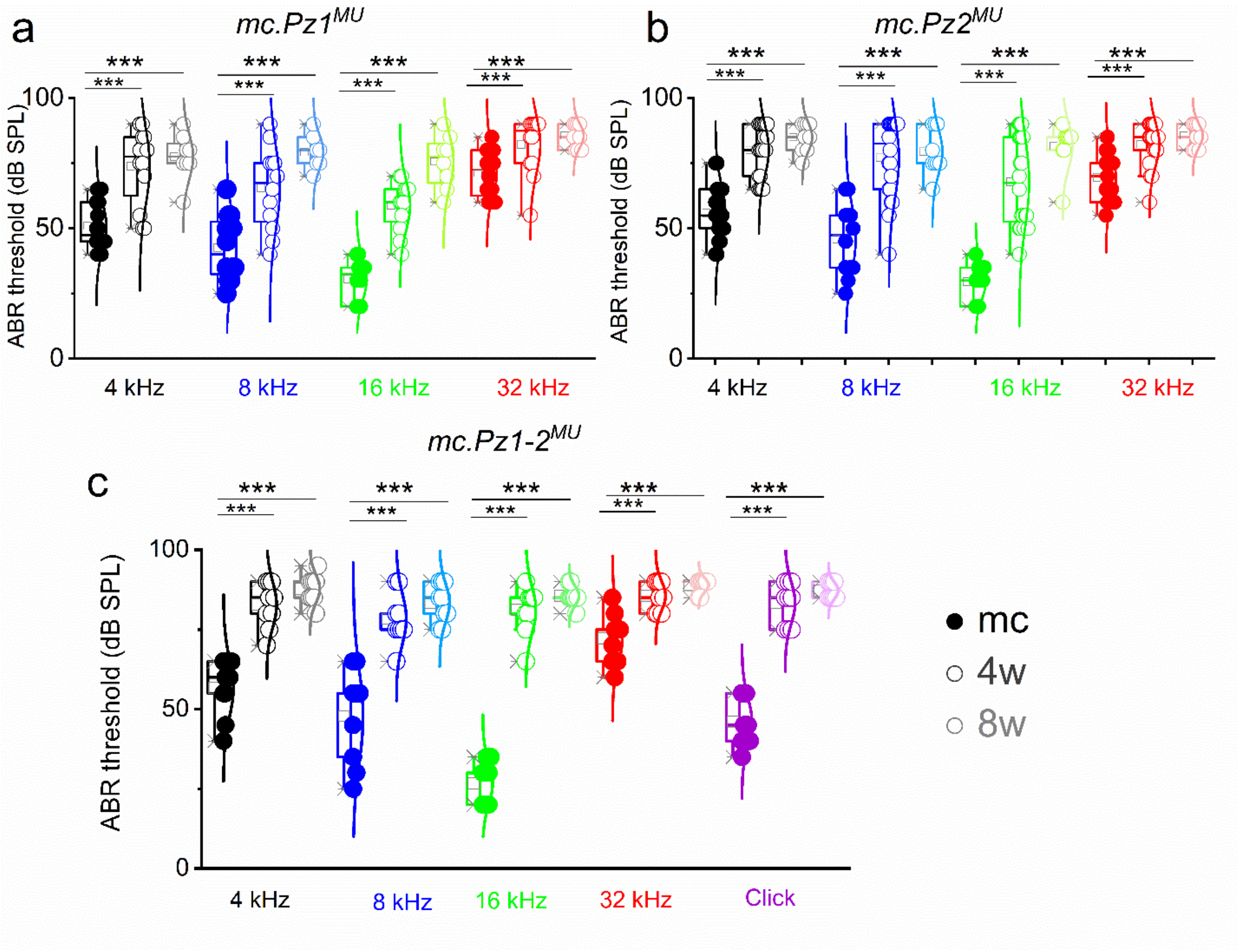

Supplement Figure 18

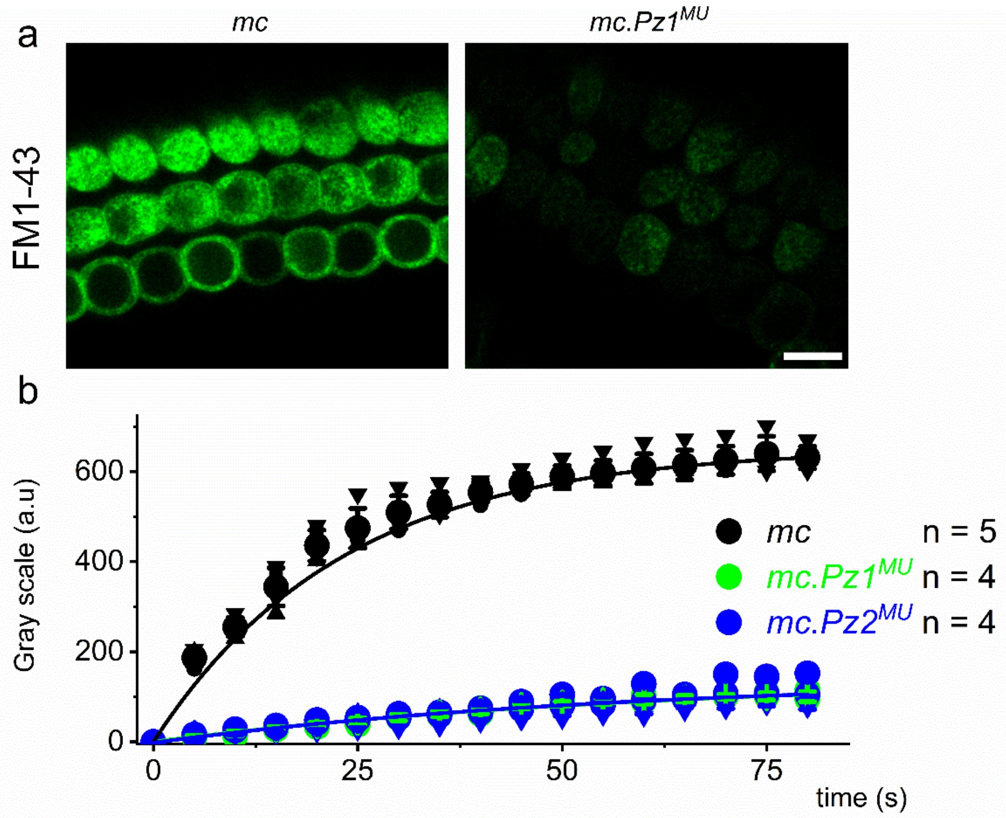

Supplement Figure 19

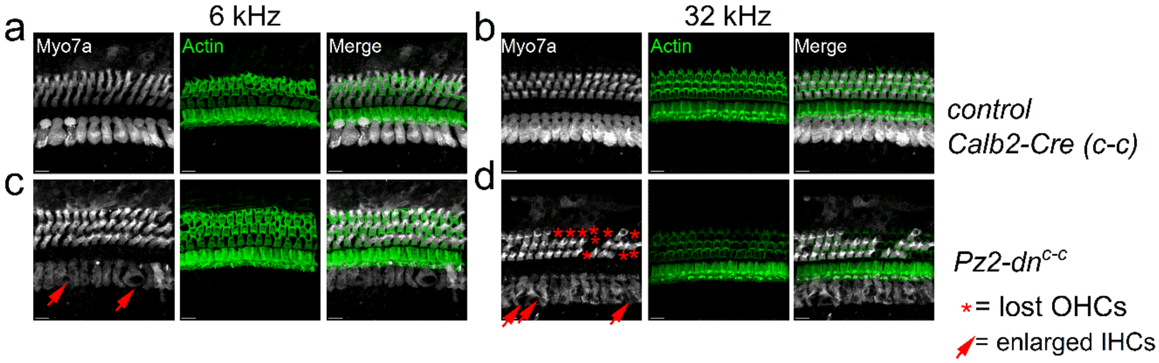

Supplement Figure 20

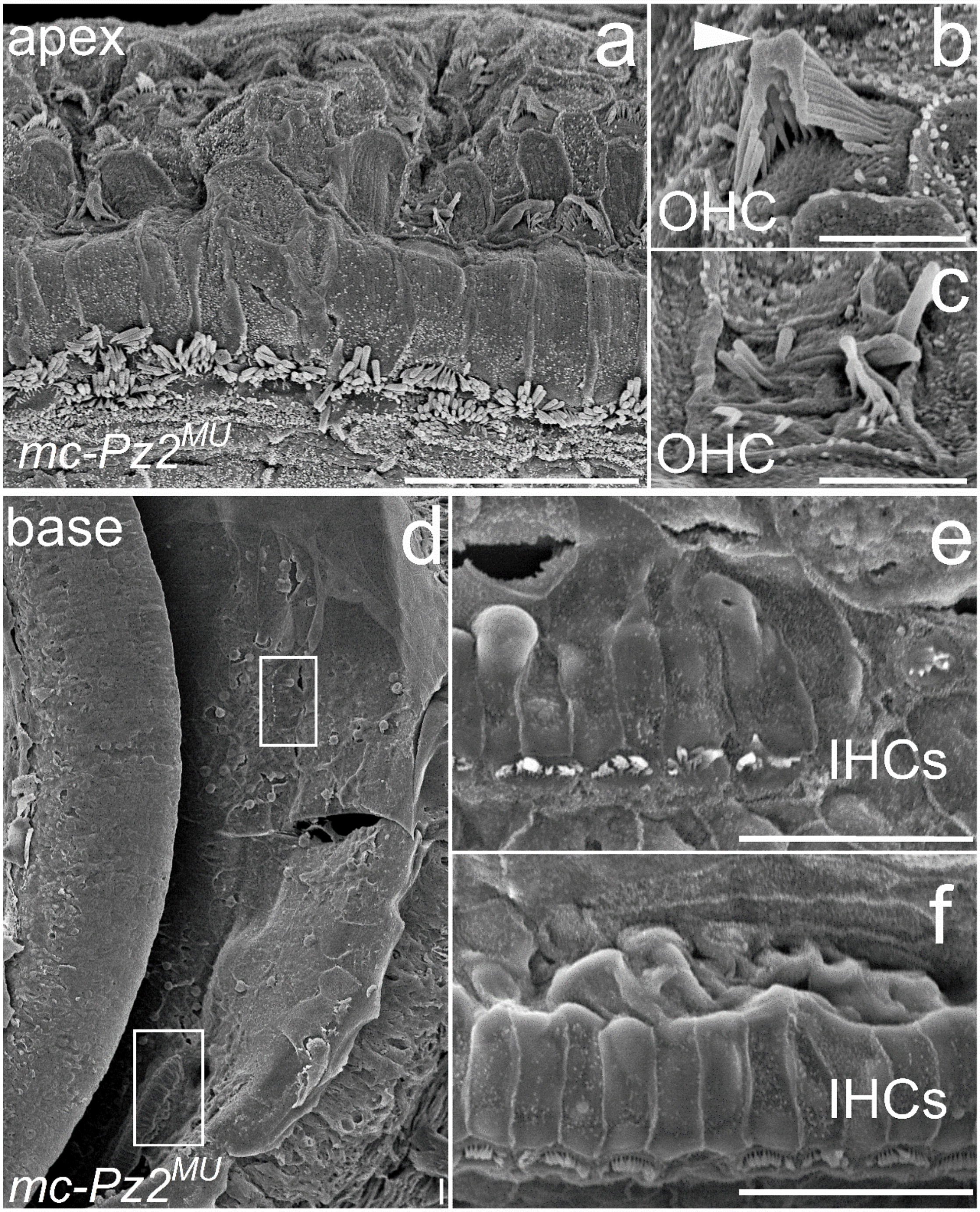

Supplement Figure 21

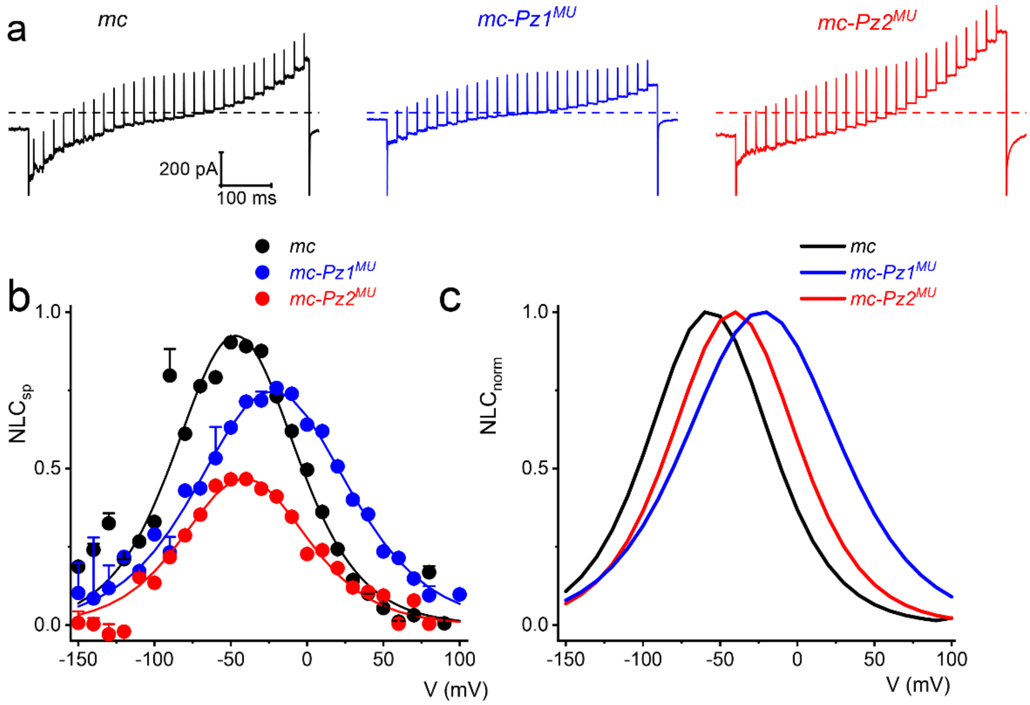

Supplement Figure 22

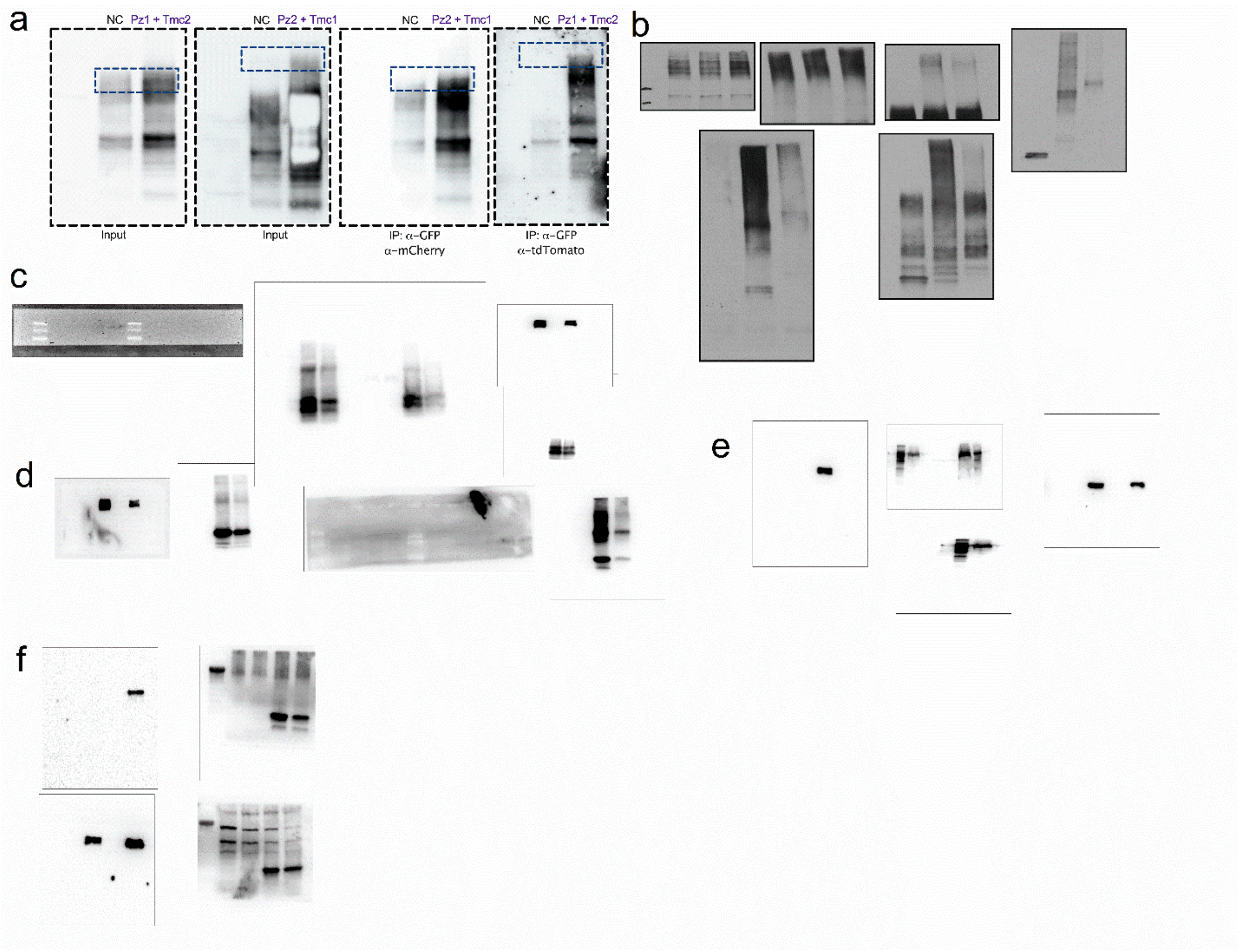

Supplement Figure 23

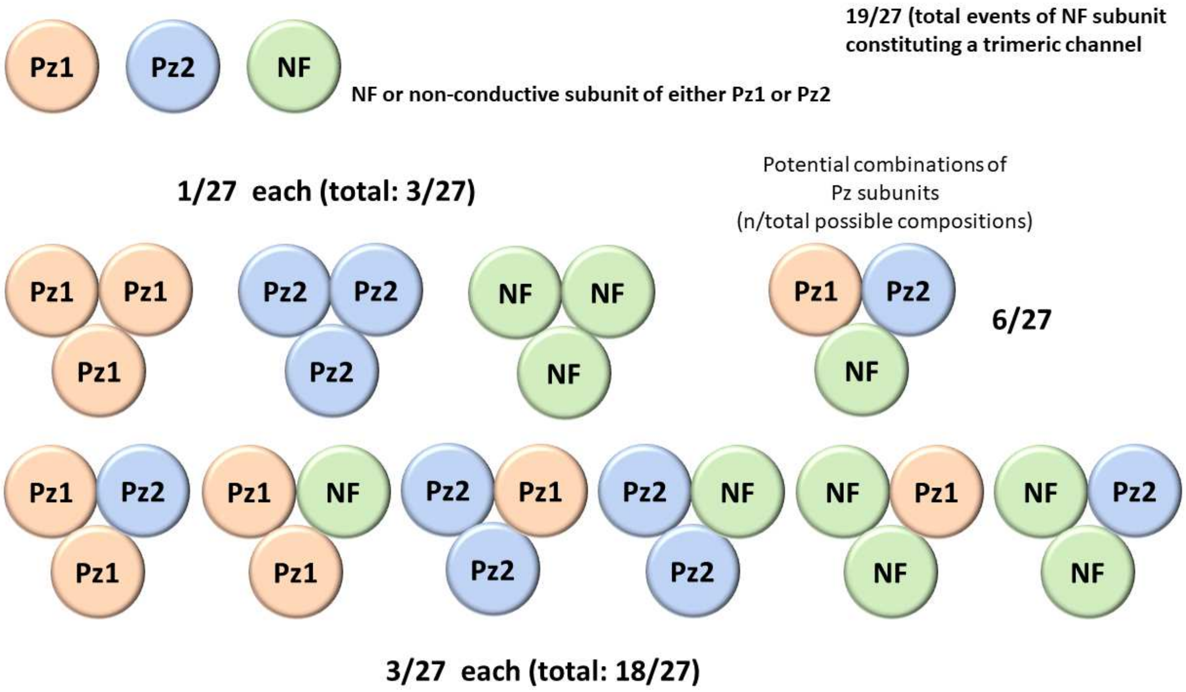

Supplement: 1 — Supplement Figure 1 (S1). Negative and positive controls in cochlear sections. smFISH localizes transcripts encoding the Pz1/2 channels and Tmc1 (Fig. 1), but not in negative control sections. a-b, Negative and positive probes provided by the manufacturer (ACD) were used on cryo-sections of the 2-w-old cochlea. Positive probes for mammalian samples were detected as fluorescent puncta in green and red. Outlines in red (left column) show one row of IHCs and three rows of OHCs. Comparison of control and experimental values are summarized in Fig. 1 and provided in the Results. Scale bar = 10 μm. Supplement Figure 2 (S2). Salt bridge specificity is highly conserved in the Pz1 and Pz2 homotrimer interfaces. a-d, Homotrimers of Pz1 (PDB: 6BPZ) from the extracellular (a) and transmembrane (b) side and Pz2 (PDB: 6KG7) from the extracellular (c) and transmembrane (d) side. Monomers are colored consistently in panels (a-d) with monomer 1 = yellow, monomer 2 = cyan, monomer 3 = pink e-f) side view of a heterotrimer with a 2:1 (e) and a 1:2 (f) Pz1: Pz2 ratio. The protein backbone is shown in a ribbon with basic and acidic amino acids in the sphere. Coloring for Pz1 is as follows: ribbon = yellow, conserved basic = light blue, conserved acidic = red/orange, non-conserved basic or acidic = same color as protein ribbon. Pz2 coloring is as follows: ribbon = purple/pink, conserved basic = dark blue, conserved acidic = dark red, non-conserved basic or acidic = same color as protein ribbon. Supplement Figure 3 (S3). Alignments of Pz1 and Pz2 interacting interfaces. Sequence alignment for Pz1 trimer interface. Alignment includes human Pz1 (Piezo1_H), mouse Pz1 (Piezo1_M), human Pz2 (Piezo2_H), mouse Pz2 (Piezo2_M). The sequence coloring is based on amino acid properties according to the “Taylor” scheme found in Jalview. Supplement Figure 4 (S4). Localization of Pz1 and Pz2 within nanometer proximity in hair cells. a-b. The expression and distribution of Pz1 and Pz2 were detected using [file NIHPPRS2287052V1-supplement-1.pdf]
